# Enhanced genome editing efficiency of CRISPR PLUS: Cas9 chimeric fusion proteins

**DOI:** 10.1038/s41598-021-95406-8

**Published:** 2021-08-10

**Authors:** Jongjin Park, Jiyoung Yoon, Daekee Kwon, Mi-Jung Han, Sunmee Choi, Slki Park, Junghyuk Lee, Kiwook Lee, Jaehwan Lee, Seunghee Lee, Kyung-Sun Kang, Sunghwa Choe

**Affiliations:** 1 G+FLAS Life Sciences, CRISPR PLUS Lab, 38 Nakseong-daero, Gwanak-Gu, Seoul, 08790 Korea; 2 Naturegenic Inc, 1281 Win Hentschel Boulevard, Kurz Purdue Technology Center Suite 1573, West Lafayette, IN 47906 USA; 3 Stem Cells and Regenerative Bioengineering Institute in Kangstem Biotech, Gwangmyeong SK TechnoPark, Gwangmyeong-si, 14322 Gyeonggi-do Korea; 4 Adult Stem Cell Research Center, College of Veterinary Medicine, Seoul National University, Seoul, 08826 Korea; 5 School of Biological Sciences, College of Natural Sciences, Seoul National University, Gwanak-gu, Seoul, 08826 Korea

**Keywords:** Biochemistry, Biotechnology, Cell biology, Stem cells, Molecular medicine

## Abstract

Efforts to improve CRISPR-Cas9 genome editing systems for lower off-target effects are mostly at the cost of its robust on-target efficiency. To enhance both accuracy and efficiency, we created chimeric SpyCas9 proteins fused with the 5′-to-3′ exonuclease Recombination J (RecJ) or with GFP and demonstrated that transfection of the pre-assembled ribonucleoprotein of the two chimeric proteins into human or plant cells resulted in greater targeted mutagenesis efficiency up to 600% without noticeable increase in off-target effects. Improved activity of the two fusion proteins should enable editing of the previously hard-to-edit genes and thus readily obtaining the cells with designer traits.

## Introduction

Clustered regularly interspaced short palindromic repeats (CRISPR) and CRISPR-associated 9 (Cas9) constitute an adaptive immune system found in bacteria, archaebacteria, and bacteriophages^1–3^, but thanks to its programmability and ease of use, CRISPR-Cas9 has been repurposed for genome editing in various applications, including research, therapeutics, microbiome manipulation, and agriculture^4–8^.

Within the CRISPR-Cas9 system, the functional RNA-Cas9 complex consists of a Cas9 effector protein that acts as a DNA cleaving enzyme and two single strand guide RNAs, namely, a trans-acting CRISPR RNA (tracrRNA) and a CRISPR RNA (crRNA), which guide the complex to the target sequence. The RNA-Cas9 ribonucleoprotein (RNP) complex localizes to its target sequence in two steps; it first locates and binds to the protospacer adjacent motif (PAM) (5′-NGG-3′ for Cas9) of the nontarget strand and then unwinds the target DNA to complementarily bind between crRNA and the target strand of the protospacer sequence in the genome. In eukaryotic cells, the dsDNA breaks (DSB) created in the target DNA by Cas9 RNP are repaired by either homology-directed repair (HDR) or nonhomologous end joining (NHEJ)^9^. Error-prone NHEJ process can produce insertion and/or deletion (indel) mutations of variable length at the target site, whereas HDR-mediated repair can replace the DNA sequence with the help of a single-stranded or double-stranded DNA donor template.

The Cas9 protein from *Streptococcus pyogenes* (SpyCas9) has been extensively characterized and developed for various applications since initial demonstration in 2012^1,10^. Various steps in the mode of action of the SpyCas9 RNP have been targeted for improvement, including protein engineering of the domains interacting with PAM, nontarget strands, and crRNA, which enabled specificity to be improved^11^; furthermore, chemical modifications to the guide RNA for stability have improved SpyCas9 function^12^. However, efforts to enhance efficiency are relatively unattended. Enzyme kinetics is affected by both how fast the enzyme (E) binds to the substrate (S) to form ES complex that leads to an enzyme-product (EP) complex, and also by how fast the enzyme dissociates from the EP complex and hits another substrate^11^. Different from conventional enzymes, the energetically stable complex between the SpyCas9 RNP and protospacer DNA makes the ternary RNA–protein-DNA structure difficult to dissociate, and this property makes Cas9 a single-turnover enzyme that does not follow the conventional Michaelis and Menten kinetics^13^.

In an attempt to enhance the enzymatic activity of SpyCas9, various proteins including endonucleases, exonucleases, ligase inhibitors, deaminases, and an RNA reverse transcriptase have been translationally fused to the SpyCas9 protein. When an *E*. *coli* 3′ to 5′ Exonuclease I (sbcB) was fused to SpyCas9, the chimeric protein generated the longer deletions of DNA in Zebrafish relative to SpyCas9 alone^14^. In addition, a SpyCas9 chimeric fusion protein with the three prime repair exonuclease 2 (TREX2), a human 3′ to 5′ exonuclease involved in DNA repair, replication, and recombination, was also reported to increase mutagenic efficiency^15,16^. TEXT (Tethering EXonuclease T5 with FnCas12a)—a fusion strategy significantly increased the knockout efficiency of FnCas12a at multiple genomic loci in different human cell lines^17^. In case of the knock-in activity, when the human CtIP endonuclease was fused to SpyCas9, the chimeric protein resulted in more than two-fold increase in transgene integration efficiency relative to SpyCas9 backbone protein alone^18^. A dominant-negative mutant of 53BP1, DN1S, was fused to Cas9, and the Cas9-DN1S fusion proteins significantly blocked NHEJ events specifically at Cas9 cut sites and improved HDR frequency^19^. Rad52-Cas9 fusion strategies yielded approximately threefold increase in HDR during the surrogate reporter assays in human HEK293T cells^20^. Furthermore, the chimeric fusion of adenine or cytidine deaminases to nuclease-inactivated version of SpyCas9 resulted in base editing without DSB, concomitantly reducing unintended off-target effects^21,22^. Lastly, an engineered reverse transcriptase fused to a catalytically-impaired SpyCas9 and a prime editing-guide RNA (pegRNA) led to specific change of the base using the pegRNA as a repair emplate^23^.

Because the chimeric fusion proteins with the SpyCas9 were shown to widen the spectrum of capability of the site-specific nuclease SpyCas9.We attempted to test the accessary proteins that may weaken the binding energy of the chimeric proteins to the substrate DNA and thus increase a turnover rate to eventually enhance the genome editing efficiency. To this end, we first created fusion proteins in which the carboxy-terminus of SpyCas9 was linked with a DNA-modifying protein—either a 5′ to 3′ DNA exonuclease (RecE, RecJ, T5, or lambda)^24^, mung bean nuclease, or terminal deoxynucleotidyl transferase (TdT). T5 and RecJ exonucleases were reported to enhance FnCas12a, T5-FnCas12a increased the knockout efficiency of FnCas12a at multiple genomic loci in different human cell lines, while RecJ-FnCas12a decreased the knockout efficiency of FnCas12a^17^.

The green fluorescent protein (GFP) was also tested as a control. Of the seven tested, SpyCas9 fusion with RecJ (SpyCas9-RecJ, C9R) and surprisingly with GFP (SpyCas9-GFP, C9G) resulted in a marked increase in both mutagenesis and knock-in efficiency, while the off-target effects are not significantly increased relative to the SpyCas9 (C9) structure alone. We refer the two functionally enhanced fusion proteins of C9R and C9G to as CRISPR PLUS, and explore how efficiently they edit the DNA at different conditions such as in test tube, cultured cell line, primary human cells, and plant protoplasts.

## Results and discussion

### Increased genome-editing efficiency of Cas9 chimeric fusion with RecJ or GFP

**Figure 1:**
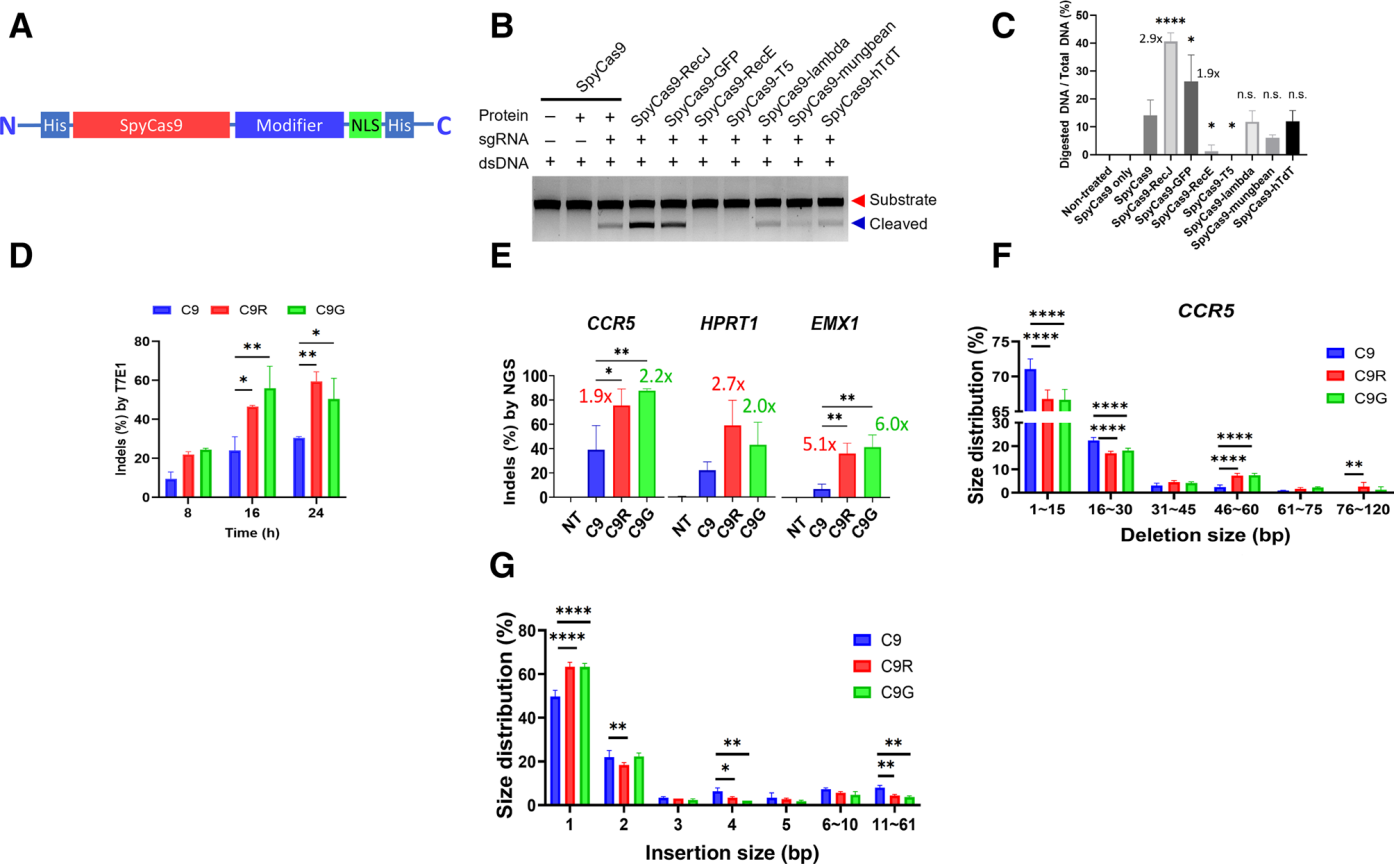
Marked increase in indel efficiency of the Cas9-RecJ and Cas9-GFP chimeric fusion proteins. (**A**) Schematic of a translational fusion of a modifier protein to SpyCas9. His tag (6X His) for protein purification and nuclear localization signal (NLS) were placed. (**B**) Comparison of the DNA cleavage activity of SpyCas9 and its chimeric proteins, C-terminal chimeric SpyCas9 translational fusion proteins with the nucleases RecJ, RecE, T5, lambda, mung bean exonuclease, and human terminal deoxynucleotidyl transferase (TdT), and with green fluorescent protein (GFP). The modifier proteins are described in Supplementary Table 1. The *CCR5* DNA was digested with the purified enzymes and resolved on an agarose gel. Successful digestion cuts the 1.5 kb substrate DNA into two 0.75 kb DNAs. (**C**) DNA band intensities in the gel image. Statistical comparison was made between C9 and each of the C9R or C9G proteins. Significance was indicated on top of each bar. One-way ANOVA using Tukey’s multiple-comparison test. The fold change relative to C9 is indicated above each bar. (**D**) DNA insertion and deletion (Indel) efficiency of C9, C9R, and C9G for the *CCR5* gene in HEK293T cells at 8, 16, and 24 h after RNP transfection as measured by a T7E1 assay. Averages from the three replicate experiments are plotted. Two-way ANOVA using Tukey’s multiple-comparison test. (**E**) Indel efficiency of C9, C9R, and C9G for the *CCR5*, *HPRT1*, and *EMX1* genes (left to right) in HEK293T cells. At 24 h (*CCR5* and *HPRT1*) and 8 h (*EMX1*) post-transfection, cells were harvested and subjected to targeted deep sequencing. The experiments were performed in triplicate. NT group represent the NGS data from non-treated control. One-way ANOVA using Tukey’s multiple-comparison test was performed using C9 data as control. (**F**) Distribution of the DNA deletion sizes after treatment with C9, C9R, and C9G. The number of deletion events around the protospacer site in the CCR5 gene was counted according to the 15 bp deletion intervals from targeted deep sequencing data (n = 3) data. (**G**) Distribution of DNA insertion sizes after treatment with C9, C9R, and C9G in the CCR5 gene. The number of insertion events was counted according to the 1 bp insertion intervals from targeted deep sequencing data (n = 3) data. 6–10 and 11–61 bp insertion intervals were also shown to include events with smaller numbers. ****P < 0.0001, ***P < 0.001, **P < 0.01, *P < 0.05, and n.s. indicates not significant.

To prepare Cas9 chimeric fusion proteins, we translationally fused the carboxy terminus of SpyCas9 to a modifier protein: 5′-to-3′ DNA exonucleases (including RecE, RecJ, T5, and lambda), mung bean nuclease, TdT, or GFP (Fig. 1A) (S1 Text). The SpyCas9 chimeric fusion proteins, were expressed in bacterial cells and purified. The SDS-PAGE pattern shows that all chimeras were isolated as single proteins (S1 Fig), indicating successful fusion of the accessory proteins and purity to perform functional analysis. The SpyCas9-T5 protein was barely detectable (< 0.2 mg/L) due to its low expression level.

To investigate the activity of the seven fusion proteins, we performed an in vitro DNA cleavage assay with the therapeutically relevant loci examined by other colleagues for cross comparison^25^. The 1.5 kb PCR-amplified *C–C chemokine receptor type 5* (*CCR5*) DNA was cut in vitro by C9 and the CRISPR PLUS proteins, 750 bp DNA fragments were produced (Fig. 1B). A 0.25 pmol of each SpyCas9 fusion protein and a 0.3 pmol of sgRNA^CCR5^ were mixed and incubated at room temperature for 15 min. A total of 250 ng of DNA was digested with preassembled RNP at 37 °C for 10 min. The enzymatic products were then quantified from the agarose gel using ImageJ and the corresponding enzymatic activities were calculated. C9 exhibited 14.1% cleavage activity, whereas C9R and C9G showed 40.6% and 26.3% activity, respectively (Fig. 1C). By contrast, fusion with RecE resulted in difficulty purifying the proteins possibly due to instability of C9-RecE chimeric fusion structure, to 1.3%, and fusion with lambda, mung bean nuclease, and hTdT did not significantly affect the C9 activity. Together, these results demonstrate that the fused fragments affect the overall enzymatic activities of the Cas9. Henceforth, we further analyzed only C9R and C9G due to their desired effects on activity.

### Improved indel efficiency of CRISPR PLUS in the cells

To check whether C9R induces DNA insertion and deletion (Indel) mutations more rapidly than does C9, we first confirmed that the RecJ moiety of C9R is functional in vitro and fluorescence activity of GFP domain of C9G (S2 Fig.). We then introduced C9, C9R, and C9G ribonucleoproteins (RNPs) targeting the *CCR5* gene into HEK293T cells and stopped the incubation at 8, 16, or 24 h after transfection; cells were then harvested and subjected to a T7 endonuclease 1 assay, the DNA was resolved on a 2% gel, and the results of the three replicates were digitized using ImageJ. C9R and C9G reached 20% efficiency as early as 8 h, a level that was not achieved until at least 16 h for the C9 control (Fig. 1D). Relative to C9, CRISPR PLUS displayed approximately twofold increase in efficiency at 16 h.

To analyze the enzymatic activity in relation to different gene targets, we transfected the preassembled RNPs of C9, C9R, and C9G targeting CCR5 into HEK293T cells and incubated them for 24 h; the indel efficiency was 39%, 76%, and 87%, respectively (Fig. 1E). Meanwhile, C9, C9R, and C9G displayed efficiencies of 22%, 59%, and 43%, respectively, for *hypoxanthine phosphoribosyltransferase 1 (HPRT1*) and 7%, 36%, and 42% for *Empty Spiracles Homeobox 1 (EMX1*; Fig. 1E). Overall, C9R displayed approximately two- to fivefold increased efficiency; C9G also exhibited two- to sixfold increased activity relative to C9.

Because the RecJ moiety of C9R as a 5′ to 3′ ssDNA exonuclease resects DNA from 5′ to 3′ direction, the deletion created by C9R at DSB could be longer that that by C9. To determine whether the increased indel efficiencies of the CRISPR PLUS proteins are associated with deletion size, we first analyzed the correlation between deletion size and the number of NGS-based deep sequencing reads (> 40,000 per locus) at the *CCR5* target locus. Compared to C9, CRISPR PLUS exhibited significantly reduced percentages of small deletions of 1–15 and 16–30 bp (Fig. 1F), but significant increases in longer DNA deletions of 46–60 and 76–120 bp. Conversely, insertions tended to be shorter than those using C9 (Fig. 1G).

Next, we evaluated the off-target effects of CRISPR PLUS. Five potential off-target sequences of the *CCR5*, *HPRT1*, and *EMX1* genes were identified by Cas-OFFinder^7^ (S3 Fig). For sequences with > 50,000 NGS reads, the error rates of CRISPR PLUS did not exceed that of conventional NGS sequencing error rate (0.01–0.1%) (S3 Fig), suggesting that the high on-target editing efficiency of CRISPR PLUS is accompanied by low off-target effects.

Taken together, we demonstrated that CRISPR PLUS consistently displayed greater efficiency in indel mutagenesis in three different genes of HEK293T cells, whereas the off-target effects are not increased as compared with C9 treatment. Now we turn to testing knock-in efficiency of the CRISPR PLUS proteins.

### Enhanced homology-directed DNA repair (HDR) of CRISPR PLUS

**Figure 2:**
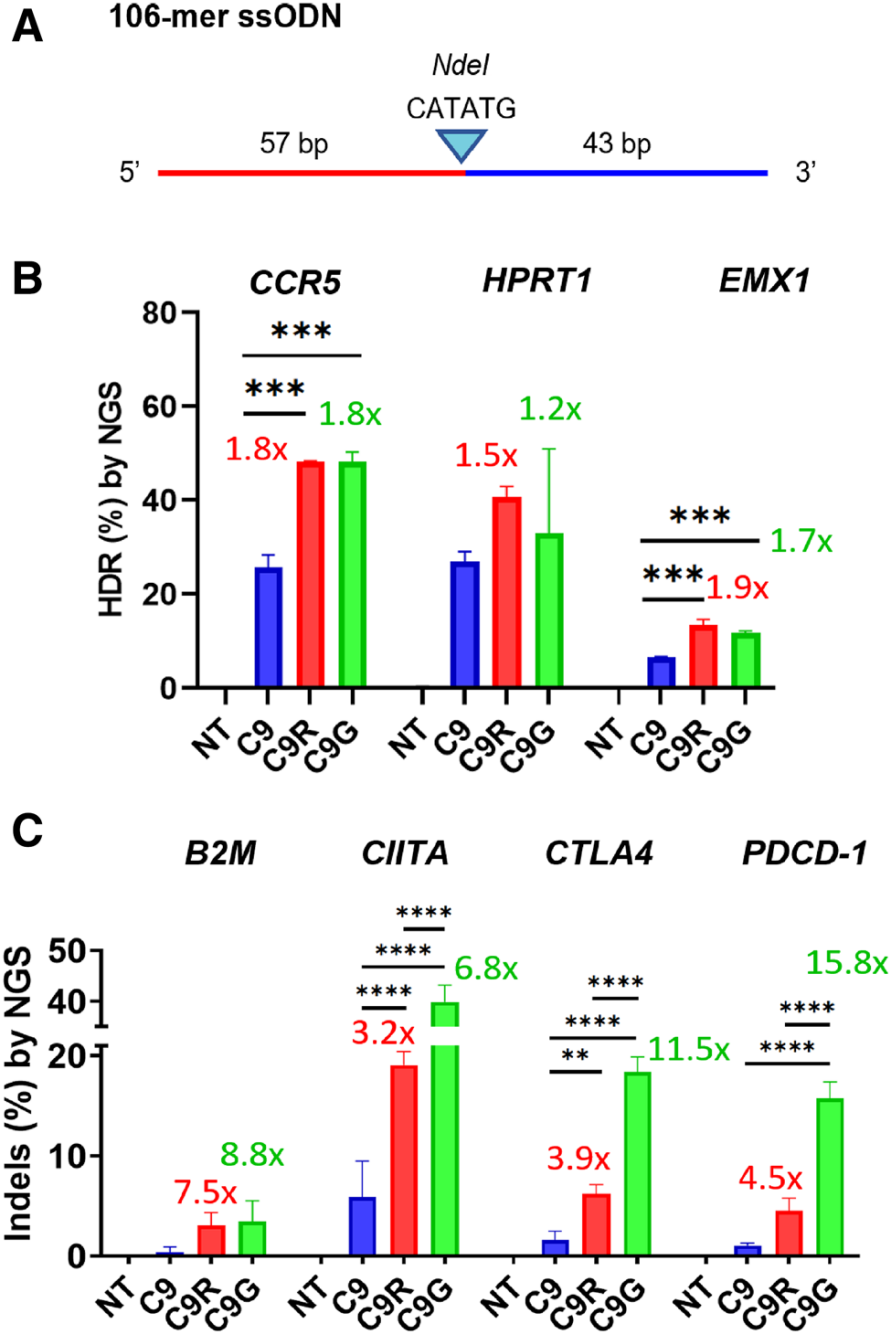
Increase in knock-in efficiency in HEK293T cells and enhanced multiplex indel efficiency of chimeric proteins in iPSCs. (**A**) Schematic of a 106-mer single-stranded oligonucleotide (ssODN). Artificially introduced *NdeI* restriction site (blue triangle) was flanked with homologous 5′ arm (57-mer) and 3′ arm (43-mer). (**B**) Comparison of homology-directed DNA repair (HDR) activity among C9, C9R, and C9G. HDR efficiency was evaluated by targeted deep sequencing of the *CCR5*, *HPRT1*, and *EMX1* loci (left to right) after genome editing with the specific 106-mer template shown in panel a. (****P < 0.0001 and ***P < 0.001, one-way ANOVA using Tukey’s multiple-comparison test). For each gene, fold change was calculated relative to C9 efficiency. (**C**) Four different RNPs, each preassembled with a different sgRNA (targeting *B2M*, *CIITA*, *CTLA4*, or *PDCD-1*) were simultaneously transfected into induced pluripotent stem cells (iPSCs) and incubated for 24 h, and the targeted deep-sequencing analysis was performed to determine the indel efficiencies (****P < 0.0001, ***P < 0.001, and **P < 0.01, two-way ANOVA using Tukey’s multiple-comparison test).

In addition to indel mutagenesis, knock-in replacement of the sequence is the major area of CRISPR-Cas9 applications. We therefore investigated whether CRISPR PLUS improves HDR activity for the three target genes in HEK293T cells. The cell cycle was first synchronized using nocodazole^26^ before transfection with both the preassembled RNP complex (protein:sgRNA = 30:60 pmol) and a 106-mer single strand oligodeoxynucleotide (ssODN) repair template (20 pmol) consisting of a 57-nt 5′ homology arm, 5′-CATATG-3′ *Nde*I restriction enzyme site, and 43-nt 3′ homology arm (Fig. 2A). Each target site was amplified and analyzed using targeted amplicon sequencing, and the HDR efficiencies were evaluated by *Nde*I digestion of the amplified DNA (S4 Fig). C9 exhibited 20%, 23%, and 5% efficiency for the *CCR5*, *HPRT1*, and *EMX1* sites, respectively; these values were 36%, 48%, and 12% for C9R and 36%, 39%, and 11% for C9G (S4 Fig). Therefore, relative to C9, CRISPR PLUS increased the knock-in replacement efficiency by 6–16%. When the efficiency was re-examined using targeted deep sequencing analysis, the efficiency of C9 for the *CCR5*, *HPRT1*, and *EMX1* sites was found to be 26%, 27%, and 6%, respectively; that of C9R was 48%, 41%, and 13%; and that of C9G was 48%, 33%, and 12%, confirming that CRISPR PLUS increased the knock-in efficiency by 6–22% (Fig. 2B). It is likely that increased knock-in efficiency might be associated with the increased editing activity of CRISPR PLUS enzymes.

### Increased multiplex genome editing efficiency in induced pluripotent stem cells (iPSCs) and plant protoplasts

Finally, to determine whether the enhanced genome editing efficiency might be applicable to induced pluripotent stem cells (iPSCs), a cell type that is recalcitrant to editing, we performed multiplex genome editing of the *beta 2 microglobulin* (*B2M*), *class II major histocompatibility complex transactivator* (*CIITA*), *cytotoxic T‐lymphocyte-associated antigen 4* (*CTLA4*), and *programmed cell death protein 1* (*PDCD1*). When it comes to regenerative medicine using universal iPSCs, deletion of *B2M* and *CIITA* in iPSCs suppresses expression of their human leucocyte antigen (HLA) Class I and Class II genes, respectively, such that the genome edited iPSCs can avoid host T cell-mediated clearance^27^. *CTLA4* and *PDCD1* are inhibitory receptors that take part in T cell exhaustion, therefore being the target of deletion when designing engineered T cells for cancer immunotherapy^28^. We first designed two sgRNAs for each of the four genes, tested in HEK293T cells, and selected the more efficient sgRNA from each pair, where sgRNA^B2M^-2, sgRNA^CIITA^-1, sgRNA^PDCD1^-1, and sgRNA^CTLA4^-1 were chosen for the iPSC experiments (S5 Fig).

After in vitro assembly of each of the four sgRNAs with effector proteins, namely, C9, C9R, and C9G, three sets of four RNPs were transfected into HEK293T before moving to iPSCs. At three days post-electroporation, T7 endonuclease I assay (T7E1) was performed. Three-day treatment resulted in indel efficiency 38–81% regardless of C9, C9R, and C9G (S6 Fig). In iPSCs, compared with C9, CRISPR PLUS increased indel efficiency 7.5-fold and 8.8-fold for *B2M* (Fig. 2C), 3.2- and 6.8-fold for *CIITA*, 3.9- and 11.5-fold for *CTLA4*, and 4.5- and 15.8-fold for *PDCD-1*, respectively, suggesting possibility to obtain multiplex homozygous mutations in the target genes within shorter time than using C9.

Plant protoplasts are an example of difficult-to-edit cells partly because the nucleofection of the RNP complex damages the cells. Therefore, obtaining desired level of editing efficiency with a tolerable amount of RNP is a demanding technique. To determine whether C9R activity is reproducible in plants, C9 and C9R RNPs programmed to target α-1,3-fucosyltransferase 1 were transfected into protoplasts of the model plant *Nicotiana benthamiana* by the DNA-free genome editing method (as described in previous studies^29^), and their editing efficiency were analyzed by NGS deep sequencing. Compared to C9, C9R had increased editing efficiency by 2.6-fold, (S7 Fig), suggesting that C9R activity is conserved regardless of the plant or animal system.

Taken together, we have shown that the two CRISPR PLUS proteins display markedly increased efficiency in both indel mutagenesis and knock-in efficiency at the various conditions tested such as in vitro DNA cleavage, in plant protoplasts, human cells, and in iPSC cells. However, currently, we do not have a complete explanation about the mechanisms of action of the enhanced genome editing efficiency observed from CRISPR PLUS especially in the C9G protein. In fact, it was not too much surprising that the C9R showed greater efficiency relative to C9 alone, because RecJ moiety could contribute to leaving permanent mutation footprint at the site of DSB. However, it was indeed unexpected results obtained from C9G experiments.

To rule out any possibility of experimental errors, we examined the identity of the C9G protein and performed the editing experiments repeatedly with separately prepared batches of C9G, but obtained basically the same results. First, we double checked if our C9G protein surely has GFP moiety by examining the photochemical spectra of both C9 and C9G. Specific light absorption and emission of C9G peaked around 400 and 500 nm, respectively as expected, whereas C9 did not noticeably absorb or emit at these wavelengths (S2 Fig. B). Emission of green light from C9G protein confirms that we used the right proteins. In addition, it can be postulated that the GFP moiety might contribute to stabilizing the C9G protein, and the relatively stable C9G acts longer time than C9 to result in overall increase in genome editing efficiency. However, our data shows that the activity was found enhanced at as early as 16 h, and not significantly increased then after (Fig. 1D). On the other hand, it is also of note that the nucleotide deletion size of both C9R and C9G similarly decreased at smaller size (1–30 nt) but increased at larger section (46–60 nt) (Fig. 1F). Thus, the mode of action of C9G could be similar to C9R, but it needs further research to draw definitive conclusion about the enhanced genome editing activity of the CRISPR PLUS proteins.

One of the remaining hypotheses to explore through future research is that chimerically fused GFP might have transformed the Cas9 moiety to conformationally favorable ways, and this resulting C9G protein has increased the turnover rate. Conceptually opposite to the inhibitory activity of anti-CRISPR proteins including AcrIIA2 and AcrIIA4^30^, the fused GFP moiety might interact with Cas9 part so that the C9G proteins better interrogate PAM and target site or release from the target DNA after creation of DSB. Previously, it was shown that, depending on the linker structure, GFP moiety in the chimeric fusion with an acid phosphatase (Pho-C) could differentially affect the enzyme activity^31^. Lastly, understanding the crystal structure of C9 in complex with RecJ or GFP should give us better answer concerning how the chimeric proteins performs better in creation of DNA DSBs.

## Conclusion

In conclusion, we have shown that CRISPR PLUS greatly enhanced genome editing efficiency while keeping the off-target effect low relative to C9 control. Three genes in human HEK293T cells, four in iPSCs, and one in plant cells were tested, and consistently showed that CRISPR PLUS exceeded the editing efficiency of C9. Given that the off-target effects are proportional to duration and concentration of the genome editing enzymes in the cells of treatment^11^, CRISPR PLUS would further decrease the possibility of off-target effects because CRISPR PLUS achieved a genome editing rate within shorter time with relatively lower concentration than those of C9. Greater efficiency is also useful when practicing multiplex genome editing because the possibility to have homozygous alleles simultaneously at the multiple loci equals to the product of the editing rate at an individual locus. Our results should represent breakthrough techniques for multiplex genome editing of the precious and hard-to-edit cells for therapeutics and expedite availability of the regenerative medicines closer.

## Methods

### Cloning of SpyCas9 fusion proteins

The SpyCas9 coding sequence (Addgene, #138566) was ligated into a pET28a vector (Merck Biosciences, Darmstadt, Germany) that had been linearized by *Nco*I and *Xho*I double digestion. The pET28a-SpyCas9 vector, which was named pNGP020, was again linearized by *Xho*I which was 9 bp downstream of the full-length SpyCas9 sequence. A BPNLS signal peptide and DNA-modifying proteins were introduced to the *Xho*I site in pNGP020 by digestion of the *Xho*I site; pNGP020-BPNLS was named pNGP021. Full-length RecJ exonuclease amplified from *E*. *coli* DH5α was cloned into the pNGP020 vector by Gibson assembly (NEB, Ipswich, MA), which was then named pNGP022. With the same manner, the GFP gene was amplified from modified GFP4^32^, and the SpyCas9-GFP-BPNLS was named pNGP056. RecE exonuclease was amplified from *E*. *coli* DH5α, and the SpyCas9-RecE-BPNLS was named pNGP024. T5 phage exonuclease was synthesized by GenScript (Piscataway, NJ, USA), and the SpyCas9-T5-BPNLS was named pNGP041. Lambda phage exonuclease was synthesized by IDT (Coralville, IA, USA), and the SpyCas9-Lambda-BPNLS was named pNGP097. Mung bean exonuclease was amplified from cDNA of mung bean roots purchased from a local grocery store, and the SpyCas9-mungbean exonuclease-BPNLS was named pNGP030. Full-length human TdT (NM_004088) was obtained from the GenScript cDNA library (OHu11174D), and the SpyCas9-hTdT-BPNLS was named pNGP072. The DNA sequences mentioned here are provided in Supplementary File 1.

### Protein expression and purification

All the vectors encoding SpyCas9 fusion proteins are based on the plasmid vector pET28a backbone and are composed of the SpyCas9, fusion protein, a nucleoplasmin nuclear localization signal (NLS) and 6X-His tag sequences at the N- or C-termini. For bacterial expression of the SpyCas9 chimeric fusion proteins, the plasmids were transformed into Rosetta2 (DE3) pLysS cells. On the first day, *E. coli* cells harboring the SpyCas9 expression vector from glycerol stocks were inoculated into LB medium containing 50 mg/L kanamycin and cultured at 37 °C overnight. On the following day, the growing *E. coli* cells were reinoculated into fresh LB medium and incubated at 37 °C until the optical density at 600 nm (OD_600_) reached 0.4–0.7, indicating the exponential growth phase. Expression of the SpyCas9 fusion proteins was induced by the addition of isopropyl-β-D-thiogalactopyranoside (IPTG; final concentration 1 mM) for 20 h at 18 °C. Each bacterial culture was centrifuged at 3700*g* for 30 min, and then the pellet was resuspended in lysis buffer (20 mM Tris–HCl pH 8.0, 500 mM NaCl, 5 mM imidazole, 1 mM 1,4-dithiothreitol (DTT), and 1 mM phenylmethylsulfonylfluoride (PMSF)). The resuspended cells were lysed by sonication with a period of 2 s on and 6 s off at 40% amplitude for 40 min and then centrifuged at 15,000*g* for 1 h. The supernatant was passed through a 0.45 μm syringe filter and injected into an AKTA FPLC system (GE Healthcare, Chicago, IL, USA) for purification of the SpyCas9 fusion proteins. Immobilized metal affinity chromatography (IMAC, HisTrap HP 5 mL; GE Healthcare) was performed after washing and equilibrating the AKTA FPLC system. The affinity columns were also washed with ethanol (followed by water) and binding buffer (20 mM Tris–HCl pH 8.0, 500 mM NaCl, 5 mM imidazole). The protein was eluted in elution buffer (20 mM Tris–HCl pH 8.0, 500 mM NaCl, 500 mM imidazole) in a step gradient from 14 to 100%. The flow-through was injected into a desalting column (HiPrep 26/10 Desalting; GE Healthcare) equilibrated with the desalting buffer (20 mM HEPES, 150 mM KCl, 1 mM DTT, 10% (v/v) glycerol, pH 7.5) to replace the imidazole-containing IMAC elution buffer with a final storage buffer to ensure the long-term stability of the eluted proteins. An aliquot of the eluate was subjected to 10% SDS-PAGE to evaluate protein purity. Finally, the proteins were concentrated in a Vivaspin Turbo 15 concentrator (50,000 MWCO; Sartorius, Germany). The concentrated proteins were quantified with the conventional Bradford assay.

### In vitro sgRNA transcription

To prepare sgRNAs for in tube cleavage assay, a double-stranded DNA template was generated by annealing two single-stranded oligonucleotides with complementary sequences (forward: 5′-AATTTAATACGACTCACTATAGGXXXXXXXXXXXXXXXXXXXXGTTTTAGAGCTAGAAATAGCAAGTTAAAATAAGGCTAGTCCGTTATCAACTTGAAAAAGTGGCACCGAGTCGGTGCTTTT-3′, reverse: 5′-AAAAGCACCGACTCGGTGCCACTTTTTCAAGTTGATAACGGACTAGCCTTATTTTAACTTGCTATTTCTAGCTCTAAAACXXXXXXXXXXXXXXXXXXXXCCTATAGTGAGTCGTATTAAATT-3′) at 95 °C for 5 min and 55 °C for 10 min. ‘X’s can be replaced with the spacer sequences for each sgRNA. The DNA was purified using QIAquick (Qiagen) columns, and the purified DNAs were then used as templates for a T7 in vitro transcription (IVT) reaction according to manufacturer’s direction (MEGAshortscript T7, Invitrogen, Carlsbad, CA, USA). In vitro transcribed sgRNAs were DNase treated, precipitated using the ammonium acetate/ethanol method, and then resuspended in distilled water for use with the SpyCas9 and its chimeric fusion proteins.

### In tube cleavage assay of the SpyCas9 fusion proteins

The *CCR5* locus from HEK293T cells was amplified by PCR (Supplementary Table 5) with Q5 High-Fidelity DNA Polymerase (New England Biolabs, Ipswich, MA, USA). For RNP complex formation, 0.25 pmol of each SpyCas9 fusion protein and 0.3 pmol of sgRNA^CCR5^ were mixed and incubated at room temperature for 15 min. A total of 250 ng of DNA was digested with preassembled RNP at 37 °C for 10 min. Digested DNA was placed on ice, and the products were subjected to 2% agarose gel electrophoresis. Assessment of the digested DNA intensity was performed by ImageJ^33^. The digested fraction was calculated with the following formula: Digested fraction (%) = *b*/(*a* + *b*) × 100, where *a* represents the band intensity of the DNA substrate and *b* represents the digested DNA.

### Cell culture and synchronization

HEK293T cells were cultured in Dulbecco’s modified Eagle’s medium (DMEM; HyClone) supplemented with 10% fetal bovine serum and cultured at 37 °C in a humidified 5% CO_2_ incubator. HEK293T cells were seeded at 3 × 10^6^ cell density in a 10-cm culture dish. To help improve the efficiency of the knock-in (described below), the cell cycles were synchronized by treatment with nocodazole (200 ng/ml) for 17 h before electroporation.

### RNP preparation and nucleofection for knock-in experiments

Before transfection of proteins into the HEK293T cells, purified SpyCas9, C9R, and C9G (30 pmol) and synthetic sgRNA (60 pmol; Thermo Fisher Scientific, Waltham, MA, USA) were incubated at room temperature for 20 min for RNP assembly (sgRNA information, Supplementary Table 4). In the case of the knock-in experiment, 20 pmol ssODN with phosphorothioate bonds in the first and last two nucleotides was then added with the RNP complex. The sequences of the single-stranded oligodeoxynucleotides (ssODNs) used as donor templates for the *CCR5*, *HPRT1*, and *EMX1* loci are listed in Supplementary Table 3. Each nucleofection reaction consisted of 2 × 10^5^ HEK293T cells in 20 μl of nucleofection reagent mixed with 10 μl of RNP:DNA. For multiplex experiments, 25 pmol of SpyCas9, C9R, or C9G protein and 50 pmol of modified sgRNAs for *B2M*, *CIITA*, *PD1*, or *CTLA4* (Supplementary Table 7) (Synthego, Menlo Park, CA, USA) were mixed separately at room temperature for 20 min before transfection. Nucleofection of HEK293T cells was performed using 4D-Nucleofector (Lonza) with the CM-130 program.

### T7 endonuclease I assay

After harvesting samples at 24 h posttransfection, genomic DNA was extracted using a PureLink Genomic DNA Kit (Thermo Fisher Scientific, Waltham, MA, USA) following the manufacturer’s protocol. Primers for the *CCR5*, *HPRT1*, and *EMX1* loci are listed in Supplementary Table 5. Purified PCR products (250 ng) in NEBuffer 2 (New England Biolabs, Ipswich, MA, USA) were denatured (95 °C for 10 min), re-annealed by gradually cooling from 95 to 85 °C at − 2 °C/s and 85 to 25 °C at − 0.1 °C/s and then held at 4 °C using a programmable thermocycler. The PCR products were treated with 10 U of T7 endonuclease 1 (T7E1; New England Biolabs) in a 20 μl final reaction at 37 °C for 35 min. The product was separated on a 2% agarose gel. The band intensity was analyzed using ImageJ. The calculation was conducted by the following equation: % mutation = 100 × (1 − (1 − *f*)^1/2^), where ‘*f*’ is the fraction cleaved.

### HDR analysis by *Nde*I restriction digestion

Aa artificially inserted sequence of the *Nde*I restriction site was PCR amplified (Supplementary Table 5), and the PCR products (1 µg) were digested with 20 U of *Nde*I (New England Biolabs, Ipswich, MA, USA) at 37 °C for 3 h. The digested DNA was quenched with 6X gel loading dye (Purple, New England Biolabs), at 70 °C for 10 min and electrophoresed on a 2% agarose TAE gel. Digested DNA fragment intensity was analyzed and evaluated with ImageJ software.

### Targeted deep sequencing of on- and off-target sites

Five potential off-target sites for each gene were predicted using Cas-OFFinder^7^. The genomic region flanking the target site for each gene was amplified by a two-step PCR method. First, genomic DNA from the edited and control samples was isolated and PCR-amplified for 35 cycles using Q5 High-fidelity DNA polymerase with adapter primers. The resulting amplicons were purified using a QIAquick PCR Purification Kit (Qiagen, Venlo, The Netherlands). The samples were subjected to eight cycles of PCR using KAPA HiFi HotStart DNA Polymerase (Roche, Basel, Switzerland) for indexing with MiniSeq High Output Kit (Illumina, FC-420-100X), followed by purification using a QIAquick PCR Purification Kit (Qiagen). Purified DNA samples were quantified with a Qubit 2.0 fluorometer and pooled in an equimolar ratio. Pooled libraries were sequenced in 150 bp read-length with the Illumina MiniSeq (Illumina, SY-420-1001). The PCR primer sequences used to analyze the off-target effects with NGS are listed in Supplementary Table 6.

### Validation of C9R exonuclease activity under in vitro conditions

A 60 nM aliquot of either SpyCas9 or C9R was added to the target template, either 125 ng of dsDNA (900 bp) or 3.5 μg of ssDNA (123 bp), with or without Mg^2+^ ions in a 20 μl reaction volume. NEBuffer 3.1 (New England Biolabs, Ipswich, MA, USA) was used as the reaction buffer. After 1.5 h of incubation at 37 °C, the reaction mixtures were assessed by electrophoresis on a 2% agarose gel.

### Plant growth conditions and protoplast transfection of *N. benthamiana*

All plants were grown under 150 μmol m^−2^ s^−1^ LED light under long-day (14-h light/10-h dark photoperiod) conditions at 25 °C. Tobacco (*Nicotiana benthamiana*) seeds were sterilized in a 0.4% hypochlorite solution for 1 min, washed three times in distilled water, and sown on 0.5 × Gamborg B5 solid medium supplemented with 2% sucrose. Four-week-old leaves grown in B5 media were treated with enzymes to digest the cell wall (1.5% cellulose R10, 0.3% macerozyme R10, 0.5 M mannitol, 8 mM CaCl_2_, 5 mM MES [pH 5.7], 0.1% BSA) for 4 h at 25 °C in darkness. The mixture was filtered through a 100 µm cell strainer (Thermo Fisher Scientific, Waltham, MA, USA), and then flow-through protoplasts were collected by centrifugation at 100*g* in a round-bottom tube for 6 min. Protoplasts were resuspended and washed with W5 solution (154 mM NaCl, 125 mM CaCl_2_∙2H_2_O, 5 mM KCl, 2 mM MES pH 5.7) and pelleted by centrifugation at 100*g* for 6 min. Finally, protoplasts were resuspended in MMG solution (0.4 M mannitol, 15 mM MgCl_2_, 4 mM MES pH 5.7) and counted under a microscope using a hemocytometer. Protoplasts were diluted to a density of 1 × 10^6^ protoplasts/ml of MMG solution and stabilized for at least 30 min at 4 °C before PEG-mediated transfection.

Protoplasts (2 × 10^5^) were transfected with SpyCas9 and C9R proteins (10 µg) premixed with in vitro-transcribed sgRNA (20 µg) targeting α-1,3-fucosyltransferase 1 (FucT 13-1) (Supplementary Table 4). Prior to transfection, SpyCas9 protein was mixed with sgRNA in 1 × NEB buffer 3 and incubated for 10 min at room temperature. A mixture of protoplasts was resuspended in 200 µl MMG solution and gently mixed with 10–20 µl of RNP complex and 210–220 µl of freshly prepared PEG (0.2 M mannitol, 40% w/v PEG-4000, 100 mM CaCl_2_) solution and then incubated at 25 °C for 15 min. After a 15 min incubation at room temperature, transformation was stopped by adding 840–880 μl of W5 solution. Protoplasts were then collected by centrifugation for 2 min at 100*g* at room temperature, washed once with 1 ml of wash buffer, and then collected by centrifuging for another 2 min at 100*g*. The density was adjusted to 1 × 10^5^ protoplasts/ml, and they were cultured in modified Protoplast Induction Medium (PIM) medium (1.58 g B5 medium, 103 g sucrose, 0.2 mg 2,4-D, 0.3 mg 6-Benzylaminopurine (BAP), 0.1 g MES, 375 mg CaCl_2_∙2H_2_O, 18.35 mg NaFe-EDTA and 270 mg sodium succinate).

### iPSC reprogramming and maintenance

Human dermal fibroblasts (DFs; ATCC, Manassas, VA, US) were plated in 24-well plates. After one day, the cells were transduced with reprogramming factors (Oct4, Sox2, Klf-4, and c-Myc) in 5 μg/mL polybrene (Sigma, St. Louis, MO, US) based on a multiplicity of infection 10. After 5 days, transduced cells were transferred to STO feeder cells (ATCC) in feeder medium [DMEM/F12 (Thermo Fisher Scientific, Waltham, MA, US) supplemented with 10% FBS (Thermo) and 1 × primocin (InvivoGen, San Diego, CA, US)]. After 2 days, medium exchange was initiated with iPSC medium [DMEM/F12 supplemented with 20% serum replacement (Thermo), 1 × primocin and 4 ng/mL bFGF (Thermo)]. Newly-formed colonies were consistently subcultured by mechanical picking under a stereoscopic microscope. Picked colonies were maintained on STO feeder cells and mechanically passaged to fresh feeder cells every 7 days. DF-derived iPSCs were used for further gene knockout (KO) experiments.

### Multiplex genome editing in iPSCs

For gene KO, RNP mixtures of CRISPR sgRNA (*B2M*, *CIITA*, *PDCD-1*, and *CTLA-4*; each 50 pmol) and nuclease protein (C9, C9R, and C9G; each 25 pmol) were introduced into iPSCs using the Neon transfection system (Thermo, Waltham, MA, US). sgRNA information for multiplex gene knockout is listed in Supplementary Table 7. After the introduction of the RNP mixture, iPSCs were cultured for 1 d or 3 d in mTeSR Plus medium (Stemcell Technologies, Vancouver, BC, CA) on Matrigel (Corning, New York, MA, US)-coated culture plates. Cells were pooled after 1 d or 3 d of electroporation and used for subsequent analysis.

### Statistical analyses

Results were analyzed using GraphPad (ver. Prime 9). Comparison of groups was analyzed using a Nested t-test and ANOVA.

## Supplementary Information

Supplementary Information.
