# Author Correction: Enhanced genome editing efficiency of CRISPR PLUS: Cas9 chimeric fusion proteins

**DOI:** 10.1038/s41598-021-98186-3

**Published:** 2021-09-10

**Authors:** Jongjin Park, Jiyoung Yoon, Daekee Kwon, Mi-Jung Han, Sunmee Choi, Slki Park, Junghyuk Lee, Kiwook Lee, Jaehwan Lee, Seunghee Lee, Kyung-Sun Kang, Sunghwa Choe

**Affiliations:** 1 G+FLAS Life Sciences, CRISPR PLUS Lab, 38 Nakseong-daero, Gwanak-Gu, Seoul, 08790 Korea; 2 Naturegenic Inc, 1281 Win Hentschel Boulevard, Kurz Purdue Technology Center Suite 1573, West Lafayette, IN 47906 USA; 3 Stem Cells and Regenerative Bioengineering Institute in Kangstem Biotech, Gwangmyeong SK TechnoPark, Gwangmyeong-si, 14322 Gyeonggi-do Korea; 4 Adult Stem Cell Research Center, College of Veterinary Medicine, Seoul National University, Seoul, 08826 Korea; 5 School of Biological Sciences, College of Natural Sciences, Seoul National University, Gwanak-gu, Seoul, 08826 Korea

Correction to: *Scientific Reports* 10.1038/s41598-021-95406-8, published online 10 August 2021

The Supplementary Information file published with the original version of this Article was incomplete. It now also contains Tables A-I, and DNA sequences for eight CRISPR-PLUS proteins. The original Supplementary Information file is provided below.

This error has now been corrected in the Supplementary Information file that accompanies the original Article.

## Supplementary Information

Supplementary Information.

