# Supplementary material for: Enhanced genome editing efficiency of CRISPR PLUS: Cas9 chimeric fusion proteins
Source: Sci Rep. 2021 Aug 10;11:16199. doi: 10.1038/s41598-021-95406-8 (PMC8355345; doi:10.1038/s41598-021-95406-8)
Supplement: Supplementary file 1 — Supplementary Information. [file 41598_2021_95406_MOESM1_ESM.pdf]

Supplementary Information

S1 Fig. SDS-PAGE of SpyCas9 and the fusion proteins.

An SDS-PAGE gel was loaded with 8 μg of each protein after purification. The left- and rightmost lanes display molecular weight size markers, indicated in kDa. The gel image was stained with Coomassie Brilliant Blue. The molecular weights in kDa of SpyCas9, SpyCas9-RecJ, SpyCas9-GFP, SpyCas9-RecE, SpyCas9-T5, SpyCas9-Lambda, SpyCas9-mungbean, and SpyCas9-hTdT were 167, 230, 194, 263, 198, 193, 207, and 225, respectively. The protein yields during the purification process are given in S1 Text.

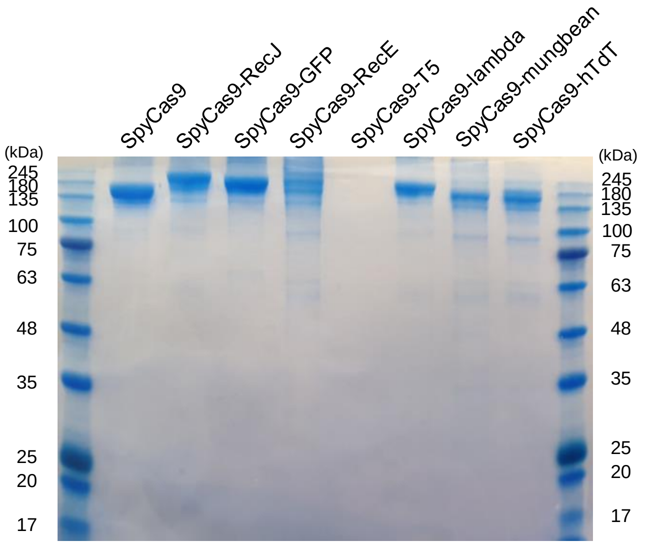

S2 Fig. Functional validation of the exonuclease activity of the RecJ moiety in SpyCas9-RecJ and green fluorescence in SpyCas9-GFP. (A) Exonuclease activity of C9R. RecJ exonuclease activity of the SpyCas9-RecJ fusion protein was tested on ssDNA and dsDNA in the presence or absence of magnesium ions. The DNA band intensity was diminished only when C9R protein acted with ssDNA in the presence of magnesium ions (right most lane). (B) Emission of green light by C9G. C9G showed a major excitation peak at a wavelength of 395 nm and a minor one at 475 nm, and an emission peak was observed at 509 nm (solid lines). C9 displayed only basal level of absorption and emission (broken lines).

a

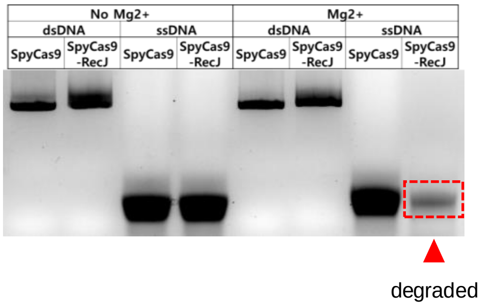

b

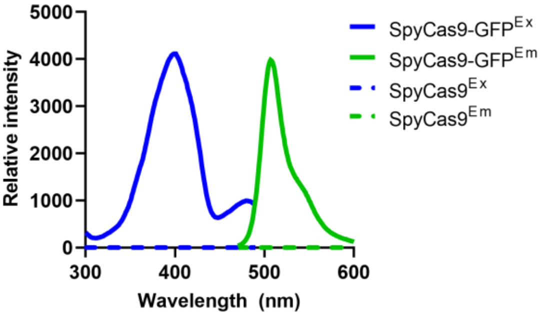

S3 Fig. The off-target effects of SpyCas9-RecJ were not significantly different from those of Cas9. For each target gene, five putative off-target sites were identified using Cas-OFFinder and subjected to NGS deep sequencing. The results show that the rates of mutations in the potential off-target sites were not significantly different from the conventional NGS sequencing error rate (0.01–0.1%).

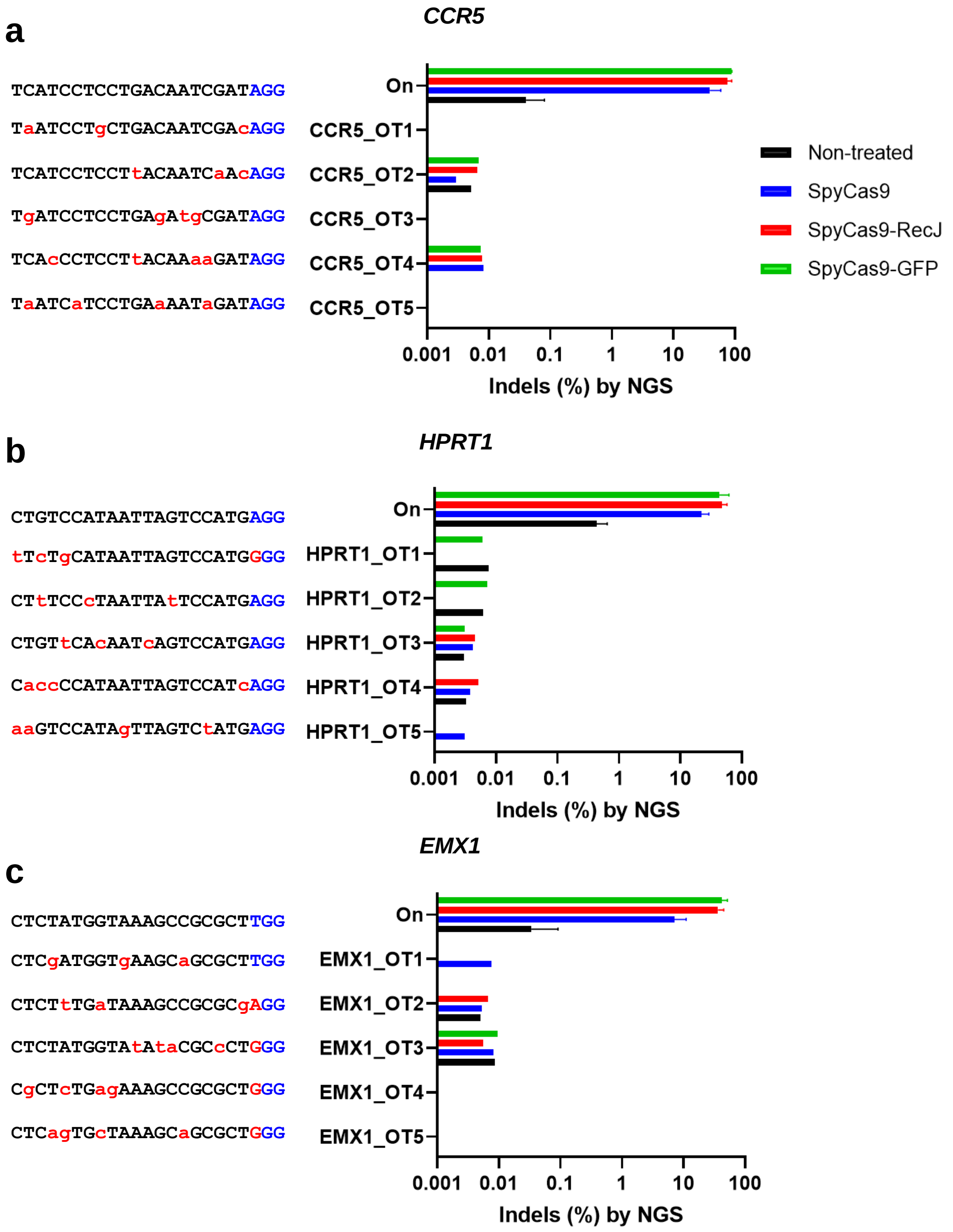

S4 Fig. DNA replacement activity in HEK293T cells.
HEK293T cells treated with C9 protein only, C9, C9R, and C9G (from left to right, lanes 1-4) targeting *CCR5*, *HPRT1*, and *EMX1* were harvested, and the DNA spanning the protospacer site was amplified, digested with *NdeI*, and resolved on an agarose gel (left to right). The substrates and cleaved products are indicated by red and blue triangles, respectively. The HDR activity was digitized and analyzed using ImageJ. HDR percentage is indicated bottom of each gel image.

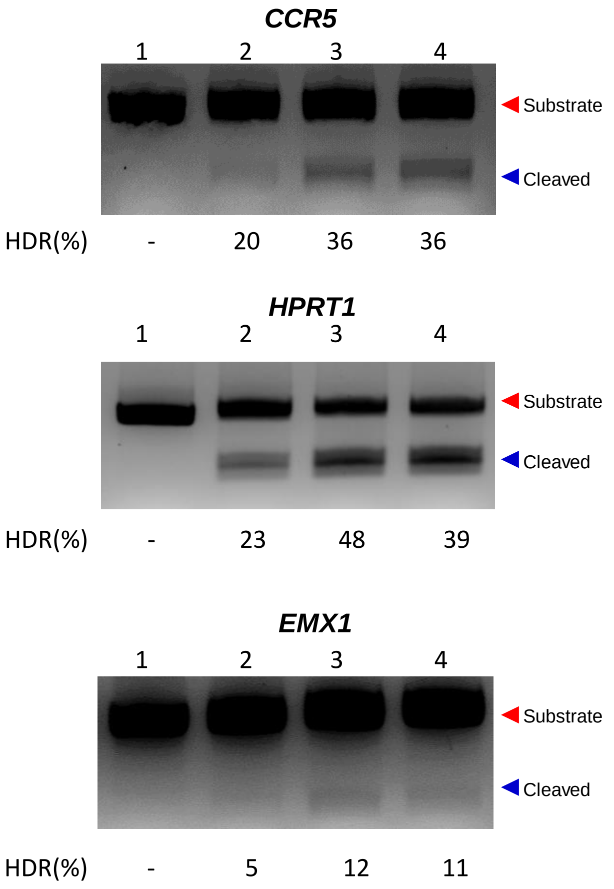

S5 Fig. Screening for sgRNAs targeting immune regulator genes. The genome editing efficiency of C9 and C9R was evaluated for two different sgRNAs for each of the target genes (*B2M*, *CIITA*, *CTLA4*, and *PDCD-1*). For each tested pair, an sgRNA showing greater indel efficiency (downward arrowheads) was chosen for the editing experiments with iPSCs. NT, non-treated cells as a negative control.

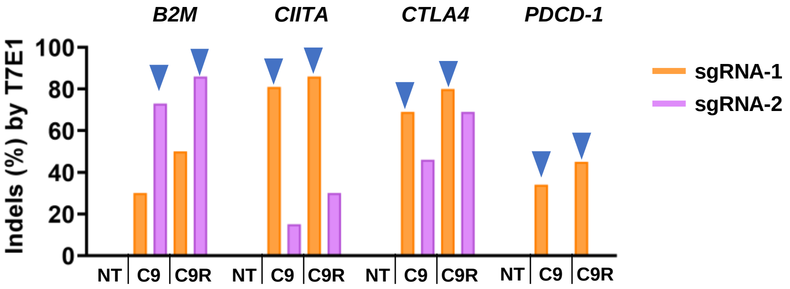

S6 Fig. T7E1 analysis of the indel efficiency after transfection of preassembled RNP for the four target genes. Three sets of four different RNPs, each pre-assembled with a different sgRNA (targeting *B2M*, *CIITA*, *CTLA4*, or *PDCD-1*) were simultaneously transfected into HEK293T cells and incubated for 3 d, and the T7E1 analysis was performed to determine the indel efficiencies. NT and M represent the non-treated control and DNA molecular weight marker, respectively. The substrates and cleaved products are indicated by red and blue triangles, respectively. The indel efficiency was digitized and analyzed using ImageJ. Indel efficiency in percentage is indicated bottom of each gel image.

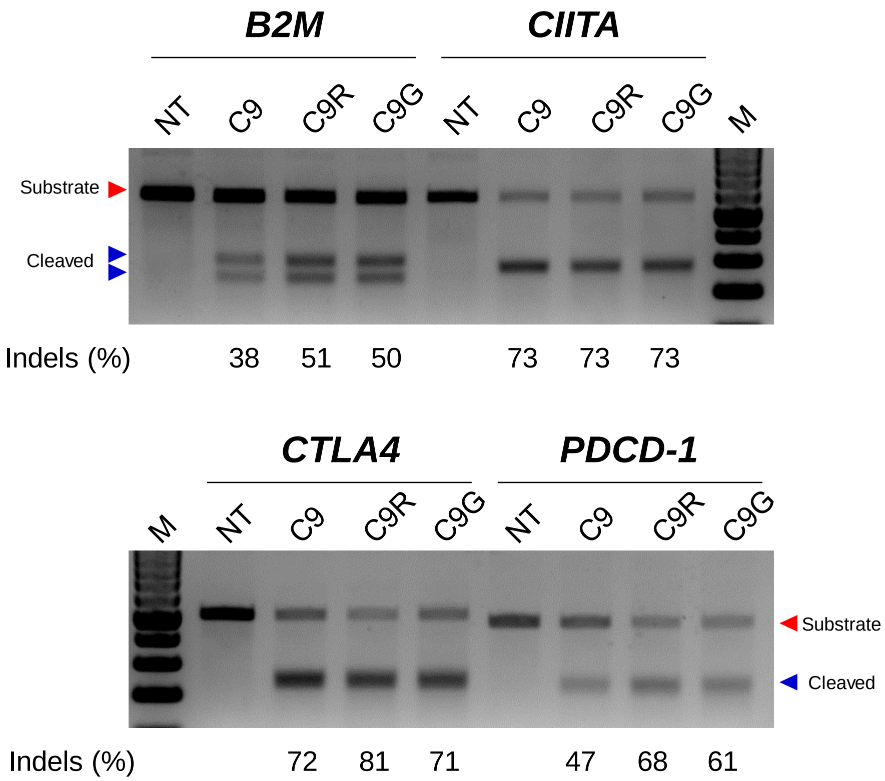

S7 Fig. Comparison of C9 and C9R activity in plant cells. Each C9 or C9R RNP preassembled with sgRNA targeting α-1,3-fucosyltransferase 1 (FucT13-1) was transfected into protoplasts derived from 4-week-old *N. benthamiana* leaves. More than 2.6-fold increase in indel efficiency of C9R relative to C9 was observed in targeted deep sequencing analysis. The error bars stand for standard deviations (SD), generated by a Nested *t*-test from GraphPad.

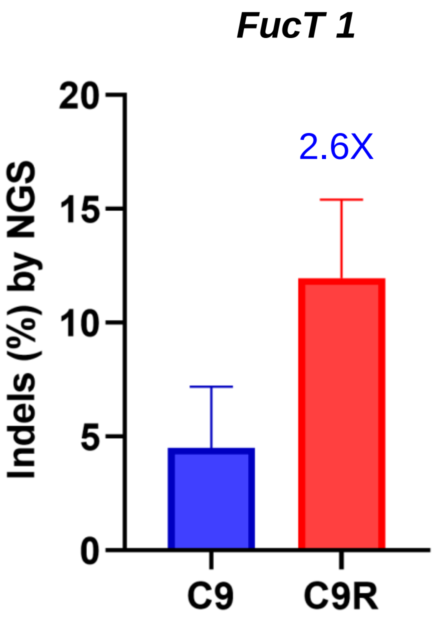

S8-S13 Fig. The full size of images, which were used in Fig 1B, S2, S4 and S6.

S8 Fig. The full size of image, which was used in Fig 1B

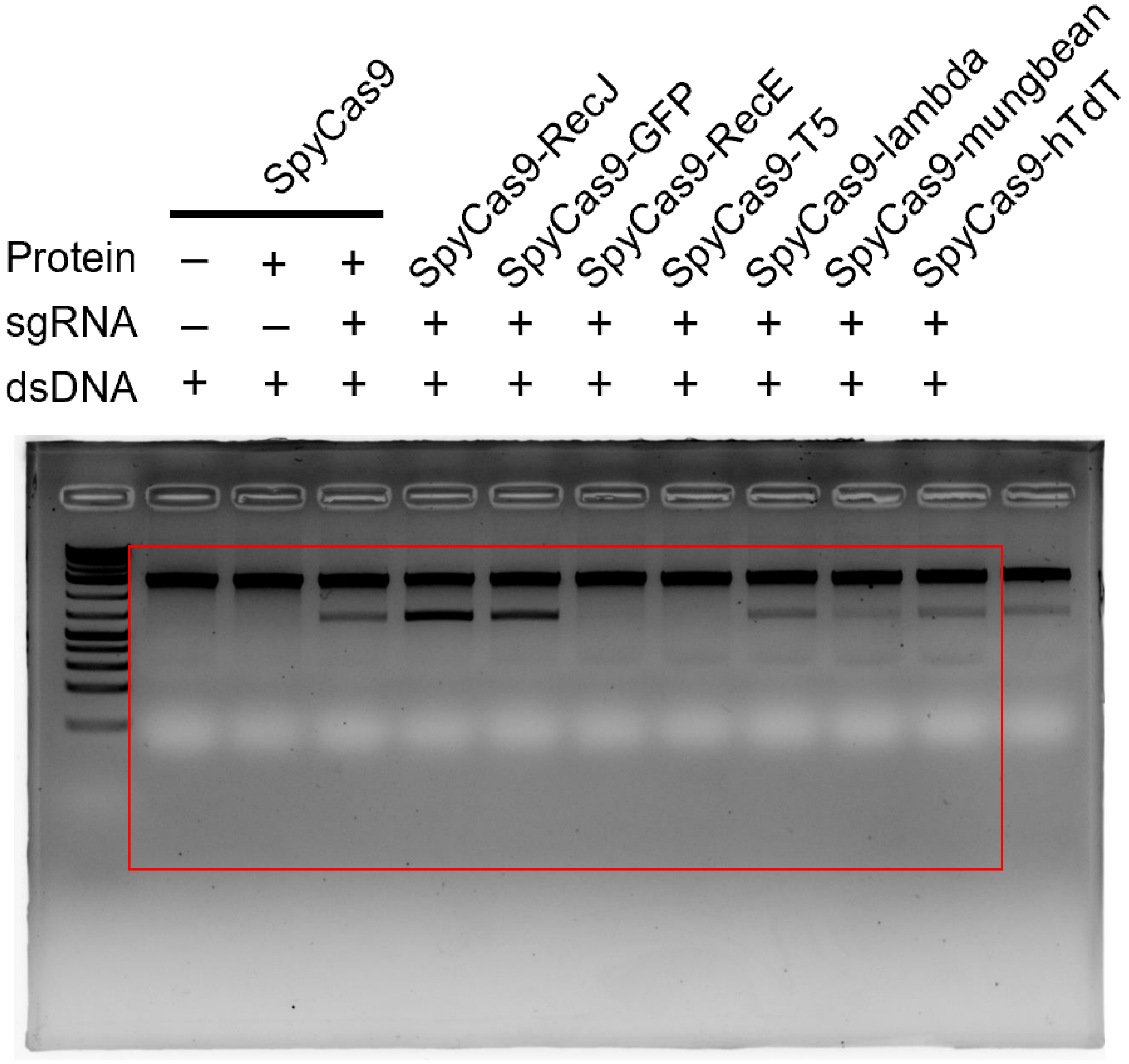

S9 Fig. The full size of image, which was used in S2 Fig.

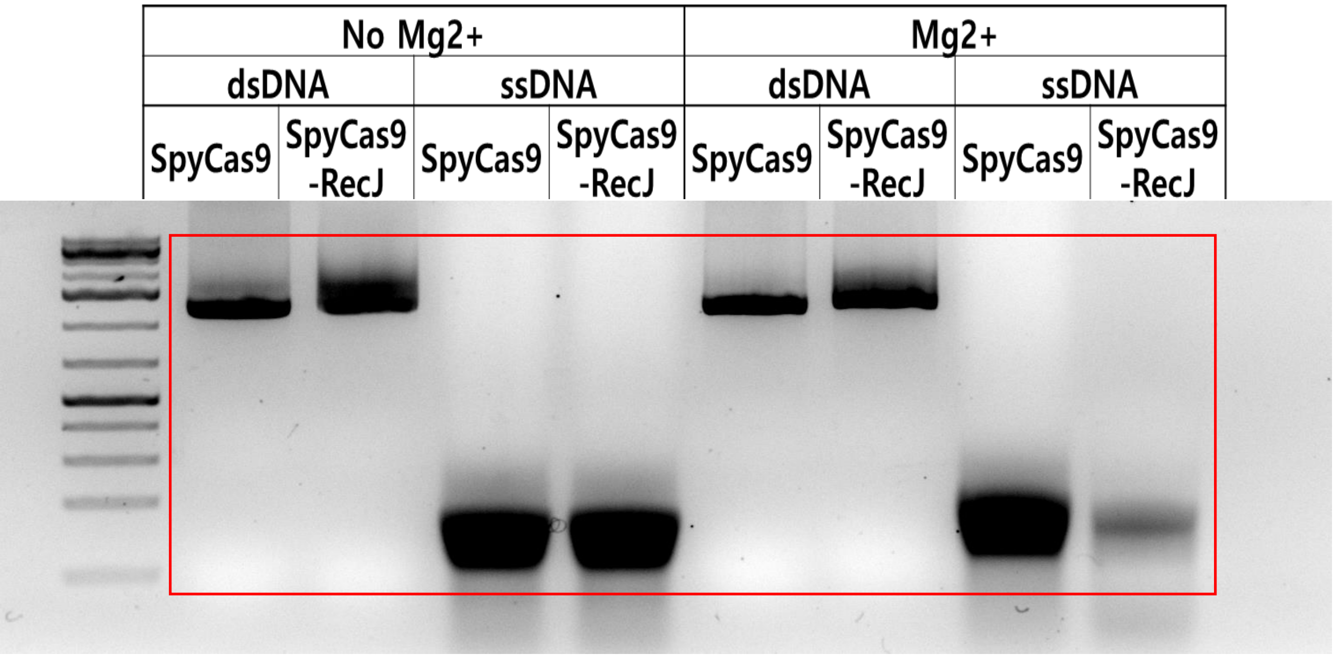

S10 Fig. The full size of image, which was used in S4 Fig.

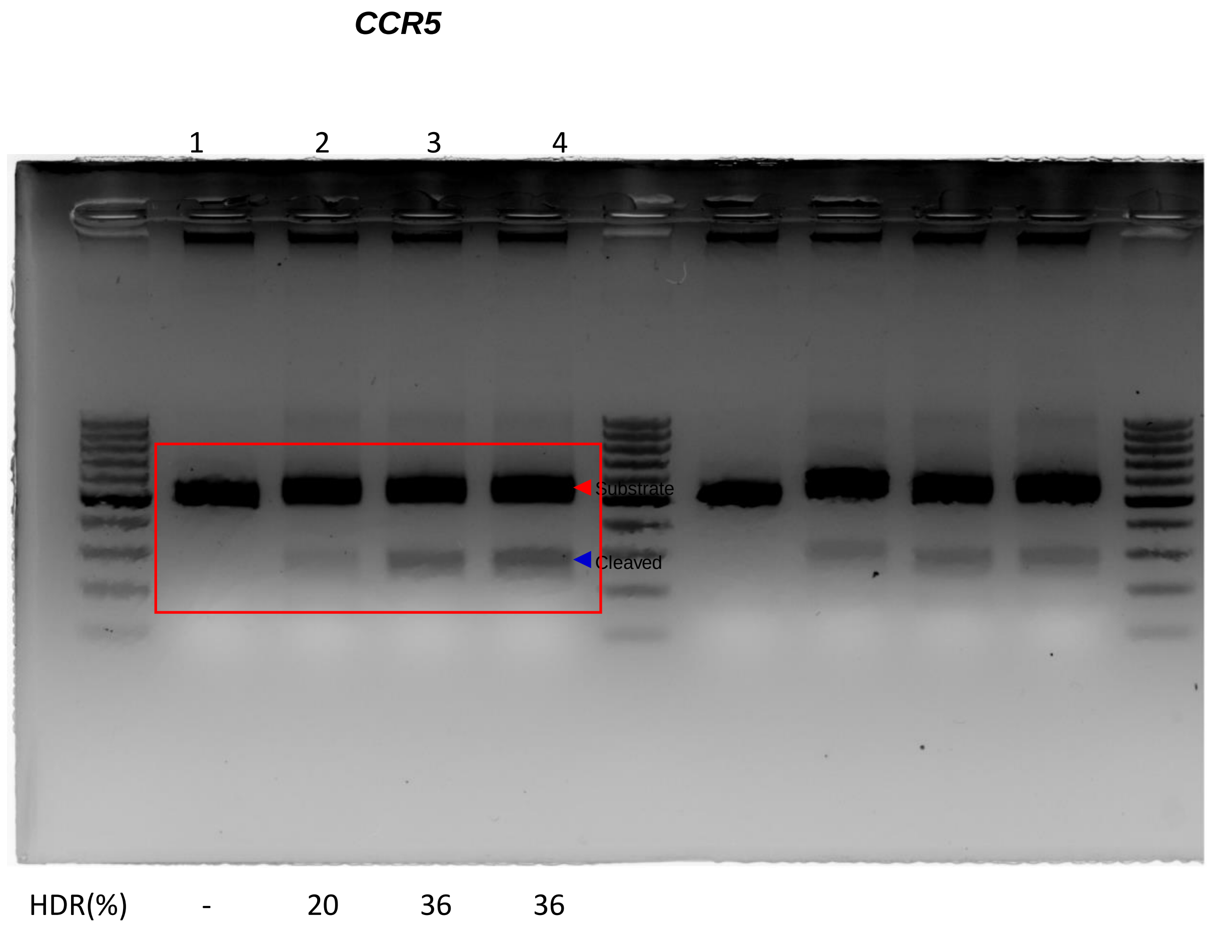

S11 Fig. The full size of image, which was used in S4 Fig.

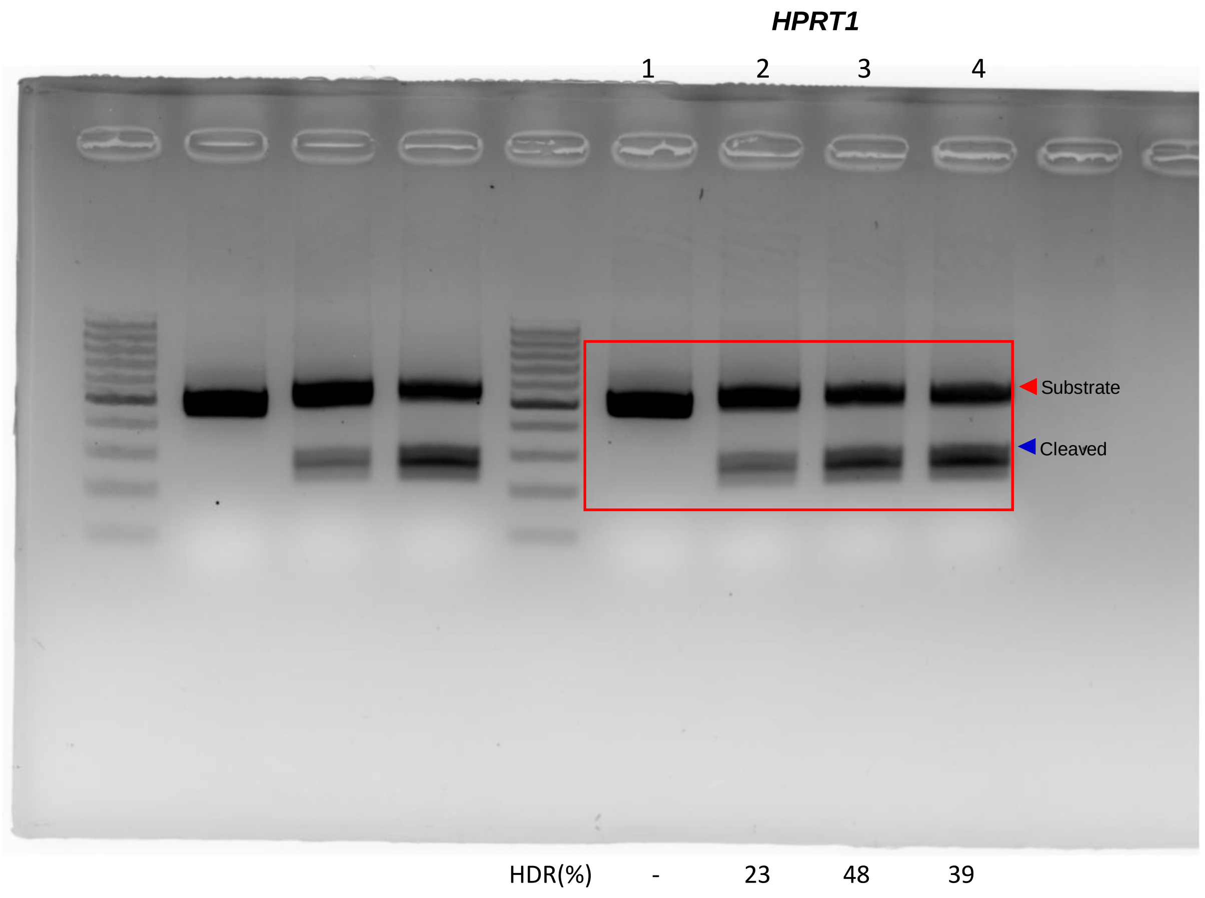

S12 Fig. The full size of image, which was used in S4 Fig.

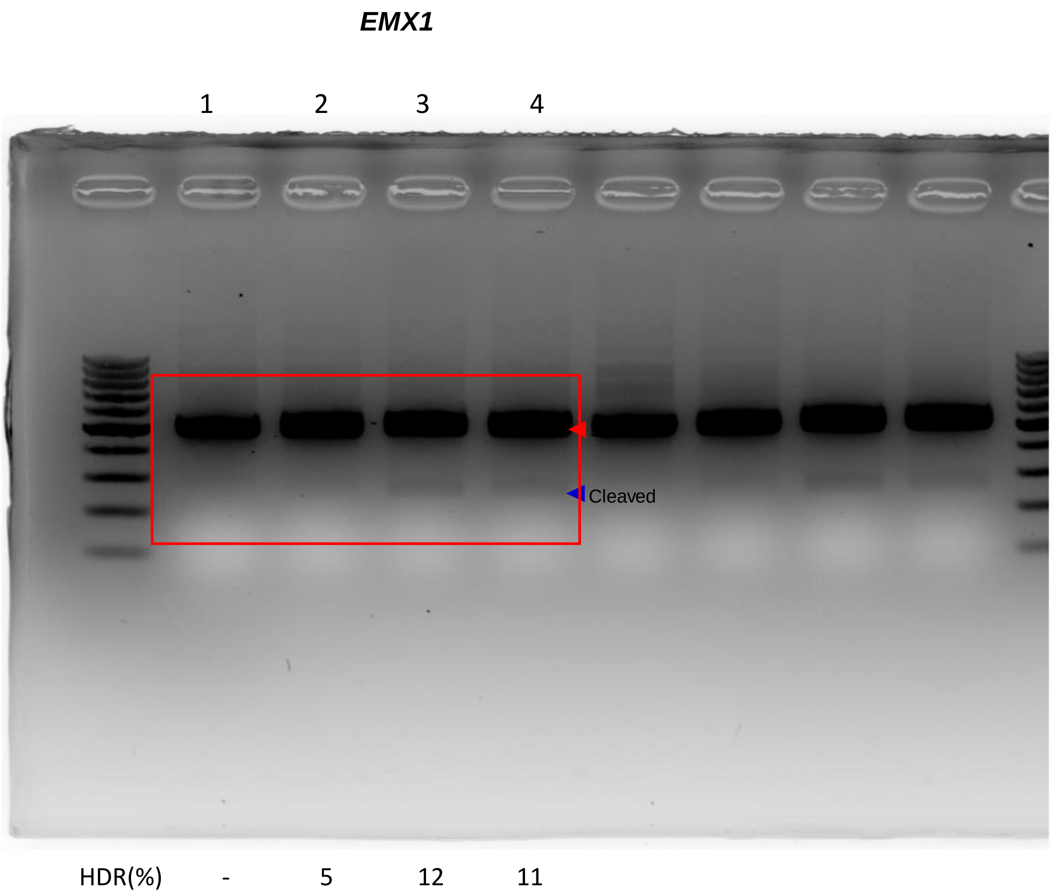

S13 Fig. The full size of image, which was used in S6 Fig.

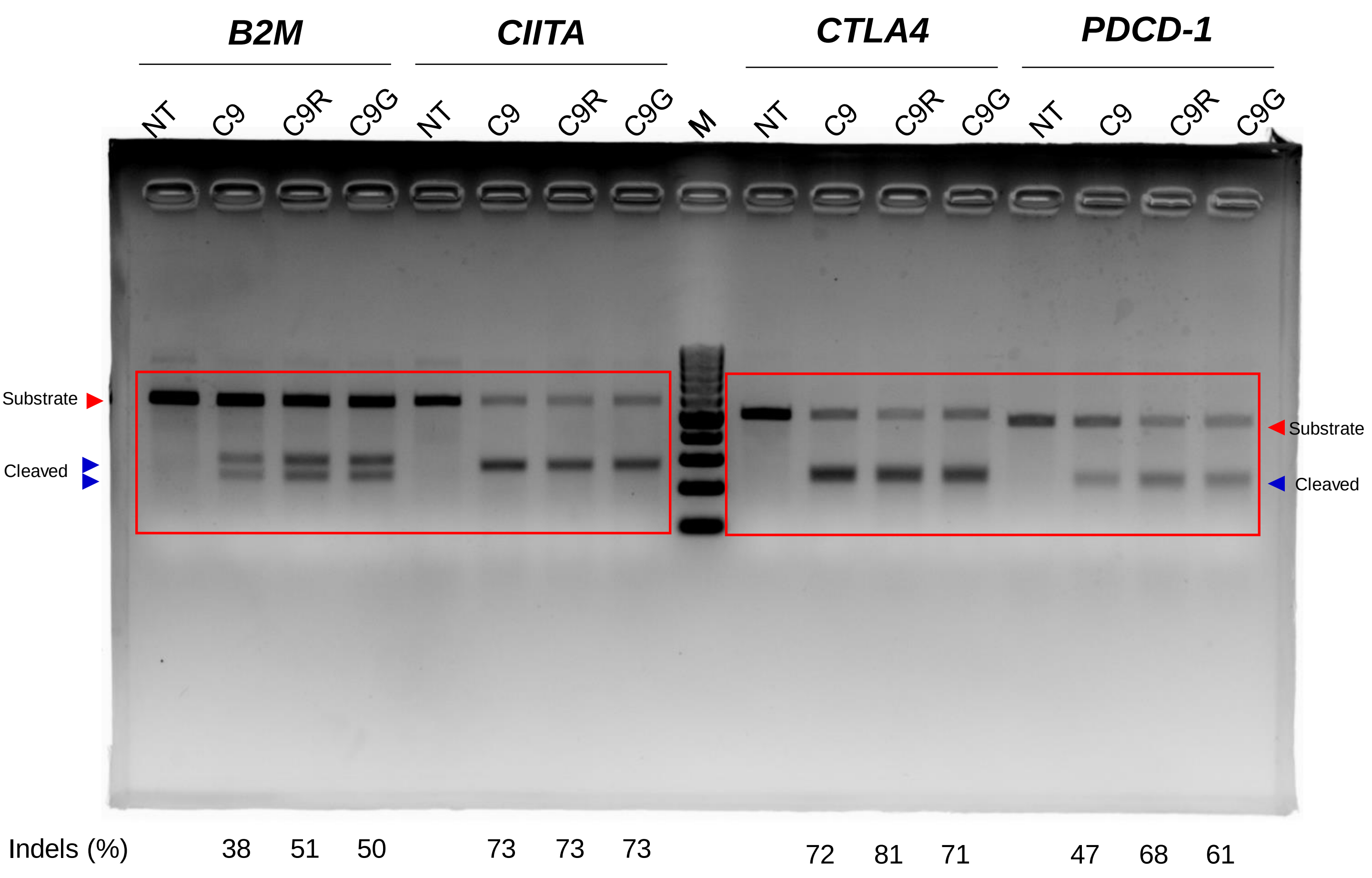

S1 Text,

Table A. Accessory proteins used to formulate CRISPR PLUS chimeric proteins.

	CRISPR PLUS Name	Added moiety	Representing origin of added moiety	Function of added moiety	Substrate DNA	Product	Intended enhancement	Enablement
1	SpyCas9-RecJ	RecJ	*Escherichia coli*	5'→3' exonuclease	ssDNA	blunt end/ 5’ and 3’ overhang	Indel and HR	Knock-out & Knock-in
2	SpyCas9-RecE	RecE	*Escherichia coli*	5'→3' exonuclease	dsDNA	3' overhang	Indel and HR	Knock-out & Knock-in
3	SpyCas9-T5	T5	Bacteriophage T5	5'→3' exonuclease	dsDNA and ssDNA	3' overhang	Indel and HR	Knock-out & Knock-in
4	SpyCas9-Lambda	Lexo	*Escherichia coli*	Lambda exonuclease, 5′→3′ exonuclease	5′-ⓟ blunt or recessed ends.	3' overhang	Indel and HR	Knock-out & Knock-in
5	SpyCas9-Mungbean	Mungbean nuclease	*Vigna radiata*	Single strand DNA digestion	ssDNA	blunt end	Indel and HR	Knock-out & Knock-in
6	SpyCas9-TdT	TdT	Human (*homo sipens*)	Terminal deoxyribonucleotidyl transferase	DSB, dsDNA/ssDNA	insertion of dNTP	Indel, labeling DSB with BrdU and subsequent sequencing to detect off target effect	Knock-out & Knock-in, Off-target detection
7	SpyCas9-GFP	GFP	*Aequorea victoria*	Green fluorescent protein	Not applicable	Not applicable	Not applicable	Not applicable

Table B. Molecular weight and protein purification yield of SpyCas9 and CRISPR PLUS

CRISPR PLUS	Molecular weight (kDa)	Yield (mg/L)
SpyCas9	166.56	8.5
SpyCas9-RecJ	230.06	3.9
SpyCas9-GFP	193.58	5.0
SpyCas9-RecE	263.15	0.7
SpyCas9-T5	198.45	0.2
SpyCas9-lambda	192.69	4.2
SpyCas9-mungbean	207.33	1.2
SpyCas9-hTdT	225.22	3.9

Table C. Sequences of single-stranded oligodeoxynucleotides (ssODN) with phosphorothioate bond in the first and last two nucleotides that were used as a repair template.

Target gene	ssODN sequence	Restriction site
CCR5	GGGCTCTATTTTATAGGCTTCTTCTCTGGAATCTTCTTCATCATCCTCCTGACAATCCATATGGATAGGTACCTGGCTGTCGTCCATGCTGTGTTTGCTTTAAAAG	Nde1
HPRT1	CAAGAAAAAGTAAAAAACCTCATTTTAAGATCTTACTTACCTGTCCATAATTAGTCCCATATGATGAGGAATAAACACCCTTTCCAAATCCTCAGCATAATGATTA	Nde1
EMX1	CAGTGCCCCCGCCGGTGCCGCCGTCCTTGGCCACCAAGGACTCTATGGTAAAGCCGCATATGGCTTGGCCGCGGGCTGGAACATGGTCGCAGCCGCGGGAGCTGT	Nde1

Table D. Protospacer and PAM sequences in the target genes.

Target gene	Protospacer Sequence (5' to 3')
HsCCR5	TCATCCTCCTGACAATCGAT[AGG]
HsHPRT1	CTGTCCATAATTAGTCCATG[AGG]
HsEMX1	CTCTATGGTAAAGCCGCGCT[TGG]
NbFTa13-1	GGCTTTTGAGAATTCTAATG[AGG]

Table E. Oligonucleotide list for PCR amplification of the target sites.

Target gene	Round	Sequence (5' to 3') forward	Sequence (5' to 3') reverse
CCR5	1st	GATGCTTAGAACAGTGATTGG	CCAAAGAATTCCTGGAAGGTG
	2nd	CTCCGCTCTACTCACTGGTG	GAGTAGCAGATGACCATGACAAGC
HPRT1	1st	CACTGTAATTGACTTAACAGAAAATT TTCAAGCTAGATGT	GTTTGTTTTGTTTTTTTGAGACAGA GTCTTGCTCTGTTT
	2nd	TACAGAGTCCCACTATACCACAACT GATACTAAGT	CCCGGCCTGTTGTTTTCTTACATAAT TCATTATCA
EMX1	1st	TTTTCTAGAAAAGCCTGGAGGTCTC	AAGTTCTCTGAGAACTGAAACGACA
	2nd	TTCATGGCCTCGGGGAACAC	TGGCCGTACGCTGTGGC
NbFucT13-1	1st	AATACTTTACGAATCCACTTCGC	CAAAGAGGATGTGAATGTGATCGAG

Table F. Oligonucleotide list for NGS to analyze On- and Off-target effects

Forward adapter overhangs: TCGTCGGCAGCGTCAGATGTGTATAAGAGACAG

Reverse adapter overhangs: GTCTCGTGGGCTCGGAGATGTGTATAAGAGACAG

Target gene	Primer name	Sequence (5' to 3') Forward (without adapters)	Sequence (5' to 3') Reverse (without adapters)
CCR5	On	TCACTATGCTGCCGCCCAGT	ACACAGCCACCACCCAAGTG
	Off#1	TCAGGGGGCATAACTGGGTT	ATTCAGGAAGAGCTGCTAGC
	Off#2	GGACAGAGCTATGAGGACTTAG	TATTTTTCACTGTAATTTCGTTTTGCC
	Off#3	TTTCATCACCTACCCATCTAGCC	AAGGAACAGGCAACAGGAGA
	Off#4	CACTCAGACTTGGCACAGAT	ATATATAGCTTTGTGATAGCCTTGTT
	Off#5	GCTCACCAGGCCTGCCTTT	CTCAGTCACCAGGCTCCACT
HPRT1	On	TAGCAAGTACTCAGAACAGCTG	ATATTTCTTTTTCAGATTAGTGATGATGA
	Off#1	TCATCCTGCTTCTCCCTACAA	GTGACACAGTCTGAGGTTTTAA
	Off#2	TTTGTTTGTATGTAGCCACTAGT	TTTTTGAACTATGCTCTTGAAAGT
	Off#3	AGTACAACCCCCCTTGAACA	CCAGCATCATGATTAGTGATGAT
	Off#4	CTGGCCAAGTCTAATTCCTTTT	ACCGTTTTGGCCTGATGATG
	Off#5	AACAAACCTGAGGAAGCTCA	GCACTTTCTTGCATGGAAATATT
EMX1	On	GTGGGCCGGAGCGGTTCC	TGCACACCCCGCAAGGCG
	Off#1	GAGTTAGCGTAGCTGAGTGC	AATTCTCGTCCGTCCTCGC
	Off#2	TTGGCCTGACTTGCCGGATG	ATTCCTTCACCCCTCCGGAG
	Off#3	CATAAATCCCCCGCTTAGGC	CAGTTTCTCAGAAAGAAACATTTAATAGGG
	Off#4	TGTCCACACCCACCAGGAGG	AATCTTCAACAACCACGGGAA
	Off#5	TTCATTTTATTTGTGTATCTCCTGTCCC	TGTGTAGGCCACACACTCG
NbFucT13-1	On	GCATTTGGTGTAGGTTTAGGCT	AATTCTGAAAATCCAAGTCTAT

Table G. Protospacer and PAM sequences for multiplex KO in iPSCs [PAM]

Target gene	Protospacer Sequence (5' to 3')
B2M-1	AGTCACATGGTTCACACGGC[AGG]
CIITA-1	GATATTGGCATAAGCCTCCC[TGG]
CTLA4-1	GTGCGGCAACCTACATGATG[GGG]
PDCD1-1	ATGTGGAAGTCACGCCCGTT[GGG]
**Target gene**	**Protospacer Sequence (5' to 3')**
B2M-2	ACTCACGCTGGATAGCCTCC[AGG]
CIITA-2	TTCCGAGGAACTTCTCGTCC[TGG]
CTLA4-2	ACACCGCTCCCATAAAGCCA[TGG]
PDCD1-2	ACCGCCCAGACGACTGGCCA[GGG]

Table H. Oligonucleotide list for multiplex gene PCR

Target gene	Round	Sequence (5' to 3') forward	Sequence (5' to 3') reverse
B2M-1	1st	TAGCCTCAGATACCAATCCAGC	TCTTGGGGCCAAATCATGTAG
	2nd	GCATCAGTATCTCAGCAGGTG	TGACACCAAGTTAGCCCCAA
CIITA-1	1st	AAACTTGGGTAGGTCGTTTCAC	TTTCCAACACCCTGTGAGGT
	2nd	GGAGGTGGAGGATGTAAACATG	CCCTCAGCTTGCTGTAGAGAC
CTLA4-1	1st	GTTCCGGAGCTATATAGCTCCT	TCTGGATCTGCAACAGAAAACA
	2nd	CCGTGGGGATGAAGCTAGAA	GACTGCAATGCAACAGGTGT
PDCD1-1	1st	AGGACCCAGACTAGCAGCA	TTACACTCCCCTGTGCCAGA
	2nd	GGAGCTCCTGATCCTGTGC	TCTCAGACTCCCCAGACAGG

Target gene	Round	Sequence (5' to 3') forward	Sequence (5' to 3') reverse
B2M-2	1st	ATTCGAAACCGCTTTGTATCACAGC	GCAGGGAATTCCCAAGCTGTAGTTA
	2nd	GACGCTTATCGACGCCCTAAACTTT	GCCTTCTTAAACATCACGAGACTCT
CIITA-2	1st	TTCCTGGAAAGCCTTTCTTACTCCC	GCCTAATCTGAGGCTCATTTCCATG
	2nd	AATCTGAGAACCTCCAGCATTTGCC	TGTAGACACAGGGATCTTGCTATGT
CTLA4-2	1st	AGACAAATCCTGCCATTAGCCCAAG	AACTTGTGAGCTCATCCTGAAACCC
	2nd	TCAGTTGAGTGCTTGAGGTTGTCTT	CCAGCCAAGCCAGATTGGAGTTTTA
PDCD1-2	1st	AAAACTCGAGTGAGGACCAAGGAT G	TCCGACAGAATGGGAGTGGGGTTGA
	2nd	GACTGAGAGTGAAAGGTCCCTCCAG	TGTCTCCCTGTCTCTGTCTCTCTCT

Table I. Oligonucleotide list for NGS to analyze KO effects in iPSCs

Forward adapter overhangs: TCGTCGGCAGCGTCAGATGTGTATAAGAGACAG

Reverse adapter overhangs: GTCTCGTGGGCTCGGAGATGTGTATAAGAGACAG

Target gene	Sequence (5' to 3') Forward (without adapters)	Sequence (5' to 3') Reverse (without adapters)
B2M-2	AGAACTTGGAGAAGGGAAGTCACG	TATAAGTGGAGGCGTCGCGC
CIITA-1	AATGGGTGATATTGACAAGTGGCAG	TCCTTCTTCATCCAAGGGACTTTTC
CTLA4-1	ATCGCCAGCTTTGTGTGTGA	TAGAGTCCCGTGTCCATGGC
PDCD1-1	CGCAGGCTCTCTTTGATCTG	GCTAAACTGGTACCGCATGA

S1 Text,

DNA sequences for eight CRISPR-PLUS proteins

1) SpyCas9

ATGGGCAGCAGCCATCATCATCATCATCACAGCAGCGGCCTGGTGCCGCGCGGCAGCCAT
ATGGCTAGCATGACTGGTGGACAGCAAATGGGTCGCGGATCCGAATTCGAGCTCCGTCGA
CAAGCTTGCGGCCGCATGGACAAGAAGTACAGCATCGGCCTGGACATCGGTACCAACAGC
GTGGGCTGGGCCGTGATCACCGACGAGTACAAGGTGCCCAGCAAGAAGTTCAAGGTGCT
GGGCAACACCGACCGCCACAGCATCAAGAAGAACCTGATCGGCGCCCTGCTGTTCGACA
GCGGCGAGACCGCCGAGGCCACCCGCCTGAAGCGCACCGCCCGCCGCCGCTACACCCGC
CGCAAGAACCGCATCTGCTACCTGCAGGAGATCTTCAGCAACGAGATGGCCAAGGTGGAC
GACAGCTTCTTCCACCGCCTGGAGGAGAGCTTCCTGGTGGAGGAGGACAAGAAGCACGA
GCGCCACCCCATCTTCGGCAACATCGTGGACGAGGTGGCCTACCACGAGAAGTACCCCAC
CATCTACCACCTGCGCAAGAAGCTGGTGGACAGCACCGACAAGGCCGACCTGCGCCTGAT
CTACCTGGCCCTGGCCCACATGATCAAGTTCCGCGGCCACTTCCTGATCGAGGGCGACCT
GAACCCCGACAACAGCGACGTGGACAAGCTGTTCATCCAGCTGGTGCAGACCTACAACC
AGCTGTTCGAGGAGAACCCCATCAACGCCAGCGGCGTGGACGCCAAGGCCATCCTGAGC
GCCCGCCTGAGCAAGAGCCGCCGCCTGGAGAACCTGATCGCCCAGCTGCCCGGCGAGAA
GAAGAACGGCCTGTTCGGCAACCTGATCGCCCTGAGCCTGGGCCTGACCCCCAACTTCAA
GAGCAACTTCGACCTGGCCGAGGACGCCAAGCTGCAGCTGAGCAAGGACACCTACGACG
ACGACCTGGACAACCTGCTGGCCCAGATCGGCGACCAGTACGCCGACCTGTTCCTGGCCG
CCAAGAACCTGAGCGACGCCATCCTGCTGAGCGACATCCTGCGCGTGAACACCGAGATCA
CCAAGGCCCCCCTGAGCGCCAGCATGATCAAGCGCTACGACGAGCACCACCAGGACCTG
ACCCTGCTGAAGGCCCTGGTGCGCCAGCAGCTGCCCGAGAAGTACAAGGAGATCTTCTTC
GACCAGAGCAAGAACGGCTACGCCGGCTACATCGACGGCGGCGCCAGCCAGGAGGAGTT
CTACAAGTTCATCAAGCCCATCCTGGAGAAGATGGACGGCACCGAGGAGCTGCTGGTGAA
GCTGAACCGCGAGGACCTGCTGCGCAAGCAGCGCACCTTCGACAACGGCAGCATCCCCC
ACCAGATCCACCTGGGCGAGCTGCACGCCATCCTGCGCCGCCAGGAGGACTTCTACCCCT
TCCTGAAGGACAACCGCGAGAAGATCGAGAAGATCCTGACCTTCCGCATCCCCTACTACG
TGGGCCCCCTGGCCCGCGGCAACAGCCGCTTCGCCTGGATGACCCGCAAGAGCGAGGAG
ACCATCACCCCCTGGAACTTCGAGGAGGTGGTGGACAAGGGCGCCAGCGCCCAGAGCTT
CATCGAGCGCATGACCAACTTCGACAAGAACCTGCCCAACGAGAAGGTGCTGCCCAAGC
ACAGCCTGCTGTACGAGTACTTCACCGTGTACAACGAGCTGACCAAGGTGAAGTACGTGA
CCGAGGGCATGCGCAAGCCCGCCTTCCTGAGCGGCGAGCAGAAGAAGGCCATCGTGGAC
CTGCTGTTCAAGACCAACCGCAAGGTGACCGTGAAGCAGCTGAAGGAGGACTACTTCAA
GAAGATCGAGTGCTTCGACAGCGTGGAGATCAGCGGCGTGGAGGACCGCTTCAACGCCA
GCCTGGGCACCTACCACGACCTGCTGAAGATCATCAAGGACAAGGACTTCCTGGACAACG
AGGAGAACGAGGACATCCTGGAGGACATCGTGCTGACCCTGACCCTGTTCGAGGACCGC
GAGATGATCGAGGAGCGCCTGAAGACCTACGCCCACCTGTTCGACGACAAGGTGATGAA
GCAGCTGAAGCGCCGCCGCTACACCGGCTGGGGCCGCCTGAGCCGCAAGCTTATCAACG
GCATCCGCGACAAGCAGAGCGGCAAGACCATCCTGGACTTCCTGAAGAGCGACGGCTTC
GCCAACCGCAACTTCATGCAGCTGATCCACGACGACAGCCTGACCTTCAAGGAGGACATC
CAGAAGGCCCAGGTGAGCGGCCAGGGCGACAGCCTGCACGAGCACATCGCCAACCTGGC
CGGCAGCCCCGCCATCAAGAAGGGCATCCTGCAGACCGTGAAGGTGGTGGACGAGCTGG
TGAAGGTGATGGGCCGCCACAAGCCCGAGAACATCGTGATCGAGATGGCCCGCGAGAAC
CAGACCACCCAGAAGGGCCAGAAGAACAGCCGCGAGCGCATGAAGCGCATCGAGGAGG
GCATCAAGGAGCTGGGCAGCCAGATCCTGAAGGAGCACCCCGTGGAGAACACCCAGCTG
CAGAACGAGAAGCTGTACCTGTACTACCTGCAGAACGGCCGCGACATGTACGTGGACCAG
GAGCTGGACATCAACCGCCTGAGCGACTACGACGTGGACCACATCGTGCCCCAGAGCTTC

CTGAAGGACGACAGCATCGACAACAAGGTGCTGACCCGCAGCGACAAGAACCGCGGCA
AGAGCGACAACGTGCCCAGCGAGGAGGTGGTGAAGAAGATGAAGAACTACTGGCGCCA
GCTGCTGAACGCCAAGCTGATCACCCAGCGCAAGTTCGACAACCTGACCAAGGCCGAGC
GCGGCGGCCTGAGCGAGCTGGACAAGGCCGGCTTCATCAAGCGCCAGCTGGTGGAGACC
CGCCAGATCACCAAGCACGTGGCCCAGATCCTGGACAGCCGCATGAACACCAAGTACGA
CGAGAACGACAAGCTGATCCGCGAGGTGAAGGTGATCACCCTGAAGAGCAAGCTGGTGA
GCGACTTCCGCAAGGACTTCCAGTTCTACAAGGTGCGCGAGATCAACAACTACCACCACG
CCCACGACGCCTACCTGAACGCCGTGGTGGGCACCGCCCTGATCAAGAAGTACCCCAAGC
TGGAGAGCGAGTTCGTGTACGGCGACTACAAGGTGTACGACGTGCGCAAGATGATCGCCA
AGAGCGAGCAGGAGATCGGCAAGGCCACCGCCAAGTACTTCTTCTACAGCAACATCATGA
ACTTCTTCAAGACCGAGATCACCCTGGCCAACGGCGAGATCCGCAAGCGCCCCCTGATCG
AGACCAACGGCGAGACCGGCGAGATCGTGTGGGACAAGGGCCGCGACTTCGCCACCGTG
CGCAAGGTGCTGAGCATGCCCCAGGTGAACATCGTGAAGAAGACCGAGGTGCAGACCGG
CGGCTTCAGCAAGGAGAGCATCCTGCCCAAGCGCAACAGCGACAAGCTGATCGCCCGCA
AGAAGGACTGGGACCCCAAGAAGTACGGCGGCTTCGACAGCCCCACCGTGGCCTACAGC
GTGCTGGTGGTGGCCAAGGTGGAGAAGGGCAAGAGCAAGAAGCTGAAGAGCGTGAAGG
AGCTGCTGGGCATCACCATCATGGAGCGCAGCAGCTTCGAGAAGAACCCCATCGACTTCC
TGGAGGCCAAGGGCTACAAGGAGGTGAAGAAGGACCTGATCATCAAGCTGCCCAAGTAC
AGCCTGTTCGAGCTGGAGAACGGCCGCAAGCGCATGCTGGCCAGCGCCGGCGAGCTGCA
GAAGGGCAACGAGCTGGCCCTGCCCAGCAAGTACGTGAACTTCCTGTACCTGGCCAGCC
ACTACGAGAAGCTGAAGGGCAGCCCCGAGGACAACGAGCAGAAGCAGCTGTTCGTGGA
GCAGCACAAGCACTACCTGGACGAGATCATCGAGCAGATCAGCGAGTTCAGCAAGCGCG
TGATCCTGGCCGACGCCAACCTGGACAAGGTGCTGAGCGCCTACAACAAGCACCGCGAC
AAGCCCATCCGCGAGCAGGCCGAGAACATCATCCACCTGTTCACCCTGACCAACCTGGGC
GCCCCCGCCGCCTTCAAGTACTTCGACACCACCATCGACCGCAAGCGCTACACCAGCACC
AAGGAGGTGCTGGACGCCACCCTGATCCACCAGAGCATCACCGGTCTGTACGAGACCCGC
ATCGACCTGAGCCAGCTGGGCGGCGACGCGGCCGCACTCGACCTCGAGAAAAGGCCGGC
GGCCACGAAAAAGGCCGGCCAGGCAAAAAAGAAAGAGCACCACCACCACCACCACTGA

2) SpyCas9-RecJ

ATGGGCAGCAGCCATCATCATCATCATCACAGCAGCGGCCTGGTGCCGCGCGGCAGCCAT
ATGGCTAGCATGACTGGTGGACAGCAAATGGGTCGCGGATCCGAATTCGAGCTCCGTCGA
CAAGCTTGCGGCCGCATGGACAAGAAGTACAGCATCGGCCTGGACATCGGTACCAACAGC
GTGGGCTGGGCCGTGATCACCGACGAGTACAAGGTGCCCAGCAAGAAGTTCAAGGTGCT
GGGCAACACCGACCGCCACAGCATCAAGAAGAACCTGATCGGCGCCCTGCTGTTCGACA
GCGGCGAGACCGCCGAGGCCACCCGCCTGAAGCGCACCGCCCGCCGCCGCTACACCCGC
CGCAAGAACCGCATCTGCTACCTGCAGGAGATCTTCAGCAACGAGATGGCCAAGGTGGAC
GACAGCTTCTTCCACCGCCTGGAGGAGAGCTTCCTGGTGGAGGAGGACAAGAAGCACGA
GCGCCACCCCATCTTCGGCAACATCGTGGACGAGGTGGCCTACCACGAGAAGTACCCCAC
CATCTACCACCTGCGCAAGAAGCTGGTGGACAGCACCGACAAGGCCGACCTGCGCCTGAT
CTACCTGGCCCTGGCCCACATGATCAAGTTCCGCGGCCACTTCCTGATCGAGGGCGACCT
GAACCCCGACAACAGCGACGTGGACAAGCTGTTCATCCAGCTGGTGCAGACCTACAACC
AGCTGTTCGAGGAGAACCCCATCAACGCCAGCGGCGTGGACGCCAAGGCCATCCTGAGC
GCCCGCCTGAGCAAGAGCCGCCGCCTGGAGAACCTGATCGCCCAGCTGCCCGGCGAGAA
GAAGAACGGCCTGTTCGGCAACCTGATCGCCCTGAGCCTGGGCCTGACCCCCAACTTCAA
GAGCAACTTCGACCTGGCCGAGGACGCCAAGCTGCAGCTGAGCAAGGACACCTACGACG
ACGACCTGGACAACCTGCTGGCCCAGATCGGCGACCAGTACGCCGACCTGTTCCTGGCCG
CCAAGAACCTGAGCGACGCCATCCTGCTGAGCGACATCCTGCGCGTGAACACCGAGATCA

CCAAGGCCCCCTGAGCGCCAGCATGATCAAGCGCTACGACGAGCACCACCAGGACCTG
ACCCTGCTGAAGGCCCTGGTGCGCCAGCAGCTGCCCGAGAAGTACAAGGAGATCTTCTTC
GACCAGAGCAAGAACGGCTACGCCGGCTACATCGACGGCGGCGCCAGCCAGGAGGAGTT
CTACAAGTTCATCAAGCCCATCCTGGAGAAGATGGACGGCACCGAGGAGCTGCTGGTGAA
GCTGAACCGCGAGGACCTGCTGCGCAAGCAGCGCACCTTCGACAACGGCAGCATCCCCC
ACCAGATCCACCTGGGCGAGCTGCACGCCATCCTGCGCCGCCAGGAGGACTTCTACCCCT
TCCTGAAGGACAACCGCGAGAAGATCGAGAAGATCCTGACCTTCCGCATCCCCTACTACG
TGGGCCCCCTGGCCCGCGGCAACAGCCGCTTCGCCTGGATGACCCGCAAGAGCGAGGAG
ACCATCACCCCCTGGAACTTCGAGGAGGTGGTGGACAAGGGCGCCAGCGCCCAGAGCTT
CATCGAGCGCATGACCAACTTCGACAAGAACCTGCCCAACGAGAAGGTGCTGCCCAAGC
ACAGCCTGCTGTACGAGTACTTCACCGTGTACAACGAGCTGACCAAGGTGAAGTACGTGA
CCGAGGGCATGCGCAAGCCCGCCTTCCTGAGCGGCGAGCAGAAGAAGGCCATCGTGGAC
CTGCTGTTCAAGACCAACCGCAAGGTGACCGTGAAGCAGCTGAAGGAGGACTACTTCAA
GAAGATCGAGTGCTTCGACAGCGTGGAGATCAGCGGCGTGGAGGACCGCTTCAACGCCA
GCCTGGGCACCTACCACGACCTGCTGAAGATCATCAAGGACAAGGACTTCCTGGACAACG
AGGAGAACGAGGACATCCTGGAGGACATCGTGCTGACCCTGACCCTGTTCGAGGACCGC
GAGATGATCGAGGAGCGCCTGAAGACCTACGCCCACCTGTTCGACGACAAGGTGATGAA
GCAGCTGAAGCGCCGCCGCTACACCGGCTGGGGCCGCCTGAGCCGCAAGCTTATCAACG
GCATCCGCGACAAGCAGAGCGGCAAGACCATCCTGGACTTCCTGAAGAGCGACGGCTTC
GCCAACCGCAACTTCATGCAGCTGATCCACGACGACAGCCTGACCTTCAAGGAGGACATC
CAGAAGGCCCAGGTGAGCGGCCAGGGCGACAGCCTGCACGAGCACATCGCCAACCTGGC
CGGCAGCCCCGCCATCAAGAAGGGCATCCTGCAGACCGTGAAGGTGGTGGACGAGCTGG
TGAAGGTGATGGGCCGCCACAAGCCCGAGAACATCGTGATCGAGATGGCCCGCGAGAAC
CAGACCACCCAGAAGGGCCAGAAGAACAGCCGCGAGCGCATGAAGCGCATCGAGGAGG
GCATCAAGGAGCTGGGCAGCCAGATCCTGAAGGAGCACCCCGTGGAGAACACCCAGCTG
CAGAACGAGAAGCTGTACCTGTACTACCTGCAGAACGGCCGCGACATGTACGTGGACCAG
GAGCTGGACATCAACCGCCTGAGCGACTACGACGTGGACCACATCGTGCCCCAGAGCTTC
CTGAAGGACGACAGCATCGACAACAAGGTGCTGACCCGCAGCGACAAGAACCGCGGCA
AGAGCGACAACGTGCCCAGCGAGGAGGTGGTGAAGAAGATGAAGAACTACTGGCGCCA
GCTGCTGAACGCCAAGCTGATCACCCAGCGCAAGTTCGACAACCTGACCAAGGCCGAGC
GCGGCGGCCTGAGCGAGCTGGACAAGGCCGGCTTCATCAAGCGCCAGCTGGTGGAGACC
CGCCAGATCACCAAGCACGTGGCCCAGATCCTGGACAGCCGCATGAACACCAAGTACGA
CGAGAACGACAAGCTGATCCGCGAGGTGAAGGTGATCACCCTGAAGAGCAAGCTGGTGA
GCGACTTCCGCAAGGACTTCCAGTTCTACAAGGTGCGCGAGATCAACAACTACCACCACG
CCCACGACGCCTACCTGAACGCCGTGGTGGGCACCGCCCTGATCAAGAAGTACCCCAAGC
TGGAGAGCGAGTTCGTGTACGGCGACTACAAGGTGTACGACGTGCGCAAGATGATCGCCA
AGAGCGAGCAGGAGATCGGCAAGGCCACCGCCAAGTACTTCTTCTACAGCAACATCATGA
ACTTCTTCAAGACCGAGATCACCCTGGCCAACGGCGAGATCCGCAAGCGCCCCCTGATCG
AGACCAACGGCGAGACCGGCGAGATCGTGTGGGACAAGGGCCGCGACTTCGCCACCGTG
CGCAAGGTGCTGAGCATGCCCCAGGTGAACATCGTGAAGAAGACCGAGGTGCAGACCGG
CGGCTTCAGCAAGGAGAGCATCCTGCCCAAGCGCAACAGCGACAAGCTGATCGCCCGCA
AGAAGGACTGGGACCCCAAGAAGTACGGCGGCTTCGACAGCCCCACCGTGGCCTACAGC
GTGCTGGTGGTGGCCAAGGTGGAGAAGGGCAAGAGCAAGAAGCTGAAGAGCGTGAAGG
AGCTGCTGGGCATCACCATCATGGAGCGCAGCAGCTTCGAGAAGAACCCCATCGACTTCC
TGGAGGCCAAGGGCTACAAGGAGGTGAAGAAGGACCTGATCATCAAGCTGCCCAAGTAC
AGCCTGTTCGAGCTGGAGAACGGCCGCAAGCGCATGCTGGCCAGCGCCGGCGAGCTGCA
GAAGGGCAACGAGCTGGCCCTGCCCAGCAAGTACGTGAACTTCCTGTACCTGGCCAGCC
ACTACGAGAAGCTGAAGGGCAGCCCCGAGGACAACGAGCAGAAGCAGCTGTTCGTGGA
GCAGCACAAGCACTACCTGGACGAGATCATCGAGCAGATCAGCGAGTTCAGCAAGCGCG
TGATCCTGGCCGACGCCAACCTGGACAAGGTGCTGAGCGCCTACAACAAGCACCGCGAC

AAGCCCATCCGCGAGCAGGCCGAGAACATCATCCACCTGTTCACCCTGACCAACCTGGGC
GCCCCCGCCGCCTTCAAGTACTTCGACACCACCATCGACCGCAAGCGCTACACCAGCACC
AAGGAGGTGCTGGACGCCACCCTGATCCACCAGAGCATCACCGGTCTGTACGAGACCCGC
ATCGACCTGAGCCAGCTGGGCGGCGACGCGGCCGCACTCGACCTGCAGGTGAAACAACA
GATACAACTTCGTCGCCGTGAAGTCGATGAAACGGCAGACTTGCCCGCTGAATTGCCTCC
CTTGCTGCGCCGTTTATACGCCAGCCGGGGCGTGCGCAGTGCGCAAGAACTGGAACGCAG
TGTTAAAGGTATGTTGCCCTGGCAGCAACTGAGCGGCGTCGAAAAGGCCGTTGAGATCCT
TTACAACGCTTTTCGCGAAGGAACGCGGATTATTGTGGTCGGCGATTTTGACGCCGACGGC
GCGACCAGCACGGCTCTAAGCGTGCTGGCGATGCGCTCGCTTGGTTGCAGCAATATCGAC
TATCTGGTACCAAACCGTTTCGAAGACGGTTACGGCTTAAGCCCGGAAGTAGTCGATCAG
GCCCATGCCCGTGGCGCGCAGTTAATTGTCACGGTGGATAACGGTATTTCCTCCCATGCGG
GCGTTGAACACGCTCGCTCGTTGGGCATTCCGGTTATTGTTACCGATCACCATTTGCCGGG
CGAAACATTACCCGCAGCGGAAGCGATCATTAACCCTAACTTGCGCGACTGTAATTTCCCG
TCGAAATCACTGGCAGGCGTGGGTGTGGCGTTTTATCTGATGCTGGCGCTGCGCACCTTTT
TGCGCGATCAGGGCTGGTTTGATGAGCGTGGCATCGCAATTCCTAACCTGGCAGAACTGC
TGGATCTGGTCGCGCTGGGAACAGTGGCGGACGTCGTGCCGCTGGACGCTAATAATCGCA
TTCTGACCTGGCAGGGGATGAGTCGCATCCGTGCCGGAAAGTGCCGTCCAGGGATTAAAG
CGCTGCTGGAAGTGGCAAACCGTGATGCACAAAAACTCGCCGCCAGCGATTTAGGTTTTG
CGCTGGGGCCACGTCTCAATGCTGCCGGACGACTGGACGATATGTCCGTCGGTGTGGCGC
TCTTGCTGTGCGACAACATCGGCGAAGCGCGCGTGCTGGCAAATGAACTCGATGCGCTAA
ACCAGACGCGAAAAGAGATCGAACAAGGAATGCAAGTTGAAGCCCTGACCCTGTGCGAG
AAACTGGAGCGAAGTCGCGACACGCTACCCGGCGGGCTGGCAATGTATCACCCCGAATGG
CATCAGGGCGTTGTCGGTATTCTGGCTTCGCGCATCAAAGAGCGTTTTCACCGTCCGGTTA
TCGCCTTTGCGCCAGCAGGTGATGGTACGCTGAAAGGTTCAGGTCGCTCCATTCAGGGGC
TGCATATGCGTGATGCACTGGAGCGATTAGACACACTCTACCCTGGCATGATACTGAAGTT
TGGCGGTCATGCGATGGCGGCGGGTTTGTCGCTGGAAGAGGATAAATTCGAACTCTTTCA
ACAACGGTTTGGCGAGCTGGTTACCGAGTGGCTGGACCCTTCGCTATTGCAAGGCGAAGT
GGTGTCAGACGGCCCGTTAAGCCCGGCCGAAATGACCATGGAAGTGGCGCAGCTGCTGC
GCGATGCTGGCCCGTGGGGGCAGATGTTCCCGGAGCCGCTGTTTGATGGTCATTTCCGTCT
GCTGCAACAGCGGCTGGTGGGCGAACGTCATTTGAAAGTCATGGTCGAACCGGTCGGCG
GCGGTCCGCTGCTGGATGGTATTGCTTTTAATGTCGATACCGCCCTCTGGCCGGATAACGG
CGTGCGCGAAGTGCAACTGGCTTACAAGCTCGATATCAACGAGTTTCGCGGCAACCGCAG
CCTGCAAATTATCATCGACAATATCTGGCCAATTCTGCAGAAAAGGCCGGCGGCCACGAA
AAAGGCCGGCCAGGCAAAAAAGAAAAAGCACCACCACCACCACCACTGA

3) SpyCas9-GFP

ATGGGCAGCAGCCATCATCATCATCATCACAGCAGCGGCCTGGTGCCGCGCGGCAGCCAT
ATGGCTAGCATGACTGGTGGACAGCAAATGGGTCGCGGATCCGAATTCGAGCTCCGTCGA
CAAGCTTGCGGCCGCATGGACAAGAAGTACAGCATCGGCCTGGACATCGGTACCAACAGC
GTGGGCTGGGCCGTGATCACCGACGAGTACAAGGTGCCCAGCAAGAAGTTCAAGGTGCT
GGGCAACACCGACCGCCACAGCATCAAGAAGAACCTGATCGGCGCCCTGCTGTTCGACA
GCGGCGAGACCGCCGAGGCCACCCGCCTGAAGCGCACCGCCCGCCGCCGCTACACCCGC
CGCAAGAACCGCATCTGCTACCTGCAGGAGATCTTCAGCAACGAGATGGCCAAGGTGGAC
GACAGCTTCTTCCACCGCCTGGAGGAGAGCTTCCTGGTGGAGGAGGACAAGAAGCACGA
GCGCCACCCCATCTTCGGCAACATCGTGGACGAGGTGGCCTACCACGAGAAGTACCCCAC
CATCTACCACCTGCGCAAGAAGCTGGTGGACAGCACCGACAAGGCCGACCTGCGCCTGAT
CTACCTGGCCCTGGCCCACATGATCAAGTTCCGCGGCCACTTCCTGATCGAGGGCGACCT
GAACCCCGACAACAGCGACGTGGACAAGCTGTTCATCCAGCTGGTGCAGACCTACAACC

AGCTGTTCGAGGAGAACCCCATCAACGCCAGCGGCGTGGACGCCAAGGCCATCCTGAGC
GCCCGCCTGAGCAAGAGCCGCCGCCTGGAGAACCTGATCGCCCAGCTGCCCGGCGAGAA
GAAGAACGGCCTGTTCGGCAACCTGATCGCCCTGAGCCTGGGCCTGACCCCCAACTTCAA
GAGCAACTTCGACCTGGCCGAGGACGCCAAGCTGCAGCTGAGCAAGGACACCTACGACG
ACGACCTGGACAACCTGCTGGCCCAGATCGGCGACCAGTACGCCGACCTGTTCCTGGCCG
CCAAGAACCTGAGCGACGCCATCCTGCTGAGCGACATCCTGCGCGTGAACACCGAGATCA
CCAAGGCCCCCCTGAGCGCCAGCATGATCAAGCGCTACGACGAGCACCACCAGGACCTG
ACCCTGCTGAAGGCCCTGGTGCGCCAGCAGCTGCCCGAGAAGTACAAGGAGATCTTCTTC
GACCAGAGCAAGAACGGCTACGCCGGCTACATCGACGGCGGCGCCAGCCAGGAGGAGTT
CTACAAGTTCATCAAGCCCATCCTGGAGAAGATGGACGGCACCGAGGAGCTGCTGGTGAA
GCTGAACCGCGAGGACCTGCTGCGCAAGCAGCGCACCTTCGACAACGGCAGCATCCCCC
ACCAGATCCACCTGGGCGAGCTGCACGCCATCCTGCGCCGCCAGGAGGACTTCTACCCCT
TCCTGAAGGACAACCGCGAGAAGATCGAGAAGATCCTGACCTTCCGCATCCCCTACTACG
TGGGCCCCCTGGCCCGCGGCAACAGCCGCTTCGCCTGGATGACCCGCAAGAGCGAGGAG
ACCATCACCCCCTGGAACTTCGAGGAGGTGGTGGACAAGGGCGCCAGCGCCCAGAGCTT
CATCGAGCGCATGACCAACTTCGACAAGAACCTGCCCAACGAGAAGGTGCTGCCCAAGC
ACAGCCTGCTGTACGAGTACTTCACCGTGTACAACGAGCTGACCAAGGTGAAGTACGTGA
CCGAGGGCATGCGCAAGCCCGCCTTCCTGAGCGGCGAGCAGAAGAAGGCCATCGTGGAC
CTGCTGTTCAAGACCAACCGCAAGGTGACCGTGAAGCAGCTGAAGGAGGACTACTTCAA
GAAGATCGAGTGCTTCGACAGCGTGGAGATCAGCGGCGTGGAGGACCGCTTCAACGCCA
GCCTGGGCACCTACCACGACCTGCTGAAGATCATCAAGGACAAGGACTTCCTGGACAACG
AGGAGAACGAGGACATCCTGGAGGACATCGTGCTGACCCTGACCCTGTTCGAGGACCGC
GAGATGATCGAGGAGCGCCTGAAGACCTACGCCCACCTGTTCGACGACAAGGTGATGAA
GCAGCTGAAGCGCCGCCGCTACACCGGCTGGGGCCGCCTGAGCCGCAAGCTTATCAACG
GCATCCGCGACAAGCAGAGCGGCAAGACCATCCTGGACTTCCTGAAGAGCGACGGCTTC
GCCAACCGCAACTTCATGCAGCTGATCCACGACGACAGCCTGACCTTCAAGGAGGACATC
CAGAAGGCCCAGGTGAGCGGCCAGGGCGACAGCCTGCACGAGCACATCGCCAACCTGGC
CGGCAGCCCCGCCATCAAGAAGGGCATCCTGCAGACCGTGAAGGTGGTGGACGAGCTGG
TGAAGGTGATGGGCCGCCACAAGCCCGAGAACATCGTGATCGAGATGGCCCGCGAGAAC
CAGACCACCCAGAAGGGCCAGAAGAACAGCCGCGAGCGCATGAAGCGCATCGAGGAGG
GCATCAAGGAGCTGGGCAGCCAGATCCTGAAGGAGCACCCCGTGGAGAACACCCAGCTG
CAGAACGAGAAGCTGTACCTGTACTACCTGCAGAACGGCCGCGACATGTACGTGGACCAG
GAGCTGGACATCAACCGCCTGAGCGACTACGACGTGGACCACATCGTGCCCCAGAGCTTC
CTGAAGGACGACAGCATCGACAACAAGGTGCTGACCCGCAGCGACAAGAACCGCGGCA
AGAGCGACAACGTGCCCAGCGAGGAGGTGGTGAAGAAGATGAAGAACTACTGGCGCCA
GCTGCTGAACGCCAAGCTGATCACCCAGCGCAAGTTCGACAACCTGACCAAGGCCGAGC
GCGGCGGCCTGAGCGAGCTGGACAAGGCCGGCTTCATCAAGCGCCAGCTGGTGGAGACC
CGCCAGATCACCAAGCACGTGGCCCAGATCCTGGACAGCCGCATGAACACCAAGTACGA
CGAGAACGACAAGCTGATCCGCGAGGTGAAGGTGATCACCCTGAAGAGCAAGCTGGTGA
GCGACTTCCGCAAGGACTTCCAGTTCTACAAGGTGCGCGAGATCAACAACTACCACCACG
CCCACGACGCCTACCTGAACGCCGTGGTGGGCACCGCCCTGATCAAGAAGTACCCCAAGC
TGGAGAGCGAGTTCGTGTACGGCGACTACAAGGTGTACGACGTGCGCAAGATGATCGCCA
AGAGCGAGCAGGAGATCGGCAAGGCCACCGCCAAGTACTTCTTCTACAGCAACATCATGA
ACTTCTTCAAGACCGAGATCACCCTGGCCAACGGCGAGATCCGCAAGCGCCCCCTGATCG
AGACCAACGGCGAGACCGGCGAGATCGTGTGGGACAAGGGCCGCGACTTCGCCACCGTG
CGCAAGGTGCTGAGCATGCCCCAGGTGAACATCGTGAAGAAGACCGAGGTGCAGACCGG
CGGCTTCAGCAAGGAGAGCATCCTGCCCAAGCGCAACAGCGACAAGCTGATCGCCCGCA
AGAAGGACTGGGACCCCAAGAAGTACGGCGGCTTCGACAGCCCCACCGTGGCCTACAGC
GTGCTGGTGGTGGCCAAGGTGGAGAAGGGCAAGAGCAAGAAGCTGAAGAGCGTGAAGG
AGCTGCTGGGCATCACCATCATGGAGCGCAGCAGCTTCGAGAAGAACCCCATCGACTTCC

TGGAGGCCAAGGGCTACAAGGAGGTGAAGAAGGACCTGATCATCAAGCTGCCCAAGTAC
AGCCTGTTCGAGCTGGAGAACGGCCGCAAGCGCATGCTGGCCAGCGCCGGCGAGCTGCA
GAAGGGCAACGAGCTGGCCCTGCCCAGCAAGTACGTGAACTTCCTGTACCTGGCCAGCC
ACTACGAGAAGCTGAAGGGCAGCCCCGAGGACAACGAGCAGAAGCAGCTGTTCGTGGA
GCAGCACAAGCACTACCTGGACGAGATCATCGAGCAGATCAGCGAGTTCAGCAAGCGCG
TGATCCTGGCCGACGCCAACCTGGACAAGGTGCTGAGCGCCTACAACAAGCACCGCGAC
AAGCCCATCCGCGAGCAGGCCGAGAACATCATCCACCTGTTCACCCTGACCAACCTGGGC
GCCCCCGCCGCCTTCAAGTACTTCGACACCACCATCGACCGCAAGCGCTACACCAGCACC
AAGGAGGTGCTGGACGCCACCCTGATCCACCAGAGCATCACCGGTCTGTACGAGACCCGC
ATCGACCTGAGCCAGCTGGGCGGCGACGCGGCCGCACTCGACCTCGAGATGAGTAAAGG
AGAAGAACTTTTCACTGGAGTTGTCCCAATTCTTGTTGAATTAGATGGTGATGTTAATGGG
CACAAATTTTCTGTCAGTGGAGAGGGTGAAGGTGATGCAACATACGGAAAACTTACCCTT
AAATTTATTTGCACTACTGGAAAACTACCTGTTCCATGGCCAACACTTGTCACTACTTTCTC
TTATGGTGTTCAATGCTTTTCAAGATACCCAGATCATATGAAGCGGCACGACTTCTTCAAG
AGCGCCATGCCTGAGGGATACGTGCAGGAGAGGACCATCTCTTTCAAGGACGACGGGAA
CTACAAGACACGTGCTGAAGTCAAGTTTGAGGGAGACACCCTCGTCAACAGGATCGAGC
TTAAGGGAATCGATTTCAAGGAGGACGGAAACATCCTCGGCCACAAGTTGGAATACAACT
ACAACTCCCACAACGTATACATCACGGCAGACAAACAAAAGAATGGAATCAAAGCTAACT
TCAAAATTAGACACAACATTGAAGATGGAAGCGTTCAACTAGCAGACCATTATCAACAAA
ATACTCCAATTGGCGATGGCCCTGTCCTTTTACCAGACAACCATTACCTGTCCACACAATCT
GCCCTTTCGAAAGATCCCAACGAAAAGAGAGACCACATGGTCCTTCTTGAGTTTGTAACA
GCTGCTGGGATTACACATGGCATGGATGAACTATACAAACTCGAGAAAGGCCGGCGGCC
ACGAAAAAGGCCGGCCAGGCAAAAAAGAAAAAGCACCACCACCACCACCACTGA

4) SpyCas9-RecE

ATGGGCAGCAGCCATCATCATCATCATCACAGCAGCGGCCTGGTGCCGCGCGGCAGCCAT
ATGGCTAGCATGACTGGTGGACAGCAAATGGGTCGCGGATCCGAATTCGAGCTCCGTCGA
CAAGCTTGCGGCCGCATGGACAAGAAGTACAGCATCGGCCTGGACATCGGTACCAACAGC
GTGGGCTGGGCCGTGATCACCGACGAGTACAAGGTGCCCAGCAAGAAGTTCAAGGTGCT
GGGCAACACCGACCGCCACAGCATCAAGAAGAACCTGATCGGCGCCCTGCTGTTCGACA
GCGGCGAGACCGCCGAGGCCACCCGCCTGAAGCGCACCGCCCGCCGCCGCTACACCCGC
CGCAAGAACCGCATCTGCTACCTGCAGGAGATCTTCAGCAACGAGATGGCCAAGGTGGAC
GACAGCTTCTTCCACCGCCTGGAGGAGAGCTTCCTGGTGGAGGAGGACAAGAAGCACGA
GCGCCACCCCATCTTCGGCAACATCGTGGACGAGGTGGCCTACCACGAGAAGTACCCCAC
CATCTACCACCTGCGCAAGAAGCTGGTGGACAGCACCGACAAGGCCGACCTGCGCCTGAT
CTACCTGGCCCTGGCCCACATGATCAAGTTCCGCGGCCACTTCCTGATCGAGGGCGACCT
GAACCCCGACAACAGCGACGTGGACAAGCTGTTCATCCAGCTGGTGCAGACCTACAACC
AGCTGTTCGAGGAGAACCCCATCAACGCCAGCGGCGTGGACGCCAAGGCCATCCTGAGC
GCCCGCCTGAGCAAGAGCCGCCGCCTGGAGAACCTGATCGCCCAGCTGCCCGGCGAGAA
GAAGAACGGCCTGTTCGGCAACCTGATCGCCCTGAGCCTGGGCCTGACCCCCAACTTCAA
GAGCAACTTCGACCTGGCCGAGGACGCCAAGCTGCAGCTGAGCAAGGACACCTACGACG
ACGACCTGGACAACCTGCTGGCCCAGATCGGCGACCAGTACGCCGACCTGTTCCTGGCCG
CCAAGAACCTGAGCGACGCCATCCTGCTGAGCGACATCCTGCGCGTGAACACCGAGATCA
CCAAGGCCCCCCTGAGCGCCAGCATGATCAAGCGCTACGACGAGCACCACCAGGACCTG
ACCCTGCTGAAGGCCCTGGTGCGCCAGCAGCTGCCCGAGAAGTACAAGGAGATCTTCTTC
GACCAGAGCAAGAACGGCTACGCCGGCTACATCGACGGCGGCGCCAGCCAGGAGGAGTT
CTACAAGTTCATCAAGCCCATCCTGGAGAAGATGGACGGCACCGAGGAGCTGCTGGTGAA
GCTGAACCGCGAGGACCTGCTGCGCAAGCAGCGCACCTTCGACAACGGCAGCATCCCCC

ACCAGATCCACCTGGGCGAGCTGCACGCCATCCTGCGCCGCCAGGAGGACTTCTACCCCT
TCCTGAAGGACAACCGCGAGAAGATCGAGAAGATCCTGACCTTCCGCATCCCCTACTACG
TGGGCCCCCTGGCCCGCGGCAACAGCCGCTTCGCCTGGATGACCCGCAAGAGCGAGGAG
ACCATCACCCCCTGGAACTTCGAGGAGGTGGTGGACAAGGGCGCCAGCGCCCAGAGCTT
CATCGAGCGCATGACCAACTTCGACAAGAACCTGCCCAACGAGAAGGTGCTGCCCAAGC
ACAGCCTGCTGTACGAGTACTTCACCGTGTACAACGAGCTGACCAAGGTGAAGTACGTGA
CCGAGGGCATGCGCAAGCCCGCCTTCCTGAGCGGCGAGCAGAAGAAGGCCATCGTGGAC
CTGCTGTTCAAGACCAACCGCAAGGTGACCGTGAAGCAGCTGAAGGAGGACTACTTCAA
GAAGATCGAGTGCTTCGACAGCGTGGAGATCAGCGGCGTGGAGGACCGCTTCAACGCCA
GCCTGGGCACCTACCACGACCTGCTGAAGATCATCAAGGACAAGGACTTCCTGGACAACG
AGGAGAACGAGGACATCCTGGAGGACATCGTGCTGACCCTGACCCTGTTCGAGGACCGC
GAGATGATCGAGGAGCGCCTGAAGACCTACGCCCACCTGTTCGACGACAAGGTGATGAA
GCAGCTGAAGCGCCGCCGCTACACCGGCTGGGGCCGCCTGAGCCGCAAGCTTATCAACG
GCATCCGCGACAAGCAGAGCGGCAAGACCATCCTGGACTTCCTGAAGAGCGACGGCTTC
GCCAACCGCAACTTCATGCAGCTGATCCACGACGACAGCCTGACCTTCAAGGAGGACATC
CAGAAGGCCCAGGTGAGCGGCCAGGGCGACAGCCTGCACGAGCACATCGCCAACCTGGC
CGGCAGCCCCGCCATCAAGAAGGGCATCCTGCAGACCGTGAAGGTGGTGGACGAGCTGG
TGAAGGTGATGGGCCGCCACAAGCCCGAGAACATCGTGATCGAGATGGCCCGCGAGAAC
CAGACCACCCAGAAGGGCCAGAAGAACAGCCGCGAGCGCATGAAGCGCATCGAGGAGG
GCATCAAGGAGCTGGGCAGCCAGATCCTGAAGGAGCACCCCGTGGAGAACACCCAGCTG
CAGAACGAGAAGCTGTACCTGTACTACCTGCAGAACGGCCGCGACATGTACGTGGACCAG
GAGCTGGACATCAACCGCCTGAGCGACTACGACGTGGACCACATCGTGCCCCAGAGCTTC
CTGAAGGACGACAGCATCGACAACAAGGTGCTGACCCGCAGCGACAAGAACCGCGGCA
AGAGCGACAACGTGCCCAGCGAGGAGGTGGTGAAGAAGATGAAGAACTACTGGCGCCA
GCTGCTGAACGCCAAGCTGATCACCCAGCGCAAGTTCGACAACCTGACCAAGGCCGAGC
GCGGCGGCCTGAGCGAGCTGGACAAGGCCGGCTTCATCAAGCGCCAGCTGGTGGAGACC
CGCCAGATCACCAAGCACGTGGCCCAGATCCTGGACAGCCGCATGAACACCAAGTACGA
CGAGAACGACAAGCTGATCCGCGAGGTGAAGGTGATCACCCTGAAGAGCAAGCTGGTGA
GCGACTTCCGCAAGGACTTCCAGTTCTACAAGGTGCGCGAGATCAACAACTACCACCACG
CCCACGACGCCTACCTGAACGCCGTGGTGGGCACCGCCCTGATCAAGAAGTACCCCAAGC
TGGAGAGCGAGTTCGTGTACGGCGACTACAAGGTGTACGACGTGCGCAAGATGATCGCCA
AGAGCGAGCAGGAGATCGGCAAGGCCACCGCCAAGTACTTCTTCTACAGCAACATCATGA
ACTTCTTCAAGACCGAGATCACCCTGGCCAACGGCGAGATCCGCAAGCGCCCCCTGATCG
AGACCAACGGCGAGACCGGCGAGATCGTGTGGGACAAGGGCCGCGACTTCGCCACCGTG
CGCAAGGTGCTGAGCATGCCCCAGGTGAACATCGTGAAGAAGACCGAGGTGCAGACCGG
CGGCTTCAGCAAGGAGAGCATCCTGCCCAAGCGCAACAGCGACAAGCTGATCGCCCGCA
AGAAGGACTGGGACCCCAAGAAGTACGGCGGCTTCGACAGCCCCACCGTGGCCTACAGC
GTGCTGGTGGTGGCCAAGGTGGAGAAGGGCAAGAGCAAGAAGCTGAAGAGCGTGAAGG
AGCTGCTGGGCATCACCATCATGGAGCGCAGCAGCTTCGAGAAGAACCCCATCGACTTCC
TGGAGGCCAAGGGCTACAAGGAGGTGAAGAAGGACCTGATCATCAAGCTGCCCAAGTAC
AGCCTGTTCGAGCTGGAGAACGGCCGCAAGCGCATGCTGGCCAGCGCCGGCGAGCTGCA
GAAGGGCAACGAGCTGGCCCTGCCCAGCAAGTACGTGAACTTCCTGTACCTGGCCAGCC
ACTACGAGAAGCTGAAGGGCAGCCCCGAGGACAACGAGCAGAAGCAGCTGTTCGTGGA
GCAGCACAAGCACTACCTGGACGAGATCATCGAGCAGATCAGCGAGTTCAGCAAGCGCG
TGATCCTGGCCGACGCCAACCTGGACAAGGTGCTGAGCGCCTACAACAAGCACCGCGAC
AAGCCCATCCGCGAGCAGGCCGAGAACATCATCCACCTGTTCACCCTGACCAACCTGGGC
GCCCCCGCCGCCTTCAAGTACTTCGACACCACCATCGACCGCAAGCGCTACACCAGCACC
AAGGAGGTGCTGGACGCCACCCTGATCCACCAGAGCATCACCGGTCTGTACGAGACCCGC
ATCGACCTGAGCCAGCTGGGCGGCGACGCGGCCGCACTCGACCTGCAGATGAGCACAAA
ACCACTCTTCCTGTTACGGAAAGCGAAAAAATCATCCGGTGAACCTGACGTCGTCCTGTG

GGCAAGCAACGATTTTGAATCGACCTGTGCCACTCTGGACTACCTGATCGTTAAGTCAGGT
AAAAAACTGAGCAGCTATTTTAAAGCTGTTGCCACGAATTTTCCTGTCGTTAATGACCTGC
CCGCTGAAGGTGAGATCGATTTTACCTGGAGTGAACGCTATCAACTCAGCAAAGACTCCA
TGACATGGGAACTAAAACCGGGAGCAGCACCAGACAACGCTCACTATCAAGGCAATACC
AACGTCAACGGCGAAGACATGACTGAGATTGAGGAGAATATGCTACTCCCAATTTCTGGC
CAGGAACTGCCCATTCGTTGGCTTGCTCAACACGGCAGCGAAAAACCGGTAACGCACGTT
TCACGCGACGGACTCCAGGCATTACACATTGCTCGGGCTGAAGAACTACCGGCTGTTACT
GCCCTGGCTGTTTCCCACAAAACCAGCCTGCTCGACCCGCTGGAAATTCGCGAACTCCAC
AAACTGGTTCGTGACACTGACAAAGTTTTCCCTAATCCTGGTAATTCAAACCTGGGACTGA
TAACTGCTTTTTTCGAAGCATACCTGAACGCTGACTACACCGATCGAGGACTGCTGACAA
AAGAGTGGATGAAGGGTAATCGTGTTTCACACATCACTCGCACGGCTTCCGGTGCTAATGC
TGGCGGCGGAAACCTCACCGATCGCGGCGAAGGTTTCGTACACGATCTGACGTCACTGGC
GCGCGACGTAGCCACTGGCGTACTGGCCCGTTCAATGGATCTGGACATCTATAACCTTCAT
CCGGCACACGCTAAACGCATTGAGGAAATTATCGCTGAAAATAAACCGCCCTTTTCTGTTT
TCCGCGACAAATTCATCACCATGCCTGGCGGGCTGGATTATTCCCGCGCCATCGTGGTTGC
GTCCGTAAAAGAAGCACCAATTGGGATCGAGGTCATCCCCGCGCACGTCACTGAATATCT
GAACAAAGTACTGACTGAAACCGATCATGCCAACCCTGATCCGGAAATCGTGGATATTGCC
TGCGGTCGCTCCTCTGCCCCGATGCCGCAGCGAGTAACAGAAGAAGGAAAACAGGATGA
TGAAGAAAAACCGCAACCATCTGGAACAACGGCAGTTGAACAGGGAGAGGCTGAAACA
ATGGAACCGGACGCAACTGAACATCATCAGGACACGCAGCCGCTGGATGCTCAGTCACAG
GTAAATTCTGTTGATGCGAAATATCAGGAACTGCGGGCAGAACTCCATGAAGCCCGGAAA
AACATTCCATCAAAAAATCCTGTCGATGACGATAAATTGCTTGCTGCATCACGTGGTGAAT
TTGTTGACGGAATTAGCGACCCGAACGATCCGAAATGGGTAAAGGGGATCCAGACTCGCG
ATTGTGTGTACCAGAACCAGCCAGAAACGGAAAAAACCAGCCCAGATATGAATCAACCTG
AGCCAGTAGTGCAACAGGAACCGGAAATAGCCTGCAATGCCTGCGGCCAGACTGGCGGG
GATAACTGCCCTGACTGTGGTGCGGTGATGGGCGACGCAACATACCAGGAAACATTCGAT
GAAGAGAGTCAGGTTGAAGCTAAGGAAAATGATCCGGAGGAAATGGAAGGCGCTGAACA
TCCGCACAATGAGAATGCTGGCAGCGATCCGCATCGCGATTGCAGTGATGAAACTGGCGA
AGTCGCAGATCCCGTAATCGTAGAAGACATAGAGCCAGGTATTTATTACGGAATTTCGAAT
GAGAATTACCACGCGGGTCCCGGTATCAGTAAGTCTCAGCTCGATGACATTGCTGATACTC
CGGCACTATATTTGTGGCGTAAAAATGCCCCCGTGGACACCACAAAGACAAAAACGCTCG
ATTTAGGAACTGCTTTCCACTGCCGGGTACTTGAACCGGAAGAATTCAGTAACCGCTTTAT
CGTAGCACCTGAATTTAACCGCCGTACAAACGCCGGAAAAGAAGAAGAGAAAGCGTTTC
TGATGGAATGCGCAAGCACAGGAAAAACGGTTATCACTGCGGAAGAAGGCCGGAAAATT
GAACTCATGTATCAAAGCGTTATGGCTTTGCCGCTGGGGCAATGGCTTGTTGAAAGCGCCG
GACACGCTGAATCATCAATTTACTGGGAAGATCCTGAAACAGGAATTTTGTGTCGGTGCCG
TCCGGACAAAATTATCCCTGAATTTCACTGGATCATGGACGTGAAAACTACGGCGGATATT
CAACGATTCAAAACCGCTTATTACGACTACCGCTATCACGTTCAGGATGCATTCTACAGTG
ACGGTTATGAAGCACAGTTTGGAGTGCAGCCAACTTTCGTTTTTCTGGTTGCCAGCACAA
CTATTGAATGCGGACGTTATCCGGTTGAAATTTTCATGATGGGCGAAGAAGCAAAACTGGC
AGGTCAACAGGAATATCACCGCAATCTGCGAACCCTGTCTGACTGCCTGAATACCGATGA
ATGGCCAGCTATTAAGACATTATCACTGCCCCGCTGGGCTAAGGAATATGCAAATGACCTG
CAGAAAAGGCCGGCGGCCACGAAAAAGGCCGGCCAGGCAAAAAAGAAAAAGCACCACC
ACCACCACCACTGA

5) SpyCas9-T5

ATGGGCAGCAGCCATCATCATCATCATCACAGCAGCGGCCTGGTGCCGCGCGGCAGCCAT
ATGGCTAGCATGACTGGTGGACAGCAAATGGGTCGCGGATCCGAATTCGAGCTCCGTCGA

CAAGCTTGCGGCCGCATGGACAAGAAGTACAGCATCGGCCTGGACATCGGTACCAACAGC
GTGGGCTGGGCCGTGATCACCGACGAGTACAAGGTGCCCAGCAAGAAGTTCAAGGTGCT
GGGCAACACCGACCGCCACAGCATCAAGAAGAACCTGATCGGCGCCCTGCTGTTCGACA
GCGGCGAGACCGCCGAGGCCACCCGCCTGAAGCGCACCGCCCGCCGCCGCTACACCCGC
CGCAAGAACCGCATCTGCTACCTGCAGGAGATCTTCAGCAACGAGATGGCCAAGGTGGAC
GACAGCTTCTTCCACCGCCTGGAGGAGAGCTTCCTGGTGGAGGAGGACAAGAAGCACGA
GCGCCACCCCATCTTCGGCAACATCGTGGACGAGGTGGCCTACCACGAGAAGTACCCCAC
CATCTACCACCTGCGCAAGAAGCTGGTGGACAGCACCGACAAGGCCGACCTGCGCCTGAT
CTACCTGGCCCTGGCCCACATGATCAAGTTCCGCGGCCACTTCCTGATCGAGGGCGACCT
GAACCCCGACAACAGCGACGTGGACAAGCTGTTCATCCAGCTGGTGCAGACCTACAACC
AGCTGTTCGAGGAGAACCCCATCAACGCCAGCGGCGTGGACGCCAAGGCCATCCTGAGC
GCCCGCCTGAGCAAGAGCCGCCGCCTGGAGAACCTGATCGCCCAGCTGCCCGGCGAGAA
GAAGAACGGCCTGTTCGGCAACCTGATCGCCCTGAGCCTGGGCCTGACCCCCAACTTCAA
GAGCAACTTCGACCTGGCCGAGGACGCCAAGCTGCAGCTGAGCAAGGACACCTACGACG
ACGACCTGGACAACCTGCTGGCCCAGATCGGCGACCAGTACGCCGACCTGTTCCTGGCCG
CCAAGAACCTGAGCGACGCCATCCTGCTGAGCGACATCCTGCGCGTGAACACCGAGATCA
CCAAGGCCCCCCTGAGCGCCAGCATGATCAAGCGCTACGACGAGCACCACCAGGACCTG
ACCCTGCTGAAGGCCCTGGTGCGCCAGCAGCTGCCCGAGAAGTACAAGGAGATCTTCTTC
GACCAGAGCAAGAACGGCTACGCCGGCTACATCGACGGCGGCGCCAGCCAGGAGGAGTT
CTACAAGTTCATCAAGCCCATCCTGGAGAAGATGGACGGCACCGAGGAGCTGCTGGTGAA
GCTGAACCGCGAGGACCTGCTGCGCAAGCAGCGCACCTTCGACAACGGCAGCATCCCCC
ACCAGATCCACCTGGGCGAGCTGCACGCCATCCTGCGCCGCCAGGAGGACTTCTACCCCT
TCCTGAAGGACAACCGCGAGAAGATCGAGAAGATCCTGACCTTCCGCATCCCCTACTACG
TGGGCCCCCTGGCCCGCGGCAACAGCCGCTTCGCCTGGATGACCCGCAAGAGCGAGGAG
ACCATCACCCCCTGGAACTTCGAGGAGGTGGTGGACAAGGGCGCCAGCGCCCAGAGCTT
CATCGAGCGCATGACCAACTTCGACAAGAACCTGCCCAACGAGAAGGTGCTGCCCAAGC
ACAGCCTGCTGTACGAGTACTTCACCGTGTACAACGAGCTGACCAAGGTGAAGTACGTGA
CCGAGGGCATGCGCAAGCCCGCCTTCCTGAGCGGCGAGCAGAAGAAGGCCATCGTGGAC
CTGCTGTTCAAGACCAACCGCAAGGTGACCGTGAAGCAGCTGAAGGAGGACTACTTCAA
GAAGATCGAGTGCTTCGACAGCGTGGAGATCAGCGGCGTGGAGGACCGCTTCAACGCCA
GCCTGGGCACCTACCACGACCTGCTGAAGATCATCAAGGACAAGGACTTCCTGGACAACG
AGGAGAACGAGGACATCCTGGAGGACATCGTGCTGACCCTGACCCTGTTCGAGGACCGC
GAGATGATCGAGGAGCGCCTGAAGACCTACGCCCACCTGTTCGACGACAAGGTGATGAA
GCAGCTGAAGCGCCGCCGCTACACCGGCTGGGGCCGCCTGAGCCGCAAGCTTATCAACG
GCATCCGCGACAAGCAGAGCGGCAAGACCATCCTGGACTTCCTGAAGAGCGACGGCTTC
GCCAACCGCAACTTCATGCAGCTGATCCACGACGACAGCCTGACCTTCAAGGAGGACATC
CAGAAGGCCCAGGTGAGCGGCCAGGGCGACAGCCTGCACGAGCACATCGCCAACCTGGC
CGGCAGCCCCGCCATCAAGAAGGGCATCCTGCAGACCGTGAAGGTGGTGGACGAGCTGG
TGAAGGTGATGGGCCGCCACAAGCCCGAGAACATCGTGATCGAGATGGCCCGCGAGAAC
CAGACCACCCAGAAGGGCCAGAAGAACAGCCGCGAGCGCATGAAGCGCATCGAGGAGG
GCATCAAGGAGCTGGGCAGCCAGATCCTGAAGGAGCACCCCGTGGAGAACACCCAGCTG
CAGAACGAGAAGCTGTACCTGTACTACCTGCAGAACGGCCGCGACATGTACGTGGACCAG
GAGCTGGACATCAACCGCCTGAGCGACTACGACGTGGACCACATCGTGCCCCAGAGCTTC
CTGAAGGACGACAGCATCGACAACAAGGTGCTGACCCGCAGCGACAAGAACCGCGGCA
AGAGCGACAACGTGCCCAGCGAGGAGGTGGTGAAGAAGATGAAGAACTACTGGCGCCA
GCTGCTGAACGCCAAGCTGATCACCCAGCGCAAGTTCGACAACCTGACCAAGGCCGAGC
GCGGCGGCCTGAGCGAGCTGGACAAGGCCGGCTTCATCAAGCGCCAGCTGGTGGAGACC
CGCCAGATCACCAAGCACGTGGCCCAGATCCTGGACAGCCGCATGAACACCAAGTACGA
CGAGAACGACAAGCTGATCCGCGAGGTGAAGGTGATCACCCTGAAGAGCAAGCTGGTGA
GCGACTTCCGCAAGGACTTCCAGTTCTACAAGGTGCGCGAGATCAACAACTACCACCACG

CCCACGACGCCTACCTGAACGCCGTGGTGGGCACCGCCCTGATCAAGAAGTACCCCAAGC
TGGAGAGCGAGTTCGTGTACGGCGACTACAAGGTGTACGACGTGCGCAAGATGATCGCCA
AGAGCGAGCAGGAGATCGGCAAGGCCACCGCCAAGTACTTCTTCTACAGCAACATCATGA
ACTTCTTCAAGACCGAGATCACCCTGGCCAACGGCGAGATCCGCAAGCGCCCCCTGATCG
AGACCAACGGCGAGACCGGCGAGATCGTGTGGGACAAGGGCCGCGACTTCGCCACCGTG
CGCAAGGTGCTGAGCATGCCCCAGGTGAACATCGTGAAGAAGACCGAGGTGCAGACCGG
CGGCTTCAGCAAGGAGAGCATCCTGCCCAAGCGCAACAGCGACAAGCTGATCGCCCGCA
AGAAGGACTGGGACCCCAAGAAGTACGGCGGCTTCGACAGCCCCACCGTGGCCTACAGC
GTGCTGGTGGTGGCCAAGGTGGAGAAGGGCAAGAGCAAGAAGCTGAAGAGCGTGAAGG
AGCTGCTGGGCATCACCATCATGGAGCGCAGCAGCTTCGAGAAGAACCCCATCGACTTCC
TGGAGGCCAAGGGCTACAAGGAGGTGAAGAAGGACCTGATCATCAAGCTGCCCAAGTAC
AGCCTGTTCGAGCTGGAGAACGGCCGCAAGCGCATGCTGGCCAGCGCCGGCGAGCTGCA
GAAGGGCAACGAGCTGGCCCTGCCCAGCAAGTACGTGAACTTCCTGTACCTGGCCAGCC
ACTACGAGAAGCTGAAGGGCAGCCCCGAGGACAACGAGCAGAAGCAGCTGTTCGTGGA
GCAGCACAAGCACTACCTGGACGAGATCATCGAGCAGATCAGCGAGTTCAGCAAGCGCG
TGATCCTGGCCGACGCCAACCTGGACAAGGTGCTGAGCGCCTACAACAAGCACCGCGAC
AAGCCCATCCGCGAGCAGGCCGAGAACATCATCCACCTGTTCACCCTGACCAACCTGGGC
GCCCCCGCCGCCTTCAAGTACTTCGACACCACCATCGACCGCAAGCGCTACACCAGCACC
AAGGAGGTGCTGGACGCCACCCTGATCCACCAGAGCATCACCGGTCTGTACGAGACCCGC
ATCGACCTGAGCCAGCTGGGCGGCGACGCGGCCGCACTCGACCTGCAGATGGCTTCCCGT
CGTAATCTAATGATTGTCGATGGAACTAACTTAGGCTTTCGCTTCAAACATAACAATAGTAA
AAAACCATTTGCCTCAAGTTATGTTTCAACTATTCAATCTCTGGCAAAATCCTACTCTGCCA
GAACTACGATTGTTCTAGGTGATAAGGGAAAATCTGTATTTCGTCTAGAACATCTACCAGA
GTATAAAGGTAATCGTGATGAAAAGTACGCACAACGTACGGAAGAGGAGAAAGCGCTAG
ATGAGCAGTTCTTTGAGTATTTGAAGGATGCTTTCGAGTTGTGTAAAACTACATTCCCAAC
TTTTACCATTCGTGGTGTAGAAGCAGACGATATGGCAGCTTATATTGTTAAGCTCATCGGGC
ATCTTTATGATCACGTTTGGCTAATATCTACAGATGGTGACTGGGATACTTTATTAACGGATA
AAGTTTCTCGTTTTTCTTTCACAACACGTCGTGAGTATCATCTTCGTGATATGTATGAACAT
CATAATGTTGATGATGTTGAGCAGTTTATCTCCCTGAAAGCAATTATGGGAGATCTAGGAGA
TAATATTCGTGGTGTTGAAGGAATAGGAGCAAAACGCGGATATAATATTATTCGTGAGTTTG
GTAACGTACTGGATATTATTGATCAGCTTCCACTGCCTGGAAAGCAGAAATATATACAGAAC
CTGAATGCATCGGAAGAACTGCTTTTCCGAAACTTGATTCTGGTTGATTTACCTACCTACTG
TGTGGATGCTATTGCTGCTGTAGGTCAAGATGTGTTAGATAAGTTTACAAAAGATATTTGG
AGATTGCAGAACAACTGCAGAAAAGGCCGGCGGCCACGAAAAAGGCCGGCCAGGCAAA
AAAGAAAAAGCACCACCACCACCACCACTGA

6) SpyCas9-lambda

ATGGGCAGCAGCCATCATCATCATCATCACAGCAGCGGCCTGGTGCCGCGCGGCAGCCAT
ATGGCTAGCATGACTGGTGGACAGCAAATGGGTCGCGGATCCGAATTCGAGCTCCGTCGA
CAAGCTTGCGGCCGCATGGACAAGAAGTACAGCATCGGCCTGGACATCGGTACCAACAGC
GTGGGCTGGGCCGTGATCACCGACGAGTACAAGGTGCCCAGCAAGAAGTTCAAGGTGCT
GGGCAACACCGACCGCCACAGCATCAAGAAGAACCTGATCGGCGCCCTGCTGTTCGACA
GCGGCGAGACCGCCGAGGCCACCCGCCTGAAGCGCACCGCCCGCCGCCGCTACACCCGC
CGCAAGAACCGCATCTGCTACCTGCAGGAGATCTTCAGCAACGAGATGGCCAAGGTGGAC
GACAGCTTCTTCCACCGCCTGGAGGAGAGCTTCCTGGTGGAGGAGGACAAGAAGCACGA
GCGCCACCCCATCTTCGGCAACATCGTGGACGAGGTGGCCTACCACGAGAAGTACCCCAC
CATCTACCACCTGCGCAAGAAGCTGGTGGACAGCACCGACAAGGCCGACCTGCGCCTGAT
CTACCTGGCCCTGGCCCACATGATCAAGTTCCGCGGCCACTTCCTGATCGAGGGCGACCT

GAACCCCGACAACAGCGACGTGGACAAGCTGTTCATCCAGCTGGTGCAGACCTACAACC
AGCTGTTCGAGGAGAACCCCATCAACGCCAGCGGCGTGGACGCCAAGGCCATCCTGAGC
GCCCGCCTGAGCAAGAGCCGCCGCCTGGAGAACCTGATCGCCCAGCTGCCCGGCGAGAA
GAAGAACGGCCTGTTCGGCAACCTGATCGCCCTGAGCCTGGGCCTGACCCCCAACTTCAA
GAGCAACTTCGACCTGGCCGAGGACGCCAAGCTGCAGCTGAGCAAGGACACCTACGACG
ACGACCTGGACAACCTGCTGGCCCAGATCGGCGACCAGTACGCCGACCTGTTCCTGGCCG
CCAAGAACCTGAGCGACGCCATCCTGCTGAGCGACATCCTGCGCGTGAACACCGAGATCA
CCAAGGCCCCCCTGAGCGCCAGCATGATCAAGCGCTACGACGAGCACCACCAGGACCTG
ACCCTGCTGAAGGCCCTGGTGCGCCAGCAGCTGCCCGAGAAGTACAAGGAGATCTTCTTC
GACCAGAGCAAGAACGGCTACGCCGGCTACATCGACGGCGGCGCCAGCCAGGAGGAGTT
CTACAAGTTCATCAAGCCCATCCTGGAGAAGATGGACGGCACCGAGGAGCTGCTGGTGAA
GCTGAACCGCGAGGACCTGCTGCGCAAGCAGCGCACCTTCGACAACGGCAGCATCCCCC
ACCAGATCCACCTGGGCGAGCTGCACGCCATCCTGCGCCGCCAGGAGGACTTCTACCCCT
TCCTGAAGGACAACCGCGAGAAGATCGAGAAGATCCTGACCTTCCGCATCCCCTACTACG
TGGGCCCCCTGGCCCGCGGCAACAGCCGCTTCGCCTGGATGACCCGCAAGAGCGAGGAG
ACCATCACCCCCTGGAACTTCGAGGAGGTGGTGGACAAGGGCGCCAGCGCCCAGAGCTT
CATCGAGCGCATGACCAACTTCGACAAGAACCTGCCCAACGAGAAGGTGCTGCCCAAGC
ACAGCCTGCTGTACGAGTACTTCACCGTGTACAACGAGCTGACCAAGGTGAAGTACGTGA
CCGAGGGCATGCGCAAGCCCGCCTTCCTGAGCGGCGAGCAGAAGAAGGCCATCGTGGAC
CTGCTGTTCAAGACCAACCGCAAGGTGACCGTGAAGCAGCTGAAGGAGGACTACTTCAA
GAAGATCGAGTGCTTCGACAGCGTGGAGATCAGCGGCGTGGAGGACCGCTTCAACGCCA
GCCTGGGCACCTACCACGACCTGCTGAAGATCATCAAGGACAAGGACTTCCTGGACAACG
AGGAGAACGAGGACATCCTGGAGGACATCGTGCTGACCCTGACCCTGTTCGAGGACCGC
GAGATGATCGAGGAGCGCCTGAAGACCTACGCCCACCTGTTCGACGACAAGGTGATGAA
GCAGCTGAAGCGCCGCCGCTACACCGGCTGGGGCCGCCTGAGCCGCAAGCTTATCAACG
GCATCCGCGACAAGCAGAGCGGCAAGACCATCCTGGACTTCCTGAAGAGCGACGGCTTC
GCCAACCGCAACTTCATGCAGCTGATCCACGACGACAGCCTGACCTTCAAGGAGGACATC
CAGAAGGCCCAGGTGAGCGGCCAGGGCGACAGCCTGCACGAGCACATCGCCAACCTGGC
CGGCAGCCCCGCCATCAAGAAGGGCATCCTGCAGACCGTGAAGGTGGTGGACGAGCTGG
TGAAGGTGATGGGCCGCCACAAGCCCGAGAACATCGTGATCGAGATGGCCCGCGAGAAC
CAGACCACCCAGAAGGGCCAGAAGAACAGCCGCGAGCGCATGAAGCGCATCGAGGAGG
GCATCAAGGAGCTGGGCAGCCAGATCCTGAAGGAGCACCCCGTGGAGAACACCCAGCTG
CAGAACGAGAAGCTGTACCTGTACTACCTGCAGAACGGCCGCGACATGTACGTGGACCAG
GAGCTGGACATCAACCGCCTGAGCGACTACGACGTGGACCACATCGTGCCCCAGAGCTTC
CTGAAGGACGACAGCATCGACAACAAGGTGCTGACCCGCAGCGACAAGAACCGCGGCA
AGAGCGACAACGTGCCCAGCGAGGAGGTGGTGAAGAAGATGAAGAACTACTGGCGCCA
GCTGCTGAACGCCAAGCTGATCACCCAGCGCAAGTTCGACAACCTGACCAAGGCCGAGC
GCGGCGGCCTGAGCGAGCTGGACAAGGCCGGCTTCATCAAGCGCCAGCTGGTGGAGACC
CGCCAGATCACCAAGCACGTGGCCCAGATCCTGGACAGCCGCATGAACACCAAGTACGA
CGAGAACGACAAGCTGATCCGCGAGGTGAAGGTGATCACCCTGAAGAGCAAGCTGGTGA
GCGACTTCCGCAAGGACTTCCAGTTCTACAAGGTGCGCGAGATCAACAACTACCACCACG
CCCACGACGCCTACCTGAACGCCGTGGTGGGCACCGCCCTGATCAAGAAGTACCCCAAGC
TGGAGAGCGAGTTCGTGTACGGCGACTACAAGGTGTACGACGTGCGCAAGATGATCGCCA
AGAGCGAGCAGGAGATCGGCAAGGCCACCGCCAAGTACTTCTTCTACAGCAACATCATGA
ACTTCTTCAAGACCGAGATCACCCTGGCCAACGGCGAGATCCGCAAGCGCCCCCTGATCG
AGACCAACGGCGAGACCGGCGAGATCGTGTGGGACAAGGGCCGCGACTTCGCCACCGTG
CGCAAGGTGCTGAGCATGCCCCAGGTGAACATCGTGAAGAAGACCGAGGTGCAGACCGG
CGGCTTCAGCAAGGAGAGCATCCTGCCCAAGCGCAACAGCGACAAGCTGATCGCCCGCA
AGAAGGACTGGGACCCCAAGAAGTACGGCGGCTTCGACAGCCCCACCGTGGCCTACAGC
GTGCTGGTGGTGGCCAAGGTGGAGAAGGGCAAGAGCAAGAAGCTGAAGAGCGTGAAGG

AGCTGCTGGGCATCACCATCATGGAGCGCAGCAGCTTCGAGAAGAACCCCATCGACTTCC
TGGAGGCCAAGGGCTACAAGGAGGTGAAGAAGGACCTGATCATCAAGCTGCCCAAGTAC
AGCCTGTTCGAGCTGGAGAACGGCCGCAAGCGCATGCTGGCCAGCGCCGGCGAGCTGCA
GAAGGGCAACGAGCTGGCCCTGCCCAGCAAGTACGTGAACTTCCTGTACCTGGCCAGCC
ACTACGAGAAGCTGAAGGGCAGCCCCGAGGACAACGAGCAGAAGCAGCTGTTCGTGGA
GCAGCACAAGCACTACCTGGACGAGATCATCGAGCAGATCAGCGAGTTCAGCAAGCGCG
TGATCCTGGCCGACGCCAACCTGGACAAGGTGCTGAGCGCCTACAACAAGCACCGCGAC
AAGCCCATCCGCGAGCAGGCCGAGAACATCATCCACCTGTTCACCCTGACCAACCTGGGC
GCCCCCGCCGCCTTCAAGTACTTCGACACCACCATCGACCGCAAGCGCTACACCAGCACC
AAGGAGGTGCTGGACGCCACCCTGATCCACCAGAGCATCACCGGTCTGTACGAGACCCGC
ATCGACCTGAGCCAGCTGGGCGGCGACGCGGCCGCACTCGACCTCGAGATGACACCGGA
CATTATCCTGCAGCGTACCGGGATCGATGTGAGAGCTGTCGAACAGGGGGATGATGCGTG
GCACAAATTACGGCTCGGCGTCATCACCGCTTCAGAAGTTCACAACGTGATAGCAAAACC
CCGCTCCGGAAAGAAGTGGCCTGACATGAAAATGTCCTACTTCCACACCCTGCTTGCTGA
GGTTTGCACCGGTGTGGCTCCGGAAGTTAACGCTAAAGCACTGGCCTGGGGAAAACAGT
ACGAGAACGACGCCAGAACCCTGTTTGAATTCACTTCCGGCGTGAATGTTACTGAATCCC
CGATCATCTATCGCGACGAAAGTATGCGTACCGCCTGCTCTCCCGATGGTTTATGCAGTGAC
GGCAACGGCCTTGAACTGAAATGCCCGTTTACCTCCCGGGATTTCATGAAGTTCCGGCTCG
GTGGTTTCGAGGCCATAAAGTCAGCTTACATGGCCCAGGTGCAGTACAGCATGTGGGTGA
CGCGAAAAAATGCCTGGTACTTTGCCAACTATGACCCGCGTATGAAGCGTGAAGGCCTGC
ATTATGTCGTGATTGAGCGGGATGAAAAGTACATGGCGAGTTTTGACGAGATCGTGCCGGA
GTTCATCGAAAAAATGGACGAGGCACTGGCTGAAATTGGTTTTGTATTTGGGGAGCAATG
GCGACTCGAGAAAGGCCGGCGGCCACGAAAAAGGCCGGCCAGGCAAAAAAGAAAAAG
CACCACCACCACCACCACTGA

7) SpyCas9-mungbean

ATGGGCAGCAGCCATCATCATCATCATCACAGCAGCGGCCTGGTGCCGCGCGGCAGCCAT
ATGGCTAGCATGACTGGTGGACAGCAAATGGGTCGCGGATCCGAATTCGAGCTCCGTCGA
CAAGCTTGCGGCCGCATGGACAAGAAGTACAGCATCGGCCTGGACATCGGTACCAACAGC
GTGGGCTGGGCCGTGATCACCGACGAGTACAAGGTGCCCAGCAAGAAGTTCAAGGTGCT
GGGCAACACCGACCGCCACAGCATCAAGAAGAACCTGATCGGCGCCCTGCTGTTCGACA
GCGGCGAGACCGCCGAGGCCACCCGCCTGAAGCGCACCGCCCGCCGCCGCTACACCCGC
CGCAAGAACCGCATCTGCTACCTGCAGGAGATCTTCAGCAACGAGATGGCCAAGGTGGAC
GACAGCTTCTTCCACCGCCTGGAGGAGAGCTTCCTGGTGGAGGAGGACAAGAAGCACGA
GCGCCACCCCATCTTCGGCAACATCGTGGACGAGGTGGCCTACCACGAGAAGTACCCCAC
CATCTACCACCTGCGCAAGAAGCTGGTGGACAGCACCGACAAGGCCGACCTGCGCCTGAT
CTACCTGGCCCTGGCCCACATGATCAAGTTCCGCGGCCACTTCCTGATCGAGGGCGACCT
GAACCCCGACAACAGCGACGTGGACAAGCTGTTCATCCAGCTGGTGCAGACCTACAACC
AGCTGTTCGAGGAGAACCCCATCAACGCCAGCGGCGTGGACGCCAAGGCCATCCTGAGC
GCCCGCCTGAGCAAGAGCCGCCGCCTGGAGAACCTGATCGCCCAGCTGCCCGGCGAGAA
GAAGAACGGCCTGTTCGGCAACCTGATCGCCCTGAGCCTGGGCCTGACCCCCAACTTCAA
GAGCAACTTCGACCTGGCCGAGGACGCCAAGCTGCAGCTGAGCAAGGACACCTACGACG
ACGACCTGGACAACCTGCTGGCCCAGATCGGCGACCAGTACGCCGACCTGTTCCTGGCCG
CCAAGAACCTGAGCGACGCCATCCTGCTGAGCGACATCCTGCGCGTGAACACCGAGATCA
CCAAGGCCCCCCTGAGCGCCAGCATGATCAAGCGCTACGACGAGCACCACCAGGACCTG
ACCCTGCTGAAGGCCCTGGTGCGCCAGCAGCTGCCCGAGAAGTACAAGGAGATCTTCTTC
GACCAGAGCAAGAACGGCTACGCCGGCTACATCGACGGCGGCGCCAGCCAGGAGGAGTT
CTACAAGTTCATCAAGCCCATCCTGGAGAAGATGGACGGCACCGAGGAGCTGCTGGTGAA

GCTGAACCGCGAGGACCTGCTGCGCAAGCAGCGCACCTTCGACAACGGCAGCATCCCCC
ACCAGATCCACCTGGGCGAGCTGCACGCCATCCTGCGCCGCCAGGAGGACTTCTACCCCT
TCCTGAAGGACAACCGCGAGAAGATCGAGAAGATCCTGACCTTCCGCATCCCCTACTACG
TGGGCCCCCTGGCCCGCGGCAACAGCCGCTTCGCCTGGATGACCCGCAAGAGCGAGGAG
ACCATCACCCCCTGGAACTTCGAGGAGGTGGTGGACAAGGGCGCCAGCGCCCAGAGCTT
CATCGAGCGCATGACCAACTTCGACAAGAACCTGCCCAACGAGAAGGTGCTGCCCAAGC
ACAGCCTGCTGTACGAGTACTTCACCGTGTACAACGAGCTGACCAAGGTGAAGTACGTGA
CCGAGGGCATGCGCAAGCCCGCCTTCCTGAGCGGCGAGCAGAAGAAGGCCATCGTGGAC
CTGCTGTTCAAGACCAACCGCAAGGTGACCGTGAAGCAGCTGAAGGAGGACTACTTCAA
GAAGATCGAGTGCTTCGACAGCGTGGAGATCAGCGGCGTGGAGGACCGCTTCAACGCCA
GCCTGGGCACCTACCACGACCTGCTGAAGATCATCAAGGACAAGGACTTCCTGGACAACG
AGGAGAACGAGGACATCCTGGAGGACATCGTGCTGACCCTGACCCTGTTCGAGGACCGC
GAGATGATCGAGGAGCGCCTGAAGACCTACGCCCACCTGTTCGACGACAAGGTGATGAA
GCAGCTGAAGCGCCGCCGCTACACCGGCTGGGGCCGCCTGAGCCGCAAGCTTATCAACG
GCATCCGCGACAAGCAGAGCGGCAAGACCATCCTGGACTTCCTGAAGAGCGACGGCTTC
GCCAACCGCAACTTCATGCAGCTGATCCACGACGACAGCCTGACCTTCAAGGAGGACATC
CAGAAGGCCCAGGTGAGCGGCCAGGGCGACAGCCTGCACGAGCACATCGCCAACCTGGC
CGGCAGCCCCGCCATCAAGAAGGGCATCCTGCAGACCGTGAAGGTGGTGGACGAGCTGG
TGAAGGTGATGGGCCGCCACAAGCCCGAGAACATCGTGATCGAGATGGCCCGCGAGAAC
CAGACCACCCAGAAGGGCCAGAAGAACAGCCGCGAGCGCATGAAGCGCATCGAGGAGG
GCATCAAGGAGCTGGGCAGCCAGATCCTGAAGGAGCACCCCGTGGAGAACACCCAGCTG
CAGAACGAGAAGCTGTACCTGTACTACCTGCAGAACGGCCGCGACATGTACGTGGACCAG
GAGCTGGACATCAACCGCCTGAGCGACTACGACGTGGACCACATCGTGCCCCAGAGCTTC
CTGAAGGACGACAGCATCGACAACAAGGTGCTGACCCGCAGCGACAAGAACCGCGGCA
AGAGCGACAACGTGCCCAGCGAGGAGGTGGTGAAGAAGATGAAGAACTACTGGCGCCA
GCTGCTGAACGCCAAGCTGATCACCCAGCGCAAGTTCGACAACCTGACCAAGGCCGAGC
GCGGCGGCCTGAGCGAGCTGGACAAGGCCGGCTTCATCAAGCGCCAGCTGGTGGAGACC
CGCCAGATCACCAAGCACGTGGCCCAGATCCTGGACAGCCGCATGAACACCAAGTACGA
CGAGAACGACAAGCTGATCCGCGAGGTGAAGGTGATCACCCTGAAGAGCAAGCTGGTGA
GCGACTTCCGCAAGGACTTCCAGTTCTACAAGGTGCGCGAGATCAACAACTACCACCACG
CCCACGACGCCTACCTGAACGCCGTGGTGGGCACCGCCCTGATCAAGAAGTACCCCAAGC
TGGAGAGCGAGTTCGTGTACGGCGACTACAAGGTGTACGACGTGCGCAAGATGATCGCCA
AGAGCGAGCAGGAGATCGGCAAGGCCACCGCCAAGTACTTCTTCTACAGCAACATCATGA
ACTTCTTCAAGACCGAGATCACCCTGGCCAACGGCGAGATCCGCAAGCGCCCCCTGATCG
AGACCAACGGCGAGACCGGCGAGATCGTGTGGGACAAGGGCCGCGACTTCGCCACCGTG
CGCAAGGTGCTGAGCATGCCCCAGGTGAACATCGTGAAGAAGACCGAGGTGCAGACCGG
CGGCTTCAGCAAGGAGAGCATCCTGCCCAAGCGCAACAGCGACAAGCTGATCGCCCGCA
AGAAGGACTGGGACCCCAAGAAGTACGGCGGCTTCGACAGCCCCACCGTGGCCTACAGC
GTGCTGGTGGTGGCCAAGGTGGAGAAGGGCAAGAGCAAGAAGCTGAAGAGCGTGAAGG
AGCTGCTGGGCATCACCATCATGGAGCGCAGCAGCTTCGAGAAGAACCCCATCGACTTCC
TGGAGGCCAAGGGCTACAAGGAGGTGAAGAAGGACCTGATCATCAAGCTGCCCAAGTAC
AGCCTGTTCGAGCTGGAGAACGGCCGCAAGCGCATGCTGGCCAGCGCCGGCGAGCTGCA
GAAGGGCAACGAGCTGGCCCTGCCCAGCAAGTACGTGAACTTCCTGTACCTGGCCAGCC
ACTACGAGAAGCTGAAGGGCAGCCCCGAGGACAACGAGCAGAAGCAGCTGTTCGTGGA
GCAGCACAAGCACTACCTGGACGAGATCATCGAGCAGATCAGCGAGTTCAGCAAGCGCG
TGATCCTGGCCGACGCCAACCTGGACAAGGTGCTGAGCGCCTACAACAAGCACCGCGAC
AAGCCCATCCGCGAGCAGGCCGAGAACATCATCCACCTGTTCACCCTGACCAACCTGGGC
GCCCCCGCCGCCTTCAAGTACTTCGACACCACCATCGACCGCAAGCGCTACACCAGCACC
AAGGAGGTGCTGGACGCCACCCTGATCCACCAGAGCATCACCGGTCTGTACGAGACCCGC
ATCGACCTGAGCCAGCTGGGCGGCGACGCGGCCGCACTCGACCTGCAGATGCAAACGTT

ACAGATGAGTCTGTTGACACAACCTTACGTTCAGCCTCGTTTCCCTTGCAAGCGTTACCCG
ACCTTCTCCGCATCCTGCAGAACTCAAAAGACAGCGATCACGAAAACAGAGAAGGTGTTT
TTCAGTGAGTCATTTGATCAAACACGTTGCACGCAGCCTCTCTCGGAAAAGAAGAAGAGG
GTGTTCTTTTTGGACGTTAACCCGCTCTGTTACGAAGGAAGCAAGCCCAGCTTGCGCTCCT
TCGGGCGGTGGCTCTCTCTGTTTCTCCATCAAGTCAGCCTCACTGACCCCGTCATTGCTGT
TATTGATGGAGAAGGAGGCAGCGAGCATCGCAGAAAGTTGCTACCTTCATATAAAGCACAT
AGGAAAAAGTTCATGAGACACATGTCAAGTGGCCATGTTGGGAGGTCTCATCAAGTTATA
AATGATGTTCTTGGAAAATGCAACGTGCCAGTTATAAAGGTTGCTGGTCATGAAGCTGATG
ATGTTGTAGCTACTCTAGCTGGACAAGTTGTCAATAAAGGGTTTCGAGTGGTCATTGGCTC
CCCTGATAAGGATTTTAAGCAGCTTATATCTGAAGATGTGCAAATAGTTATGCCTTTGCCAG
AGTTACAAAGGTGGTCCTTCTACACTCTGAGGCACTACAGGGATCAGTATAATTGTGATCC
AGAATCTGATCTGAGCTTTAGATGCATTGTAGGTGATGAAGTAGACGGCGTTCCTGGTATC
CAGCATTTGGTCCCTAGTTTTGGTCGGAAGACTGCTATGAAACTTATTAAAAAACATGGTT
CCTTGGAAACTTTATTAAATGCGGCTGCAATAAGGACTGTAGGCAGACCATATGCACAGGA
TGCCCTCAAAAACCATGCTGATTACCTTCGGAGAAACTATGAAGTTCTTGCCTTGAAAAGG
GATGTAAATATCCAACTTTATGATGAGTGGTTGGTTAAGAGAGACAATCACAATGATAAAA
CTGCACTATCTTCCTTCTTCAAATATTTGGGAGAAAGTAAGGAGCTCAGTTACAATGGCAG
ACCTATCTCTTACAATGGTCTGCAGAAAAGGCCGGCGGCCACGAAAAAGGCCGGCCAGG
CAAAAAAGAAAAAGCACCACCACCACCACCACTGA

8) SpyCas9-hTdT

ATGGGCAGCAGCCATCATCATCATCATCACAGCAGCGGCCTGGTGCCGCGCGGCAGCCAT
ATGGCTAGCATGACTGGTGGACAGCAAATGGGTCGCGGATCCGAATTCGAGCTCCGTCGA
CAAGCTTGCGGCCGCATGGACAAGAAGTACAGCATCGGCCTGGACATCGGTACCAACAGC
GTGGGCTGGGCCGTGATCACCGACGAGTACAAGGTGCCCAGCAAGAAGTTCAAGGTGCT
GGGCAACACCGACCGCCACAGCATCAAGAAGAACCTGATCGGCGCCCTGCTGTTCGACA
GCGGCGAGACCGCCGAGGCCACCCGCCTGAAGCGCACCGCCCGCCGCCGCTACACCCGC
CGCAAGAACCGCATCTGCTACCTGCAGGAGATCTTCAGCAACGAGATGGCCAAGGTGGAC
GACAGCTTCTTCCACCGCCTGGAGGAGAGCTTCCTGGTGGAGGAGGACAAGAAGCACGA
GCGCCACCCCATCTTCGGCAACATCGTGGACGAGGTGGCCTACCACGAGAAGTACCCCAC
CATCTACCACCTGCGCAAGAAGCTGGTGGACAGCACCGACAAGGCCGACCTGCGCCTGAT
CTACCTGGCCCTGGCCCACATGATCAAGTTCCGCGGCCACTTCCTGATCGAGGGCGACCT
GAACCCCGACAACAGCGACGTGGACAAGCTGTTCATCCAGCTGGTGCAGACCTACAACC
AGCTGTTCGAGGAGAACCCCATCAACGCCAGCGGCGTGGACGCCAAGGCCATCCTGAGC
GCCCGCCTGAGCAAGAGCCGCCGCCTGGAGAACCTGATCGCCCAGCTGCCCGGCGAGAA
GAAGAACGGCCTGTTCGGCAACCTGATCGCCCTGAGCCTGGGCCTGACCCCCAACTTCAA
GAGCAACTTCGACCTGGCCGAGGACGCCAAGCTGCAGCTGAGCAAGGACACCTACGACG
ACGACCTGGACAACCTGCTGGCCCAGATCGGCGACCAGTACGCCGACCTGTTCCTGGCCG
CCAAGAACCTGAGCGACGCCATCCTGCTGAGCGACATCCTGCGCGTGAACACCGAGATCA
CCAAGGCCCCCCTGAGCGCCAGCATGATCAAGCGCTACGACGAGCACCACCAGGACCTG
ACCCTGCTGAAGGCCCTGGTGCGCCAGCAGCTGCCCGAGAAGTACAAGGAGATCTTCTTC
GACCAGAGCAAGAACGGCTACGCCGGCTACATCGACGGCGGCGCCAGCCAGGAGGAGTT
CTACAAGTTCATCAAGCCCATCCTGGAGAAGATGGACGGCACCGAGGAGCTGCTGGTGAA
GCTGAACCGCGAGGACCTGCTGCGCAAGCAGCGCACCTTCGACAACGGCAGCATCCCCC
ACCAGATCCACCTGGGCGAGCTGCACGCCATCCTGCGCCGCCAGGAGGACTTCTACCCCT
TCCTGAAGGACAACCGCGAGAAGATCGAGAAGATCCTGACCTTCCGCATCCCCTACTACG
TGGGCCCCCTGGCCCGCGGCAACAGCCGCTTCGCCTGGATGACCCGCAAGAGCGAGGAG
ACCATCACCCCCTGGAACTTCGAGGAGGTGGTGGACAAGGGCGCCAGCGCCCAGAGCTT

CATCGAGCGCATGACCAACTTCGACAAGAACCTGCCCAACGAGAAGGTGCTGCCCAAGC
ACAGCCTGCTGTACGAGTACTTCACCGTGTACAACGAGCTGACCAAGGTGAAGTACGTGA
CCGAGGGCATGCGCAAGCCCGCCTTCCTGAGCGGCGAGCAGAAGAAGGCCATCGTGGAC
CTGCTGTTCAAGACCAACCGCAAGGTGACCGTGAAGCAGCTGAAGGAGGACTACTTCAA
GAAGATCGAGTGCTTCGACAGCGTGGAGATCAGCGGCGTGGAGGACCGCTTCAACGCCA
GCCTGGGCACCTACCACGACCTGCTGAAGATCATCAAGGACAAGGACTTCCTGGACAACG
AGGAGAACGAGGACATCCTGGAGGACATCGTGCTGACCCTGACCCTGTTCGAGGACCGC
GAGATGATCGAGGAGCGCCTGAAGACCTACGCCCACCTGTTCGACGACAAGGTGATGAA
GCAGCTGAAGCGCCGCCGCTACACCGGCTGGGGCCGCCTGAGCCGCAAGCTTATCAACG
GCATCCGCGACAAGCAGAGCGGCAAGACCATCCTGGACTTCCTGAAGAGCGACGGCTTC
GCCAACCGCAACTTCATGCAGCTGATCCACGACGACAGCCTGACCTTCAAGGAGGACATC
CAGAAGGCCCAGGTGAGCGGCCAGGGCGACAGCCTGCACGAGCACATCGCCAACCTGGC
CGGCAGCCCCGCCATCAAGAAGGGCATCCTGCAGACCGTGAAGGTGGTGGACGAGCTGG
TGAAGGTGATGGGCCGCCACAAGCCCGAGAACATCGTGATCGAGATGGCCCGCGAGAAC
CAGACCACCCAGAAGGGCCAGAAGAACAGCCGCGAGCGCATGAAGCGCATCGAGGAGG
GCATCAAGGAGCTGGGCAGCCAGATCCTGAAGGAGCACCCCGTGGAGAACACCCAGCTG
CAGAACGAGAAGCTGTACCTGTACTACCTGCAGAACGGCCGCGACATGTACGTGGACCAG
GAGCTGGACATCAACCGCCTGAGCGACTACGACGTGGACCACATCGTGCCCCAGAGCTTC
CTGAAGGACGACAGCATCGACAACAAGGTGCTGACCCGCAGCGACAAGAACCGCGGCA
AGAGCGACAACGTGCCCAGCGAGGAGGTGGTGAAGAAGATGAAGAACTACTGGCGCCA
GCTGCTGAACGCCAAGCTGATCACCCAGCGCAAGTTCGACAACCTGACCAAGGCCGAGC
GCGGCGGCCTGAGCGAGCTGGACAAGGCCGGCTTCATCAAGCGCCAGCTGGTGGAGACC
CGCCAGATCACCAAGCACGTGGCCCAGATCCTGGACAGCCGCATGAACACCAAGTACGA
CGAGAACGACAAGCTGATCCGCGAGGTGAAGGTGATCACCCTGAAGAGCAAGCTGGTGA
GCGACTTCCGCAAGGACTTCCAGTTCTACAAGGTGCGCGAGATCAACAACTACCACCACG
CCCACGACGCCTACCTGAACGCCGTGGTGGGCACCGCCCTGATCAAGAAGTACCCCAAGC
TGGAGAGCGAGTTCGTGTACGGCGACTACAAGGTGTACGACGTGCGCAAGATGATCGCCA
AGAGCGAGCAGGAGATCGGCAAGGCCACCGCCAAGTACTTCTTCTACAGCAACATCATGA
ACTTCTTCAAGACCGAGATCACCCTGGCCAACGGCGAGATCCGCAAGCGCCCCCTGATCG
AGACCAACGGCGAGACCGGCGAGATCGTGTGGGACAAGGGCCGCGACTTCGCCACCGTG
CGCAAGGTGCTGAGCATGCCCCAGGTGAACATCGTGAAGAAGACCGAGGTGCAGACCGG
CGGCTTCAGCAAGGAGAGCATCCTGCCCAAGCGCAACAGCGACAAGCTGATCGCCCGCA
AGAAGGACTGGGACCCCAAGAAGTACGGCGGCTTCGACAGCCCCACCGTGGCCTACAGC
GTGCTGGTGGTGGCCAAGGTGGAGAAGGGCAAGAGCAAGAAGCTGAAGAGCGTGAAGG
AGCTGCTGGGCATCACCATCATGGAGCGCAGCAGCTTCGAGAAGAACCCCATCGACTTCC
TGGAGGCCAAGGGCTACAAGGAGGTGAAGAAGGACCTGATCATCAAGCTGCCCAAGTAC
AGCCTGTTCGAGCTGGAGAACGGCCGCAAGCGCATGCTGGCCAGCGCCGGCGAGCTGCA
GAAGGGCAACGAGCTGGCCCTGCCCAGCAAGTACGTGAACTTCCTGTACCTGGCCAGCC
ACTACGAGAAGCTGAAGGGCAGCCCCGAGGACAACGAGCAGAAGCAGCTGTTCGTGGA
GCAGCACAAGCACTACCTGGACGAGATCATCGAGCAGATCAGCGAGTTCAGCAAGCGCG
TGATCCTGGCCGACGCCAACCTGGACAAGGTGCTGAGCGCCTACAACAAGCACCGCGAC
AAGCCCATCCGCGAGCAGGCCGAGAACATCATCCACCTGTTCACCCTGACCAACCTGGGC
GCCCCCGCCGCCTTCAAGTACTTCGACACCACCATCGACCGCAAGCGCTACACCAGCACC
AAGGAGGTGCTGGACGCCACCCTGATCCACCAGAGCATCACCGGTCTGTACGAGACCCGC
ATCGACCTGAGCCAGCTGGGCGGCGACGCGGCCGCACTCGACCTGCAGATGGATCCACCA
CGAGCGTCCCACTTGAGCCCTCGGAAGAAGAGACCCCGGCAGACGGGTGCCTTGATGGC
CTCCTCTCCTCAAGACATCAAATTTCAAGATTTGGTCGTCTTCATTTTGGAGAAGAAAATG
GGAACCACCCGCAGAGCGTTCCTCATGGAGCTGGCCCGCAGGAAAGGGTTCAGGGTTGA
AAATGAGCTCAGTGATTCTGTCACCCACATTGTAGCAGAGAACAACTCGGGTTCGGATGT
TCTGGAGTGGCTTCAAGCACAGAAAGTACAAGTCAGCTCACAACCAGAGCTCCTCGATGT

CTCCTGGCTGATCGAATGCATAGGAGCAGGGAAACCGGTGGAAATGACAGGAAAACACC
AGCTTGTTGTGAGAAGAGACTATTCAGATAGCACCAACCCAGGCCCCCGAAGACTCCAC
CAATTGCTGTACAAAAGATCTCCCAGTATGCGTGTCAGAGAAGAACCACTTTAAACAACT
GTAACCAGATATTCACGGATGCCTTTGATATACTGGCTGAAAACTGTGAGTTTAGAGAAAA
TGAAGACTCCTGTGTGACATTTATGAGAGCAGCTTCTGTATTGAAATCTCTGCCATTCACA
ATCATCAGTATGAAGGACACAGAAGGAATTCCCTGCCTGGGGTCCAAGGTGAAGGGTATC
ATAGAGGAGATTATTGAAGATGGAGAAAGTTCTGAAGTTAAAGCTGTGTTAAATGATGAAC
GATATCAATCCTTCAAACTCTTTACTTCTGTATTTGGAGTGGGGCTGAAGACTTCTGAGAA
GTGGTTCAGGATGGGTTTCAGAACTCTGAGTAAAGTAAGGTCGGACAAAAGCCTGAAATT
TACACGAATGCAGAAAGCAGGATTTCTGTATTATGAAGACCTTGTCAGCTGTGTGACCAGG
GCAGAAGCAGAGGCCGTCAGTGTGCTGGTTAAAGAGGCTGTCTGGGCATTTCTTCCGGAT
GCTTTCGTCACCATGACAGGAGGGTTCCGGAGGGGTAAGAAGATGGGGCATGATGTAGAT
TTTTTAATTACCAGCCCAGGATCAACAGAGGATGAAGAGCAACTTTTACAGAAAGTGATG
AACTTATGGGAAAAGAAGGGATTACTTTTATATTATGACCTTGTGGAGTCAACATTTGAAA
AGCTCAGGTTGCCTAGCAGGAAGGTTGATGCTTTGGATCATTTTCAAAAGTGCTTTCTGAT
TTTCAAATTGCCTCGTCAAAGAGTGGACAGTGACCAGTCCAGCTGGCAGGAAGGAAAGA
CCTGGAAGGCCATCCGTGTGGATTTAGTTCTGTGCCCCTACGAGCGTCGTGCCTTTGCCCT
GTTGGGATGGACTGGCTCCCGGCAGTTTGAGAGAGACCTCCGGCGCTATGCCACACATGA
GCGGAAGATGATTCTGGATAACCATGCTTTATATGACAAGACCAAGAGGATATTCCTCAAA
GCAGAAAGTGAAGAAGAAATTTTTGCGCATCTGGGATTGGATTATATTGAACCGTGGGAA
AGAAATGCCCTGCAGAAAAGGCCGGCGGCCACGAAAAAGGCCGGCCAGGCAAAAAAGA
AAAAGCACCACCACCACCACCACTGA

These are DNA sequences of CRISPR PLUS proteins. Common sequence of SpyCas9 is colored by pale brown. Black sequences at 5'-end and 3'-end indicate sequence following RBS (Ribosomal binding site) and NLS (Nuclear localization signal) linked 6xHis tag respectively. DNA sequences encoding proteins fused to SpyCas9 are presented by different colors; RecJ (red), GFP (green), RecE (light green), T5 (light blue), lambda (pink), mungbean (dark blue) and hTdT (violet).
