# Supplementary material for: Author Correction: Enhanced genome editing efficiency of CRISPR PLUS: Cas9 chimeric fusion proteins
Source: Sci Rep. 2021 Sep 10;11:18427. doi: 10.1038/s41598-021-98186-3 (PMC8433393; doi:10.1038/s41598-021-98186-3)

Supplementary Figures

S1 Fig. SDS-PAGE of SpyCas9 and the fusion proteins.

An SDS-PAGE gel was loaded with 8 μg of each protein after purification. The left- and rightmost lanes display molecular weight size markers, indicated in kDa. The gel image was stained with Coomassie Brilliant Blue. The molecular weights in kDa of SpyCas9, SpyCas9-RecJ, SpyCas9-GFP, SpyCas9-RecE, SpyCas9-T5, SpyCas9-Lambda, SpyCas9-mungbean, and SpyCas9-hTdT were 167, 230, 194, 263, 198, 193, 207, and 225, respectively. The protein yields during the purification process are given in S1 Text.

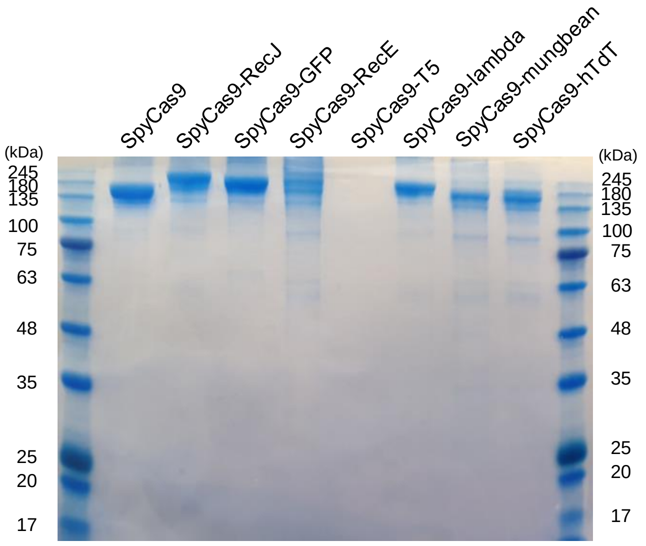

S2 Fig. Functional validation of the exonuclease activity of the RecJ moiety in SpyCas9-RecJ and green fluorescence in SpyCas9-GFP. (A) Exonuclease activity of C9R. RecJ exonuclease activity of the SpyCas9-RecJ fusion protein was tested on ssDNA and dsDNA in the presence or absence of magnesium ions. The DNA band intensity was diminished only when C9R protein acted with ssDNA in the presence of magnesium ions (right most lane). (B) Emission of green light by C9G. C9G showed a major excitation peak at a wavelength of 395 nm and a minor one at 475 nm, and an emission peak was observed at 509 nm (solid lines). C9 displayed only basal level of absorption and emission (broken lines).

a

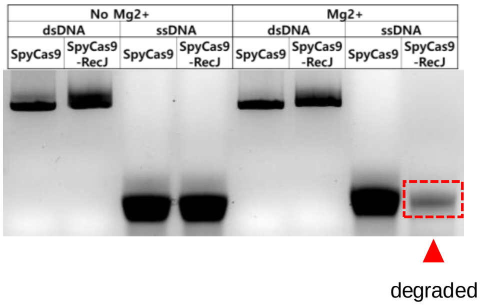

b

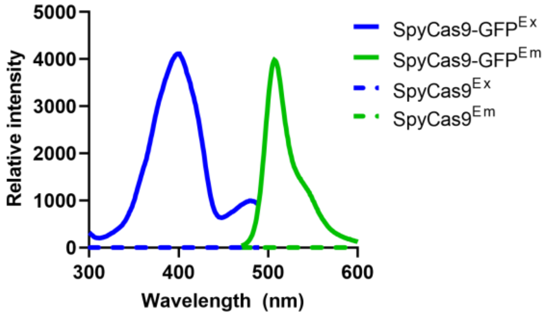

S3 Fig. The off-target effects of SpyCas9-RecJ were not significantly different from those of Cas9. For each target gene, five putative off-target sites were identified using Cas-OFFinder and subjected to NGS deep sequencing. The results show that the rates of mutations in the potential off-target sites were not significantly different from the conventional NGS sequencing error rate (0.01–0.1%).

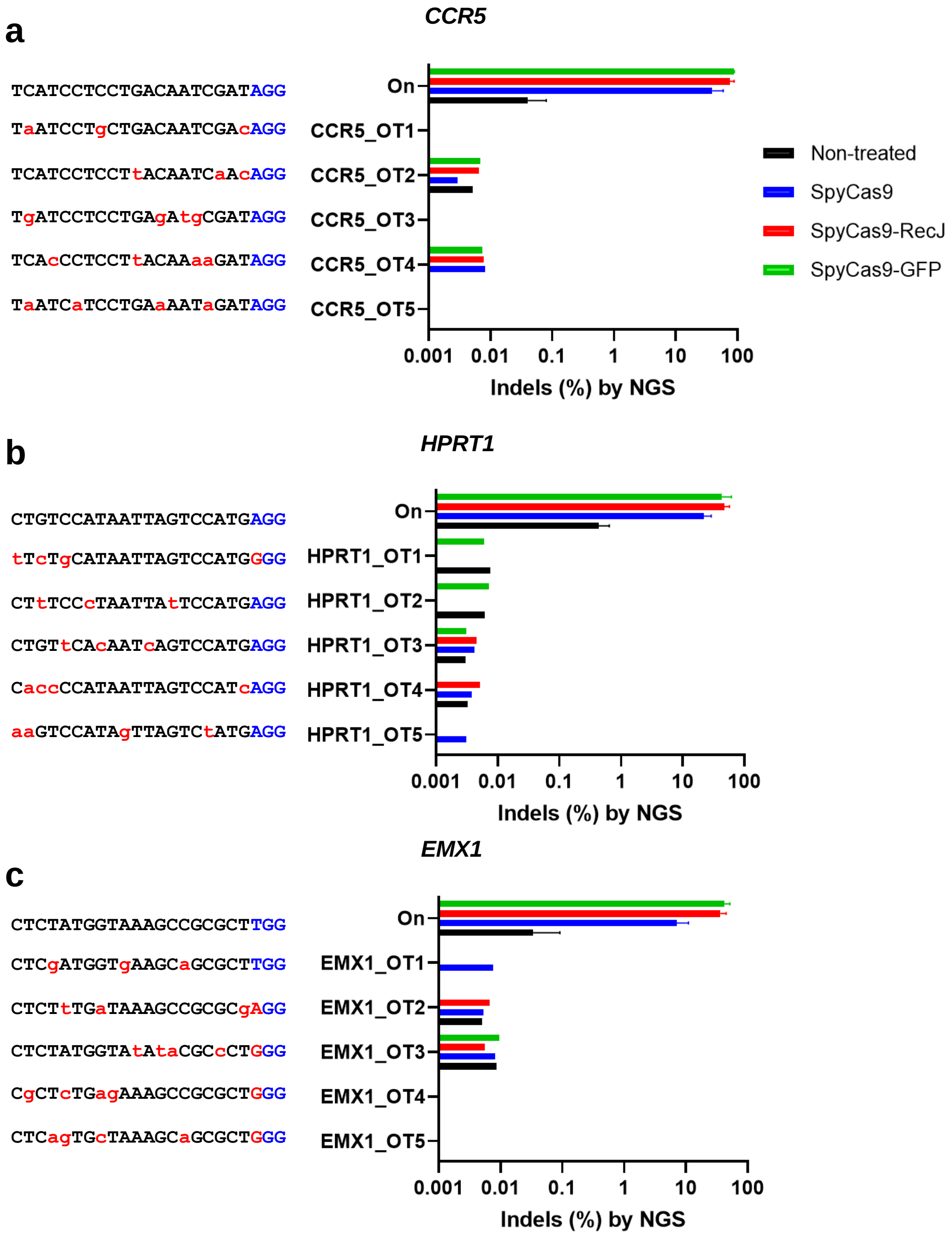

S4 Fig. DNA replacement activity in HEK293T cells.
HEK293T cells treated with C9 protein only, C9, C9R, and C9G (from left to right, lanes 1-4) targeting *CCR5*, *HPRT1*, and *EMX1* were harvested, and the DNA spanning the protospacer site was amplified, digested with *NdeI*, and resolved on an agarose gel (left to right). The substrates and cleaved products are indicated by red and blue triangles, respectively. The HDR activity was digitized and analyzed using ImageJ. HDR percentage is indicated bottom of each gel image.

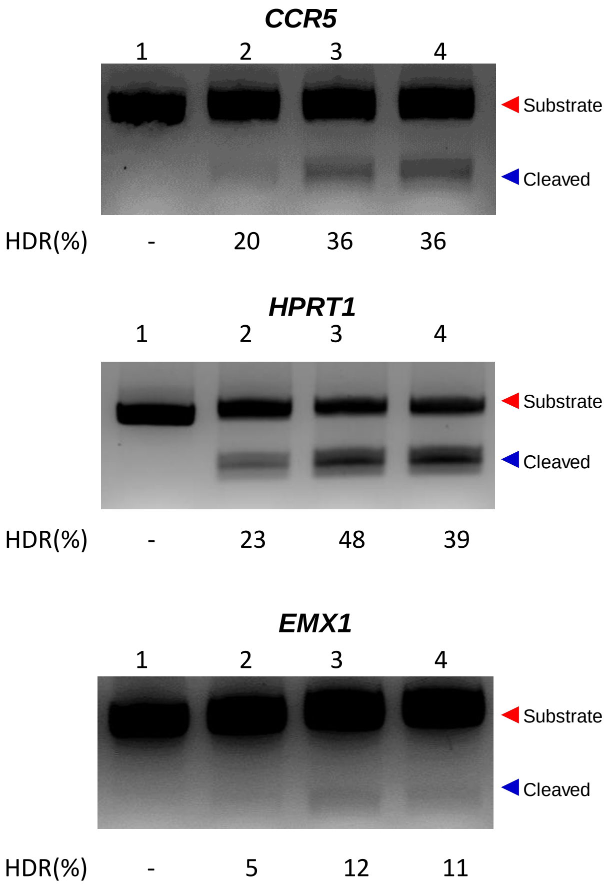

S5 Fig. Screening for sgRNAs targeting immune regulator genes. The genome editing efficiency of C9 and C9R was evaluated for two different sgRNAs for each of the target genes (*B2M*, *CIITA*, *CTLA4*, and *PDCD-1*). For each tested pair, an sgRNA showing greater indel efficiency (downward arrowheads) was chosen for the editing experiments with iPSCs. NT, non-treated cells as a negative control.

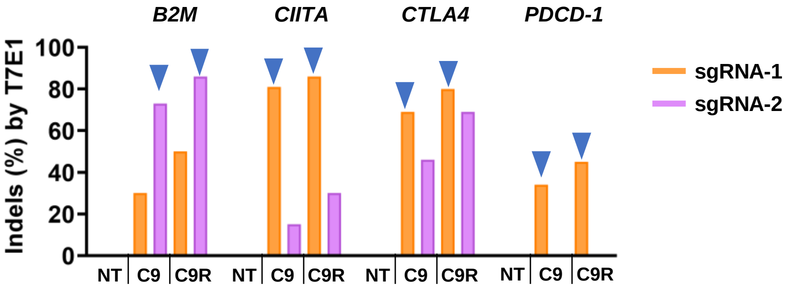

S6 Fig. T7E1 analysis of the indel efficiency after transfection of preassembled RNP for the four target genes. Three sets of four different RNPs, each pre-assembled with a different sgRNA (targeting *B2M*, *CIITA*, *CTLA4*, or *PDCD-1*) were simultaneously transfected into HEK293T cells and incubated for 3 d, and the T7E1 analysis was performed to determine the indel efficiencies. NT and M represent the non-treated control and DNA molecular weight marker, respectively. The substrates and cleaved products are indicated by red and blue triangles, respectively. The indel efficiency was digitized and analyzed using ImageJ. Indel efficiency in percentage is indicated bottom of each gel image.

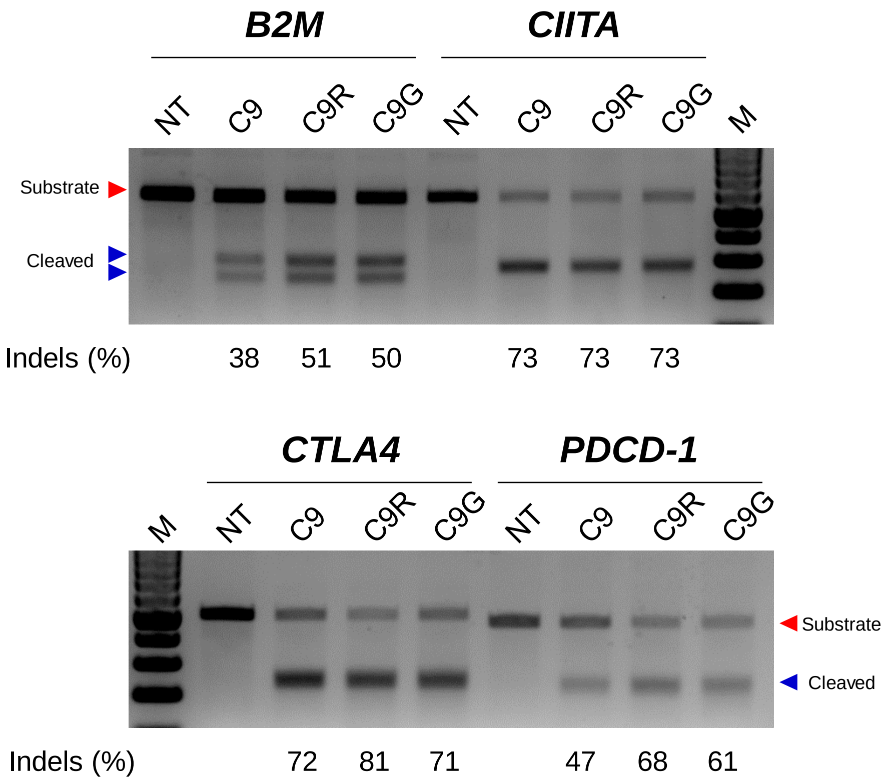

S7 Fig. Comparison of C9 and C9R activity in plant cells. Each C9 or C9R RNP preassembled with sgRNA targeting α-1,3-fucosyltransferase 1 (FucT13-1) was transfected into protoplasts derived from 4-week-old *N. benthamiana* leaves. More than 2.6-fold increase in indel efficiency of C9R relative to C9 was observed in targeted deep sequencing analysis. The error bars stand for standard deviations (SD), generated by a Nested *t*-test from GraphPad.

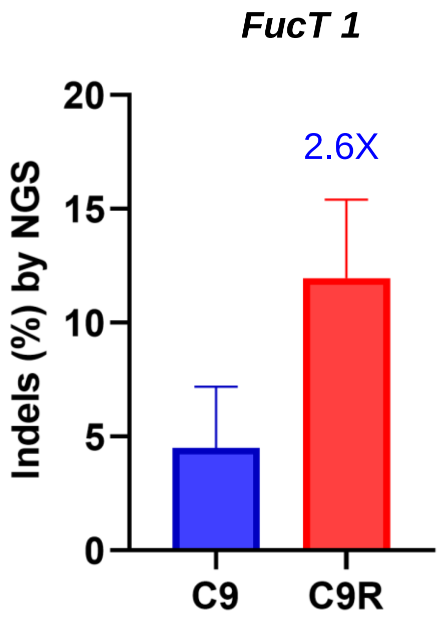

S8-S13 Fig. The full size of images, which were used in Fig 1B, S2, S4 and S6.

S8 Fig. The full size of image, which was used in Fig 1B

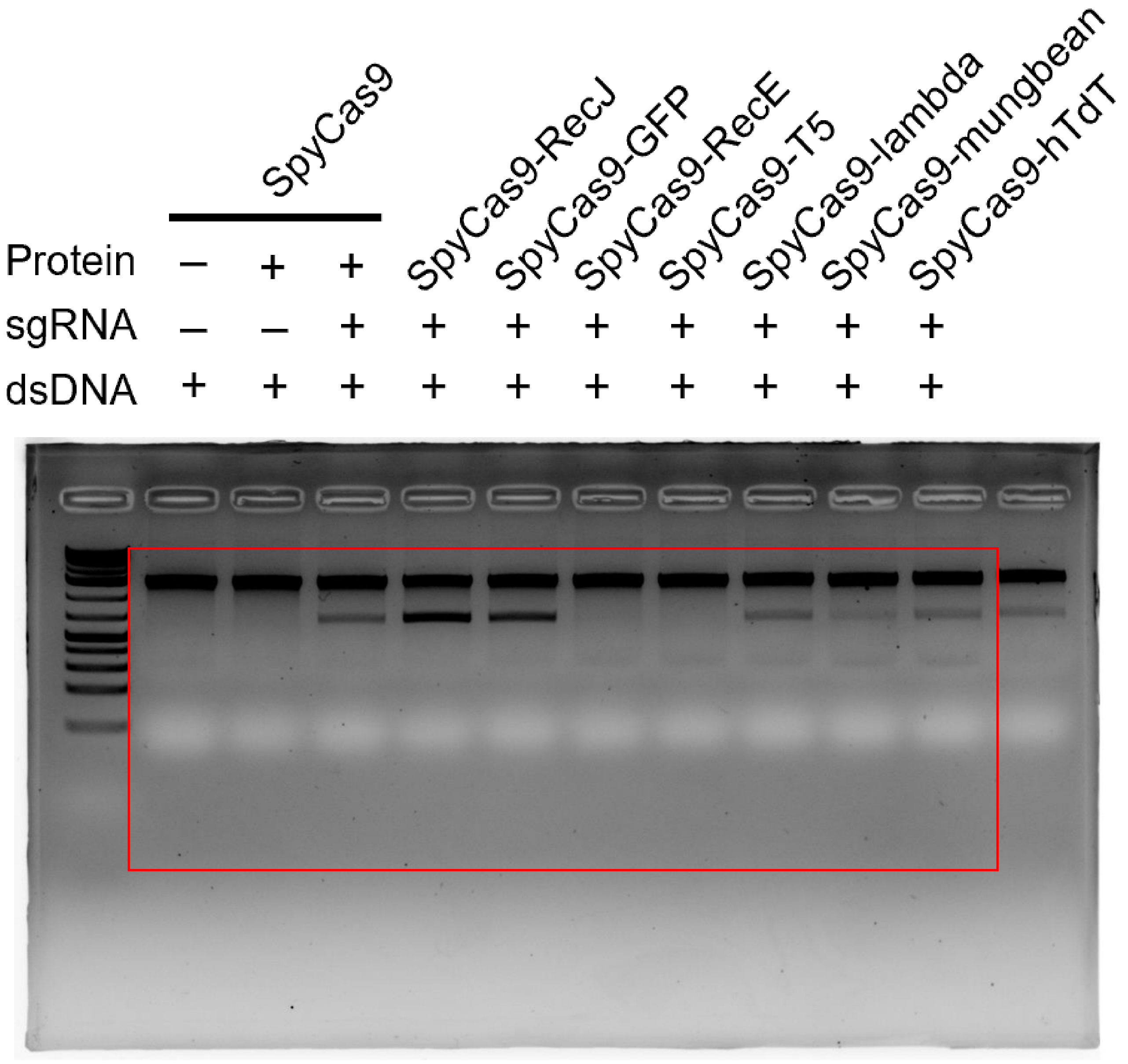

S9 Fig. The full size of image, which was used in S2 Fig.

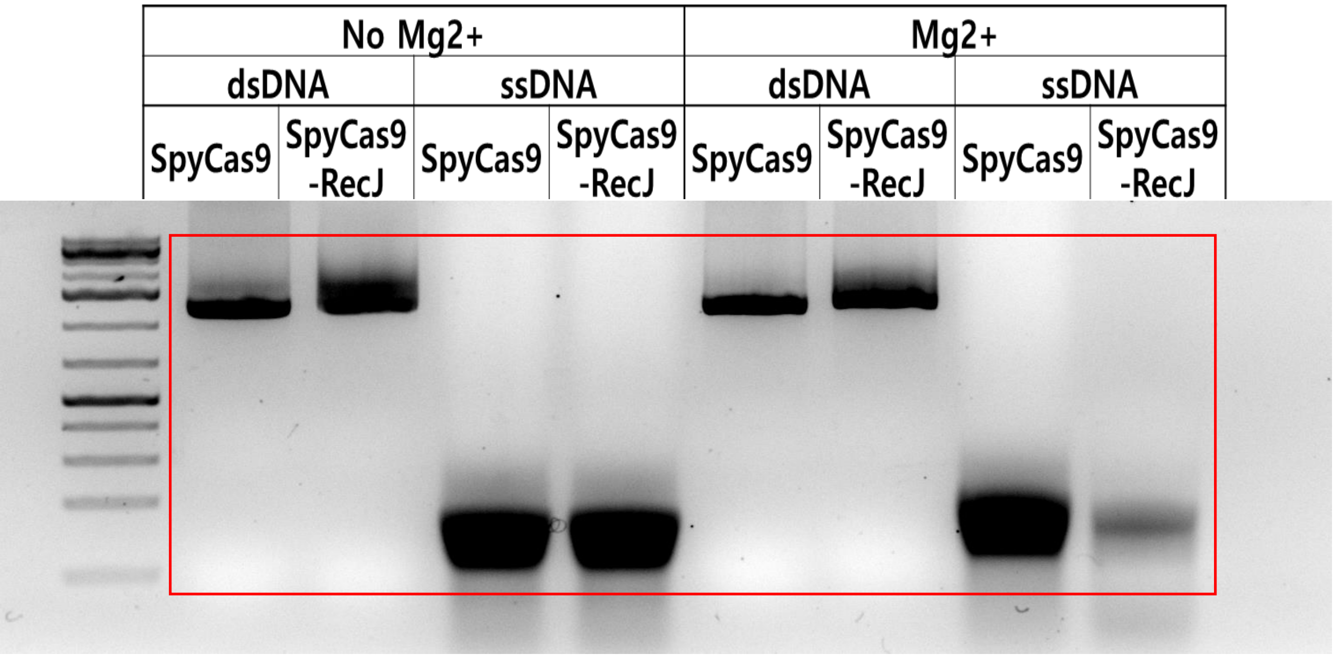

S10 Fig. The full size of image, which was used in S4 Fig.

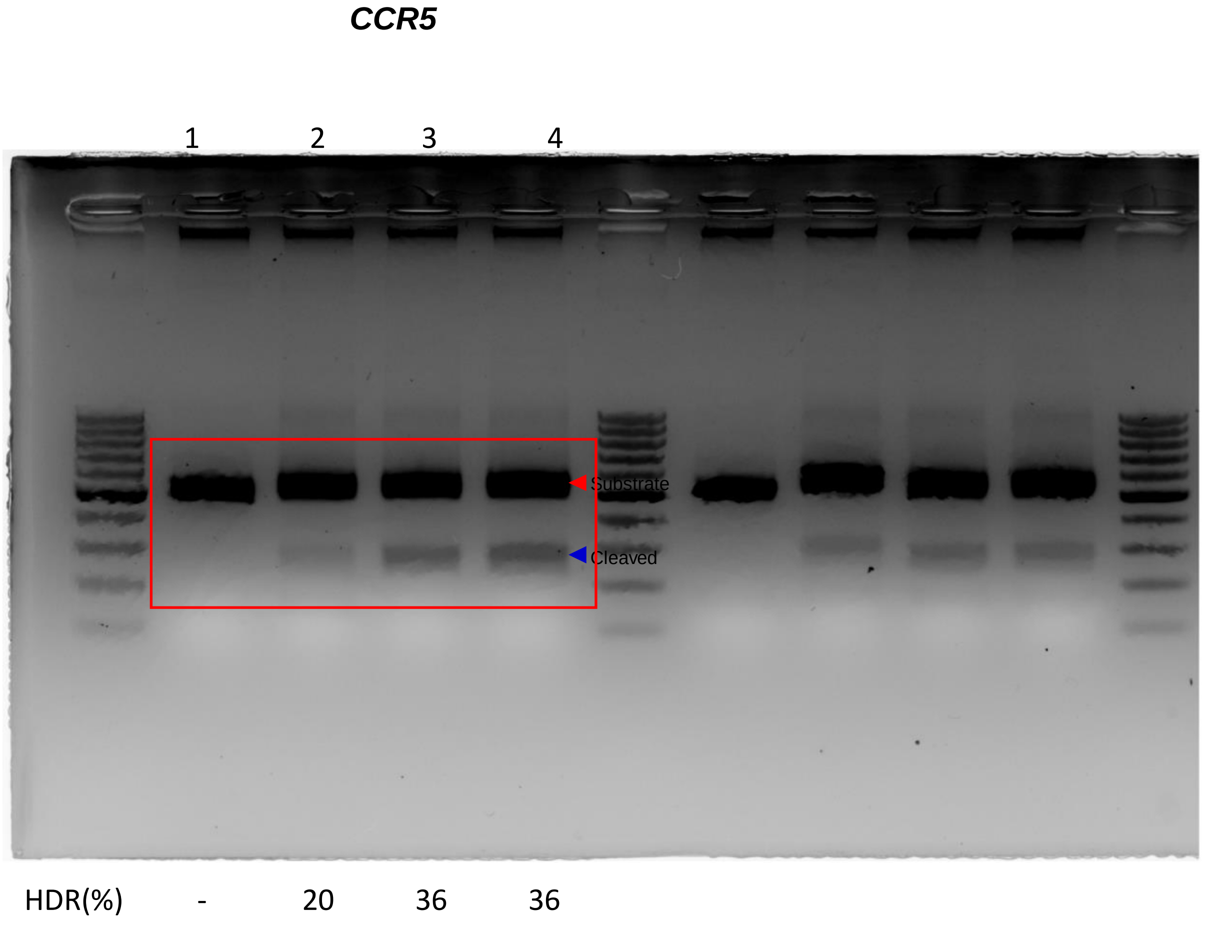

S11 Fig. The full size of image, which was used in S4 Fig.

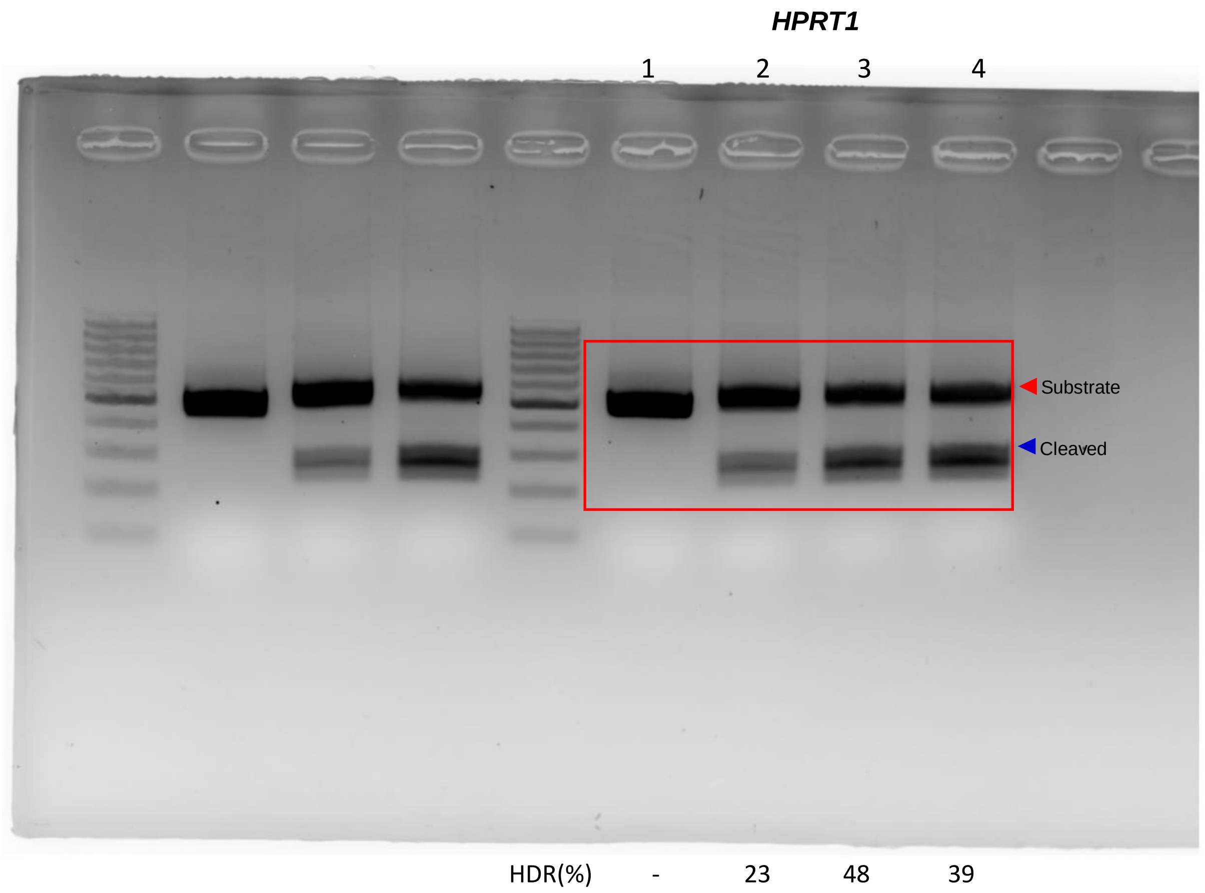

S12 Fig. The full size of image, which was used in S4 Fig.

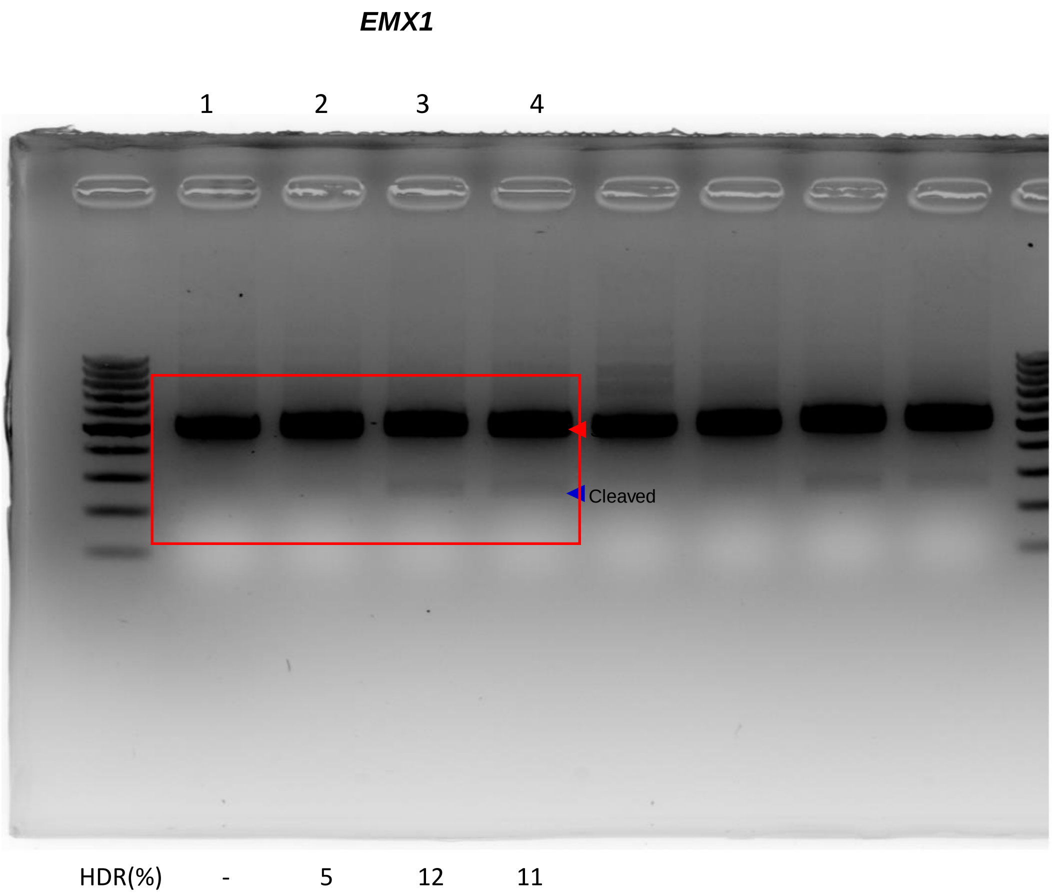

S13 Fig. The full size of image, which was used in S6 Fig.

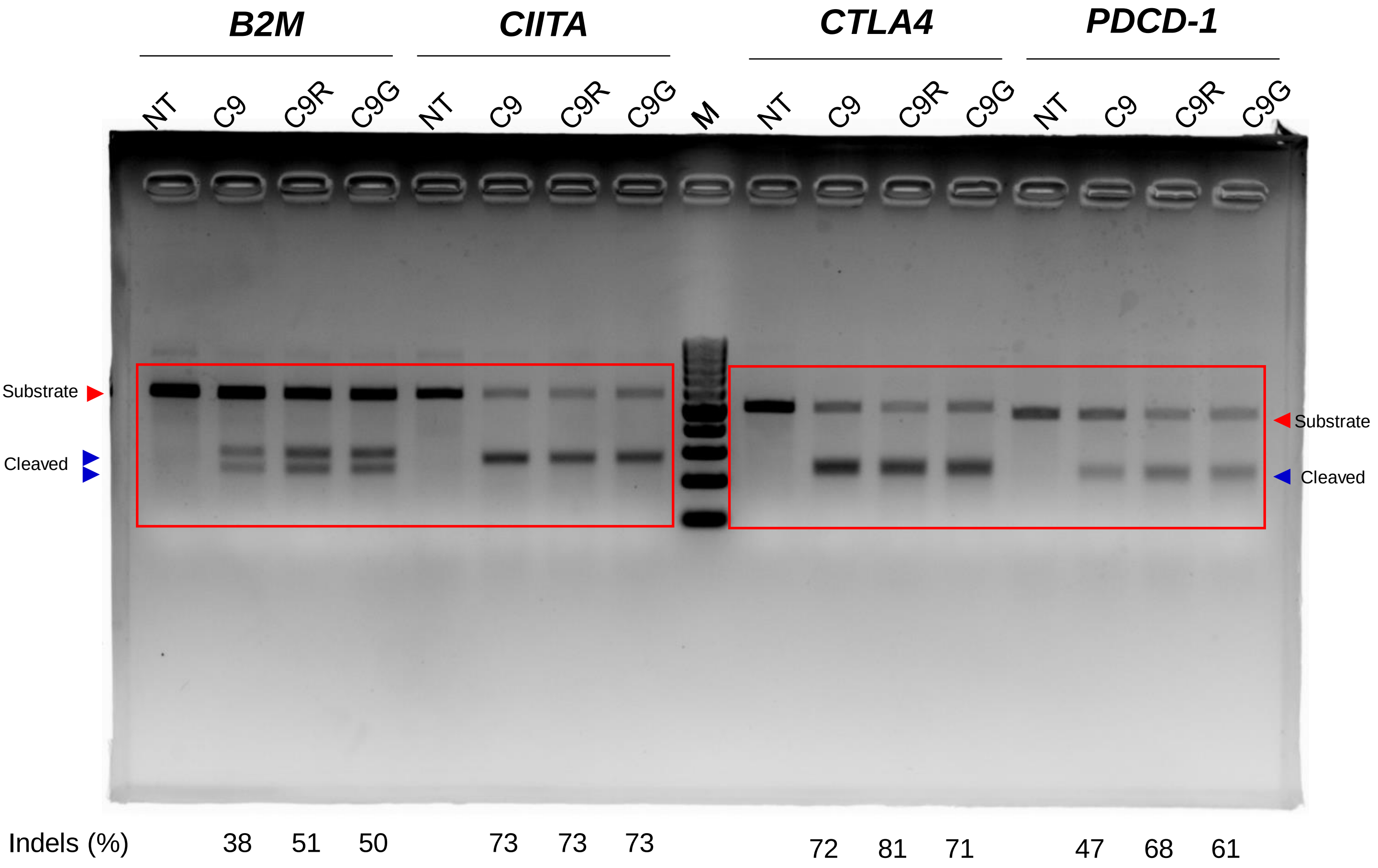

Supplement: Supplementary file 1 — Supplementary Information. [file 41598_2021_98186_MOESM1_ESM.pdf]
